# Adiponectin exerts sex-dependent effects on lipid, amino acid, and glucose metabolism during caloric restriction

**DOI:** 10.1371/journal.pbio.3003821

**Published:** 2026-06-18

**Authors:** Yoshiko M. Ikushima, Kuan-Chan Chen, Richard J. Sulston, Domenico Mattiucci, Eleanor J. Brain, Stefanie A. Fung Xin Zi, Karla J. Suchacki, Benjamin J. Thomas, Andrea Lovdel, Matthew Bennett, Hiroshi Kobayashi, Phillip D. Whitfield, Keiyo Takubo, Andrew H. Baker, Nicholas M. Morton, Robert K. Semple, William P. Cawthorn

**Affiliations:** 1 Institute for Neuroscience and Cardiovascular Research, The University of Edinburgh, Edinburgh BioQuarter, Edinburgh, United Kingdom; 2 Department of Medical Science and Innovation, SiRIUS Institute of Medical Research, Tohoku University, Sendai, Japan; 3 Division of Endocrinology and Metabolism, Department of Internal Medicine, Tri-Service General Hospital, National Defense Medical University, Taipei, Taiwan; 4 Scotland’s Rural College, The Roslin Institute, Easter Bush Campus, Edinburgh, United Kingdom; 5 Department of Cell Fate Biology and Stem Cell Medicine, Tohoku University Graduate School of Medicine, Sendai, Japan; 6 Department of Stem Cell Biology, National Institute of Global Health and Medicine, Japan Institute for Health Security (JIHS), Tokyo, Japan; 7 Kanagawa Institute of Industrial Science and Technology (KISTEC), Kawasaki, Japan; 8 Division of Biomedical Sciences, University of the Highlands and Islands, Centre for Health Sciences, Inverness, United Kingdom; 9 Institute of Infection, Immunity & Inflammation, University of Glasgow, Glasgow, United Kingdom; 10 Centre for Systems Health and Integrated Metabolic Research, Department of Biosciences, School of Science and Technology, Nottingham Trent University, Nottingham, United Kingdom; Columbia University, UNITED STATES OF AMERICA

## Abstract

Adiponectin is the most abundant hormone in the circulation. Plasma adiponectin decreases in obesity but increases in leanness, including during caloric restriction (CR) in animals and humans. In obesity, adiponectin deficiency promotes cardiometabolic dysfunction. In contrast, the roles of adiponectin in CR, when it is at its highest, are largely unknown. To address this, we studied global adiponectin knockout (KO) in male and female mice fed either *ad libitum* (AL) or a 30% CR diet from 9–13 weeks of age. We show that adiponectin KO did not alter CR effects on body mass, body composition, or energy expenditure. However, KO unexpectedly decreased blood glucose levels during CR, both with fasting and following an oral glucose challenge. This is opposite to the effects of adiponectin deficiency during AL feeding or obesity and occurred without changes in insulin concentrations or sensitivity. Moreover, adiponectin KO augmented CR-induced increases in plasma fatty acids in both sexes and, in males only, impaired systemic triglyceride clearance on both AL and CR diets. These effects on lipid metabolism were associated with sex- and diet-specific KO effects on white adipose tissue, including altered adipocyte size and expression of key regulators of adipocyte lipid metabolism. Indirect calorimetry further revealed that adiponectin KO alters the shifts between carbohydrate and lipid utilization that occur during transitions between fed and fasted states. To determine potential molecular mechanisms, we investigated effects of adiponectin KO on the liver, a major adiponectin target that plays key roles entraining metabolism to nutritional state. Hepatic transcriptomics revealed that, in both sexes, adiponectin KO upregulates sterol and fatty acid synthesis genes under AL while increasing amino acid catabolic genes during CR. However, the latter occurred without altering plasma or hepatic amino acid concentrations. Together, our findings suggest that adiponectin exerts sexually dimorphic effects on glucose, lipid, and amino acid metabolism during CR, in whole or in part through effects on the liver. Thus, the roles adiponectin in CR differ markedly from its widely reported functions in obesity, insulin resistance, and other pathological states.

## Abstract 2

Adiponectin is the most abundant mammalian hormone, decreasing in obesity and increasing in leanness, including during caloric restriction, but its role in this process is mostly unknown. This study shows that absence of adiponectin lowers glucose, and affects lipid use and amino acid metabolism during caloric restriction in a sex-specific manner.

## 1. Introduction

Adiponectin is a multimeric secreted protein that is among the most abundant in the plasma, with typical circulating concentrations of 1–20 mg/L [1,2]. Although it is expressed almost exclusively by adipocytes, plasma adiponectin correlates inversely with adiposity [1,2]. Low plasma adiponectin in humans is also a biomarker for increased cardiometabolic risk [1,2]; hence, intensive research over the past three decades has focused on a putative mediating role for low adiponectin in adverse cardiometabolic outcomes. Much of the direct support for this comes from murine models. In mice, adiponectin deficiency usually exacerbates obesity-induced glucose intolerance, insulin resistance, and ectopic lipid accumulation in organs such as liver, pancreas, and skeletal muscle (SkM) [2]. Conversely, interventions that increase adiponectin generally protect against obesity-associated metabolic dysfunction [1,2].

The focus on adiponectin’s role in cardiometabolic disease is understandable given the biomedical imperative. However, it seems implausible that any such role explains the apparently strong selective pressure that has led to adiponectin’s high expression and phylogenetic conservation. Adiponectin is dispensable for survival under conventional animal house conditions [1,2], but other clues to its fundamental evolutionary role may lie in the conditions under which it is most highly expressed. These include, most prominently, chronic caloric restriction (CR), defined by a sustained but non-lethal decrease in calorie intake [3,4]—a key selective pressure over evolutionary time [3]. The ability to adapt to CR by decreasing energy expenditure, thus conserving resources, is likely to have been crucial for survival in the face of privation [3–5].

The need to minimize negative energy balance makes the increase in adiponectin production during CR even more remarkable, given that adiponectin is already highly abundant. This implies an important role for adiponectin in adaptation to CR. However, only three studies to date have directly sought evidence for this [6–8]. They suggest roles for adiponectin in ischemic protection and bone loss, but did not examine metabolic adaptations. Other pertinent findings are that, during fasting, mice with transgenic suppression or deletion of adiponectin exhibit greater weight and fat loss than control mice [9,10]; however, these mice were not subjected to sustained CR. Thus, whether adiponectin directly influences CR’s metabolic impact remains unknown.

Importantly, investigating the role of adiponectin, if any, in adaptation to CR is motivated by more than evolutionary curiosity: CR extends healthspan in species ranging from yeast to humans [4] and is implemented in various ways in weight loss and healthy living regimens. Understanding the molecular mediators of the beneficial effects of CR may offer opportunities to mimic these pharmacologically.

Herein, we investigated the role of adiponectin in CR by investigating male and female adiponectin KO mice and their wild-type (WT) littermates during *ad libitum* (AL) and CR feeding. Our study reveals that adiponectin has distinct, unexpected, and often sexually dimorphic effects on lipid, amino acid, and glucose metabolism under AL and CR. These findings have implications for understanding adiponectin’s evolutionary function and its contribution to CR’s therapeutic benefits.

## 2. Results

### 2.1. Plasma adiponectin increases during CR

**Fig 1:**
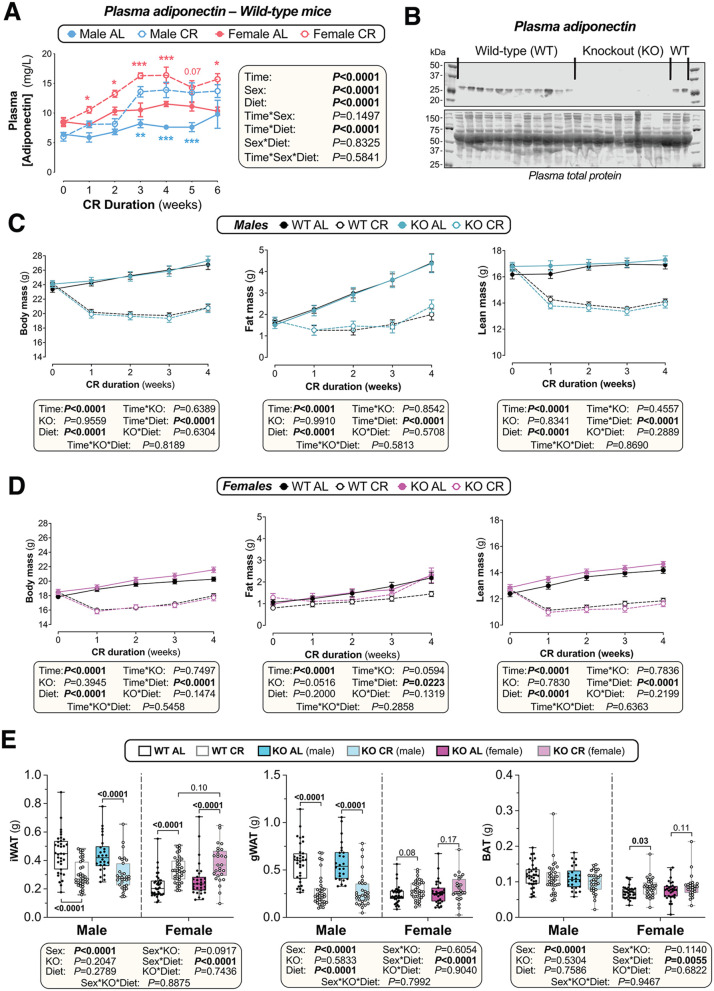
Adiponectin KO does not alter CR’s effects on body mass, fat mass, or lean mass. Male and female WT and *Adipoq* KO mice were fed *ad libitum* (AL) or a 30% CR diet from 9 to 15 weeks of age (0–6 weeks of CR) **(A)** or 9–13 weeks of age (0–4 weeks of CR) **(C–F)**. **(A)** Plasma adiponectin concentrations were determined by ELISA in tail vein blood sampled weekly from male and female WT mice. Data are mean ± SEM of 5 mice per group for weeks 0–6 plus a further 5 mice per group (CR males, AL or CR females) or 6 mice per group (AL males) for weeks 0–2. **(B)** Immunoblot of adiponectin in plasma from WT and adiponectin KO mice. Equal loading was confirmed by total protein stain*.* **(C, D)** Each week male (C) and female (D) mice were weighed (left panel) and TD-NMR used to determine total fat mass (middle panel) and lean mass (right panel). **(E)** Masses of iWAT (inguinal WAT), gWAT (gonadal WAT) and BAT were recorded at necropsy (13 weeks of age). Data are shown as mean ± SEM (C, D) or as box-and-whisker plots (E) of the following numbers of mice per group: *male WT AL*, *n* = 24 (C) or 35 (E); *male WT CR*, *n* = 24 (C) or 35 (E); *male KO AL*, *n* = 18 (C) or 26 (E); *male KO CR*, *n* = 22 (C) or 31 (E); *female WT AL*, *n* = 23 (D) or 36 (E); *female WT CR*, *n* = 25 (D) or 37 (E); *female KO AL*, *n* = 22 (D) or 31 (E); *female KO CR*, *n* = 20 (D) or 27 (E). Significant effects of diet, time, sex, and/or genotype, and interactions thereof, were determined by mixed-effects models (A) or 3-way ANOVA (C–F); *P values* for each variable and interaction are shown beneath each graph. In (A), 2-way ANOVA with Šidák’s multiple comparisons test was used to identify significant diet effects at each time point within each sex. Significant differences between AL and CR mice are indicated by * *(P* < 0.05), ** (*P* < 0.01), or *** (*P* < 0.001); blue asterisks are for males and red asterisks are for females. In (E), significant diet effects (within each sex and genotype) or genotype effects (within each sex and diet) were determined by 2-way ANOVA with Fisher’s LSD test; *P* values for each comparison are shown on each graph. The underlying data for this figure can be found in the S1 Data file and S1 Raw Images.

We first tested if our CR protocol increases circulating adiponectin in WT mice. Compared to AL controls, 30% CR increased plasma adiponectin by ~50% in females and ~70% in males from weeks 3 to 6 of CR (Fig 1A). Females had higher adiponectin than males, irrespective of diet; however, the overall CR effect was similar between the sexes.

### 2.2. Adiponectin KO does not alter CR’s effects on body composition

We next generated adiponectin global KO mice by deletion of exon 3, which encodes the globular domain (S1A Fig). We first confirmed adiponectin KO by the absence of plasma adiponectin (Fig 1B). WT and KO mice then underwent AL or CR feeding from 9 to 13 weeks of age and adiponectin KO was further confirmed by analysis of adiponectin expression in white adipose tissue (WAT). KO mice retained some expression of exons 1–2 of the *Adipoq* transcript but exon 3 was undetectable (S1B and S1C Fig), resulting in a complete lack of adiponectin protein in WAT (S1D and S1E Fig). In WT mice, adiponectin protein was higher in females versus males and increased with CR in females only (S1D and S1E Fig).

We next investigated if KO altered CR’s metabolic effects, beginning with body mass and composition; males and females were studied separately given our prior observation that CR exerts sexually dimorphic effects [5]. Consistent with previous findings [5], CR decreased body mass, fat mass, and lean mass in WT males whereas in females only body and lean masses decreased (Fig 1C and 1D). Adiponectin KO did not affect body mass, fat mass or lean mass, irrespective of sex or diet, nor, importantly, did it alter CR’s effects on these parameters (Fig 1C and 1D).

Decreased adiposity is critical to CR’s overall metabolic benefits [5]. As previously observed [5], WAT in the inguinal (iWAT) and gonadal (gWAT) depots was decreased by CR in WT males but increased by CR in WT females (Fig 1E and 1F). Adiponectin KO did not affect iWAT, gWAT, or brown adipose tissue (BAT) masses in either sex, irrespective of diet, nor did it alter CR’s effects on the masses of these depots (Fig 1E). Thus, adiponectin does not influence CR’s effects on body mass, fat mass, lean mass, or regional adiposity in either sex.

### 2.3. CR-induced decreases in blood glucose are enhanced by adiponectin KO

**Fig 2:**
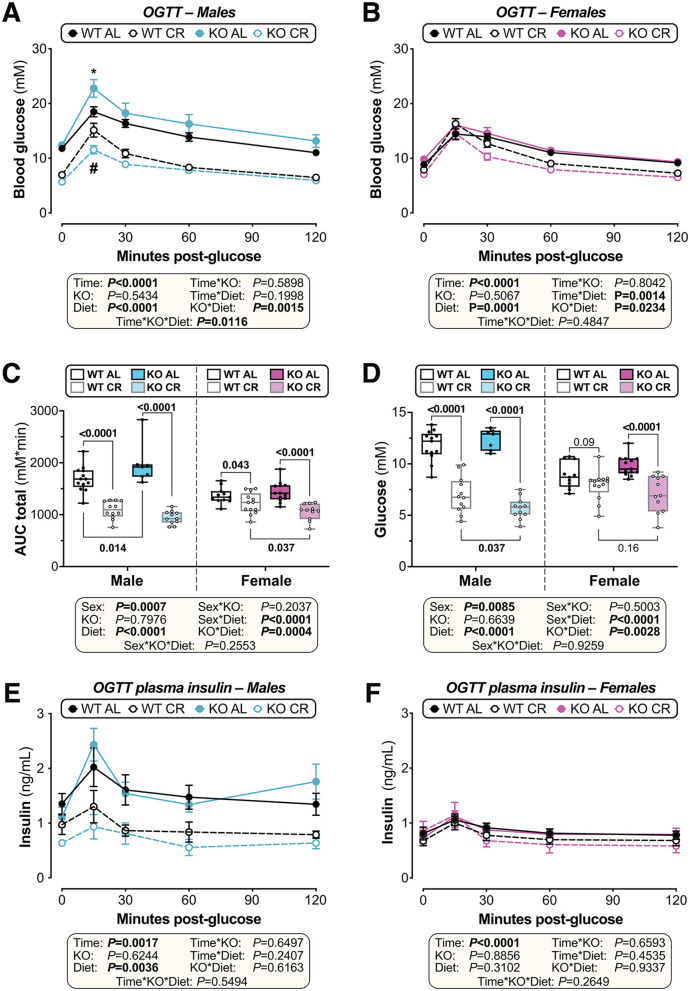
CR-induced decreases in blood glucose are enhanced by adiponectin KO in both sexes. Male and female WT and *Adipoq* KO mice were fed AL or CR as described for Fig 1. At 12.5 weeks of age (3.5 weeks of AL or CR diet) mice underwent an oral glucose tolerance test (OGTT). **(A–B)** Blood glucose readings during the OGTT for male (A) and female (B) mice. **(C)** Area under the curve (AUC) during the OGTT was determined relative to 0 mmol/L (total AUC: tAUC) for each mouse. **(D)** Fasting blood glucose concentrations at time 0 of the OGTT. **(E–F)** Plasma insulin concentrations in males (E) and females (F) during OGTT were assessed using an insulin ELISA. Data are shown as mean ± SEM (A–B, E–F) or as box-and-whisker plots (C–D) of the following numbers of mice per group: *male WT AL*, *n* = 13 (A,C–D) or 11 (E); *male WT CR*, *n* = 12 (A,C–D) or 14 (E); *male KO AL*, *n* = 7 (A,C–D) or 4 (E); *male KO CR*, *n* = 11 (A,C–D) or 4 (E); *female WT AL*, *n* = 11 (B,C–D) or 10 (F); *female WT CR*, *n* = 13 (B,C–D) or 12 (F); *female KO AL*, *n* = 13 (B,C–D) or 10 (F); *female KO CR*, *n* = 11 (B,C–D) or 7 (F). For (A–B) and (E–F), significant effects of diet, time, sex, and/or genotype, and interactions thereof, were determined by 3-way ANOVA (A–B) or mixed-effects models (E–F). For (A–B) Šidák’s multiple comparisons test was further used to assess genotype effects at 0 and 15 min; significant differences between WT and KO mice *(P* < 0.05) are indicated by * or # for AL or CR diets, respectively. For (C–D), statistical analyses were as described for Fig 1E. The underlying data for this figure can be found in the S1 Data file.

Both CR and adiponectin increase glucose tolerance and insulin sensitivity [2,5]. Therefore, we conducted oral glucose tolerance tests (OGTT) to address the hypothesis that adiponectin contributes to these CR effects. In WT mice, CR significantly lowered blood glucose levels in males and, less strongly, in females (Fig 2A–2C) consistent with previously reported sex differences [5]. However, the effects of adiponectin KO were at odds with our expectations: in males, KO *accentuated* CR-induced suppression of blood glucose across the OGTT time course, with 15-min glucose lower in KO than WT males during CR (Fig 2A). A similar KO-Diet interaction also occurred in females, despite the CR effect being smaller than in males (Fig 2B). Across both sexes, adiponectin KO enhanced CR-induced decreases in fasting glucose and the blood glucose total area under the curve (tAUC) during OGTT, suggesting impaired gluconeogenesis and/or increased glucose utilization in adiponectin KO under CR (Fig 2C and 2D). This unexpected effect occurred without discernible alteration of either insulin concentrations (Fig 2E and 2F) or insulin sensitivity, as assessed by the Homeostatic Model Assessment for Insulin Resistance (HOMA-IR) and insulin tolerance tests (S2A–S2C Fig).

### 2.4. Adiponectin KO impairs lipid clearance and utilization in males

Adiponectin and CR each stimulate fatty acid β-oxidation [2,3,11–14]. Therefore, we next hypothesized that adiponectin contributes to increased fat oxidation during CR; that this is compromised by adiponectin KO; and that, via the Randle effect [15], this shifts substrate oxidation towards carbohydrate utilization, causing fasting hypoglycemia in CR KO mice. To test this, we assessed the impact of adiponectin KO on lipid homeostasis.

**Fig 3:**
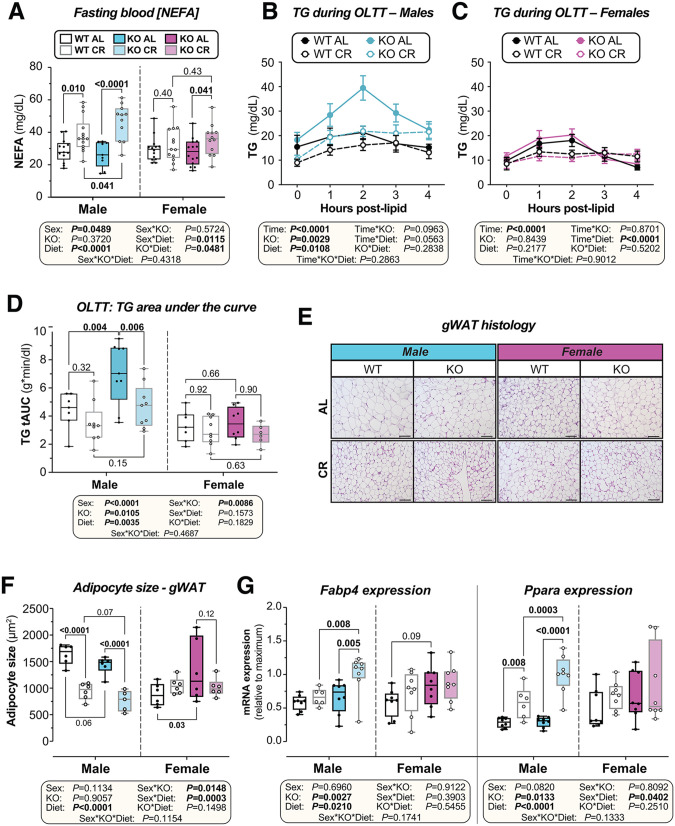
Effects of adiponectin KO on plasma NEFA, lipid tolerance, and white adipose tissue. Male and female WT and *Adipoq* KO mice were fed AL or CR as described for Fig 1. **(A)** Plasma NEFA measured on the day of necropsy (13 weeks of age) in mice fasted for ~12 hours. **(B–D)** At 12.5 weeks of age (3.5 weeks of AL or CR diet) mice underwent an oral lipid tolerance test (OLTT). (B–C) Plasma triglyceride (TG) concentrations for males (B) and female (C) during the OLTT. (D) Total AUC for TG during the OLTT was determined relative to 0 mg/dL TG. **(E–G)** At 13 weeks of age, mice were culled and gWAT was collected. Micrographs of H&E-stained sections of gWAT (E) were used for histomorphometric analysis of adipocyte area (F); in (E), scale bar = 100 µm. **(G)** Expression of *Fabp4* and *Ppara* in gWAT was determined by qPCR and was normalized to expression of the housekeeping genes *Ppia*, *Tbp*, and *Hprt.* For each transcript, expression is shown relative to levels in the sex-diet subgroup with the highest average expression. Data are shown as box-and-whisker plots (A, D, F, G) or as mean ± SEM (B–C) of the following numbers of mice per group: *male WT AL*, *n* = 13 (A), 7 (B, D), 6 (F) or 8 (G); *male WT CR*, *n* = 14 (A), 9 (B, D) or 6 (F–G); *male KO AL*, *n* = 9 (A, B, D), 6 (F) or 8 (G); *male KO CR*, *n* = 11 (A), 9 (B, D), 6 (F) or 8 (G); *female WT AL*, *n* = 14 (A), 7 (C–D), 6 (F) or 8 (G); *female WT CR*, *n* = 13 (A), 9 (C–D), 6 (F) or 8 (G); *female KO AL*, *n* = 14 (A), 9 (C–D), 6 (F) or 8 (G); *female KO CR*, *n* = 12 (A), 6 (C–D, F) or 8 (G). Significant effects of diet, genotype, time, and/or sex, and interactions thereof, were determined by 3-way ANOVA (A–B, D, F–G) or mixed-effects models (C). For (C–D) and (F–G), significant diet effects (within each sex and genotype) or genotype effects (within each sex and diet) were assessed as described for Fig 1E. The underlying data for this figure can be found in the S1 Data file.

Plasma non-esterified fatty acid (NEFA) concentrations increased with CR in WT males but not WT females; adiponectin KO enhanced this effect in both sexes, with CR-induced increases in plasma NEFA being greater in KO versus WT mice (Fig 3A). Thus, adiponectin KO may compromise fatty acid oxidation during CR.

Adiponectin KO impairs lipid tolerance in aging and other contexts [16], but whether this occurs in CR is unknown. Therefore, we tested this using oral lipid tolerance tests (OLTT). In males, plasma TG was decreased by CR but increased by adiponectin KO, indicating that KO impairs lipid clearance; this KO effect was stronger in AL versus CR males (Fig 3B). In contrast, CR in females prevented the increases in TG that occurred during the OLTT, but adiponectin KO had no effect on lipid clearance (Fig 3C and 3D). The effects of adiponectin KO on lipid tolerance therefore differ strikingly between the sexes.

### 2.5. Adiponectin KO decreases adipocyte size in males and has sex-specific effects on transcript and protein expression in gWAT

The KO effects on plasma NEFA and lipid tolerance might result from KO increasing lipolysis, increasing FA synthesis, decreasing FA and lipoprotein uptake, and/or decreasing FA oxidation in WAT. To test this, we analyzed WAT for histological and molecular markers of these processes. As reported previously, CR decreased adipocyte size in males but not in females (Figs 3E, 3F, S3A, and S3B). Adiponectin KO did not influence the CR response but had sex-dependent effects, increasing adipocyte size in AL females but decreasing this in AL males, particularly in iWAT (Figs 3E, 3F, S3A, and S3B). KO AL males also had greater phosphorylation of hormone-sensitive lipase (HSL) and higher perilipin A expression than their WT counterparts (S4A and S4B Fig). In contrast, during CR KO did not affect adipocyte size or HSL activation and prevented the CR-induced upregulation of Perilipin A (Figs 3E, 3F, S3, and S4).

These observations suggest that adiponectin KO increases lipolysis in AL males but not in CR mice of either sex. However, we found that KO influenced expression of several regulators of adipose lipid metabolism. Across all sex-diet subgroups, fatty acid-binding protein 4 (*Fabp4*) and peroxisome-proliferator activated receptor-α (*Ppara*) were increased by adiponectin KO, particularly in CR males (Fig 3G). KO also subtly increased expression of carnitine palmitoyltransferase 1A (*Cpt1a*), a transcriptional target of PPARαinvolved in fatty acid oxidation, and influenced expression of *Pparg2*, *Cebpa*, and *Dlk1*, three transcriptional regulators of adipogenesis (S5A and S5B Fig). Expression of lipoprotein lipase (*Lpl*), fatty acid synthase (*Fasn*), and diacylglycerol O-acyltransferase 2 (*Dgat2*), three critical regulators of lipid uptake and storage, was increased by CR but unaffected by adiponectin KO (S5B Fig). Finally, expression of CD36, the fatty acid transporter, was lower in KO versus WT females during CR and tended to be downregulated by CR in KO but not WT males (S4A and S4B Fig). These effects on CD36 may contribute to increased plasma NEFA in KO versus WT mice during CR; however, the overall KO effects suggest that altered WAT function is not a primary mediator of increased plasma NEFA or impaired lipid clearance in KO mice.

### 2.6. During CR, adiponectin KO enhances postprandial carbohydrate utilization in males and impairs lipid oxidation in fasting females

**Fig 4:**
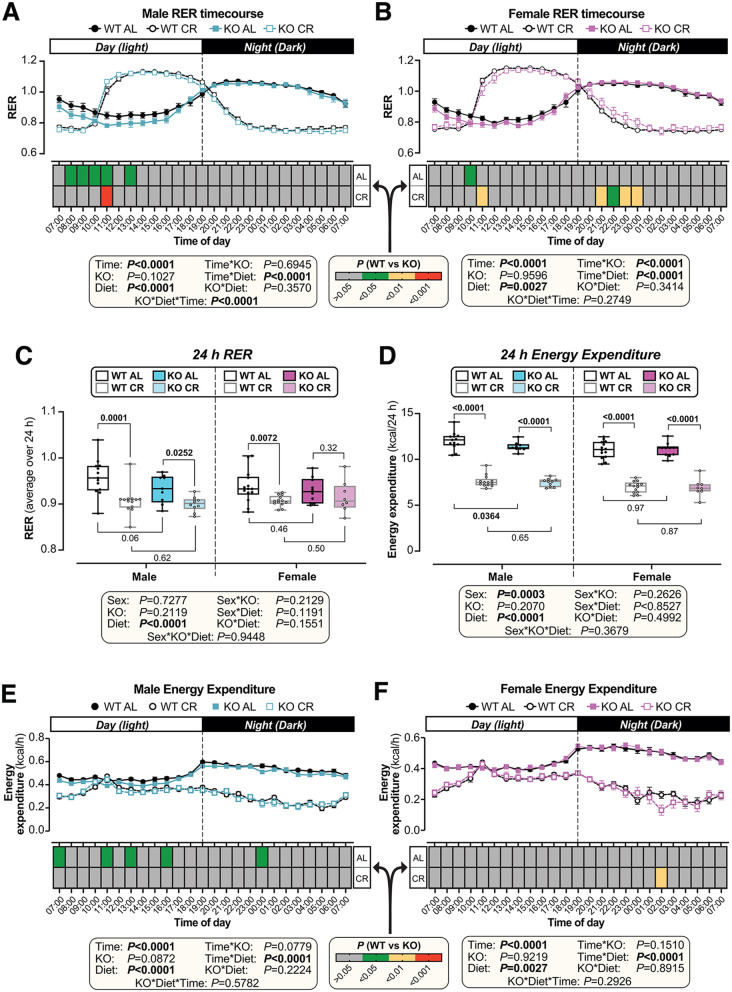
Effects of adiponectin KO and CR on RER and energy expenditure. Male and female WT and *Adipoq* KO mice were fed AL or CR as described for Fig 1. At 11.5 weeks of age (during the third week of AL or CR diet) mice were housed for 4 days in Promethion CORE System cages for indirect calorimetry. Respiratory exchange ratio (RER) and energy expenditure (EE) were recorded every minute throughout the 4 days. **(A)** Male and **(B)** Female RER per hour over the 24-hour light (Day) and dark (Night) periods, based on the average for days 2–4 of Promethion housing. **(C)** Average RER across the 24-hour light-dark cycle. **(D)** Average EE across the 24-hour light-dark cycle. **(E, F)** Male (E) and Female (F) energy expenditure per hour over the 24-hour day and night periods. Data are presented as mean ± SEM (A, B, E, F) or box-and-whisker plots (C, D) of the following numbers of mice per group: *male WT AL*, *n* = 13; *male WT CR*, *n* = 13; *male KO AL*, *n* = 9; *male KO CR*, *n* = 10; *female WT AL*, *n* = 14; *female WT CR*, *n* = 13; *female KO AL*, *n* = 10; *female KO CR*, *n* = 8. Significant effects of genotype, diet, and time or sex were determined by 3-way ANOVA. Overall *P* values from 3-way ANOVA are shown beneath each graph. For (C–D), significant genotype effects (within each sex and diet) or diet effects (within each sex and genotype) were assessed as described for Fig 1E. *P* values from these multiple comparisons are indicated by the color coding for each time point (A, B, E, and F) or shown on the graphs (C–D). The underlying data for this figure can be found in the S1 Data file.

We next investigated if KO alters glucose and lipid homeostasis through effects on systemic substrate utilization and energy expenditure (EE). To do so, we used indirect calorimetry to assess mice during week 3 of CR (Fig 4). Consistent with previous observations [5], feeding CR mice at ~1,030 increased the respiratory exchange ratio (RER) to >1 until the beginning of the dark period, indicating postprandial fatty acid synthesis from dietary carbohydrates [17] (Fig 4A and 4B). RER of CR mice reached a nadir of ~0.7 from 0100 to 0900, a fasting period wherein fatty acids are the predominant fuel source. In contrast, AL mice had greater RER during the dark period and lower RER in the light period, corresponding to periods of increased and decreased feeding, respectively (Fig 4A and 4B). Across the full 24 h light-dark cycle, CR significantly decreased average RER in males and females (Fig 4C). CR also robustly suppressed 24 h energy expenditure (Fig 4D), driven largely by decreased energy expenditure during the night (Fig 4E and 4F).

In AL or CR mice of either sex, adiponectin KO did not alter average RER or energy expenditure over the 24 h period (Fig 4C and 4D) and had relatively little effect on energy expenditure at specific time points (Fig 4E and 4F). However, KO had diet- and time-dependent effects on RER that might reflect its effects on glucose and lipid metabolism (Figs 2 and 3). For example, KO decreased fasting (daytime) RER in AL males and females but increased fasting (nighttime) RER in CR females (Fig 4A and 4B). Moreover, during CR, adiponectin KO enhanced the postprandial increase in RER (10:00–11:00) in males but suppressed this in females (Fig 4A and 4B). These observations demonstrate that although KO does not affect overall RER or EE in AL or CR mice of either sex, it can alter the shifts between lipid and carbohydrate utilization that occur during transitions between fed and fasted states.

Taken together, the results of our tolerance tests and indirect calorimetry suggest that adiponectin plays a role in defending against hypoglycemia during CR by suppressing utilization of glucose as a substrate for energy production.

### 2.7. Adiponectin KO has no effect on liver mass, lipid accumulation, or sphingolipid concentrations in AL or CR-fed mice

The liver plays a pivotal role in orchestrating fluxes in macronutrient metabolism during nutritional transitions and is also a key target of adiponectin [2,18,19]. Thus, we next investigated the hepatic effects of adiponectin KO during both AL feeding and CR.

CR decreased liver mass in males but not females and adiponectin KO did not influence this, regardless of sex or diet (S6A Fig). Analysis across both sexes and genotypes revealed that CR decreased hepatic triacylglycerol (TG) concentrations and lipid droplet area; however, these were unaffected by adiponectin KO in males or females on either diet (S6B–S6D Fig).

We also analyzed hepatic sphingolipids, which can be decreased by adiponectin [20–23] and modulated by CR, especially in males [5]. Targeted lipidomics of male livers showed that CR altered hepatic ceramides, dihydroceramides (DHC), and the ceramide:DHC ratio in a manner dependent on sphingolipid chain length (S1–S3 Tables). However, adiponectin KO did not alter ceramides, DHCs, or the ceramide: DHC ratio in AL or CR mice, nor did it alter their modulation by CR (S1–S3 Tables).

### 2.8. Adiponectin KO has a major effect on lipid metabolism-related genes under AL and on amino acid catabolism-related genes under CR

**Fig 5:**
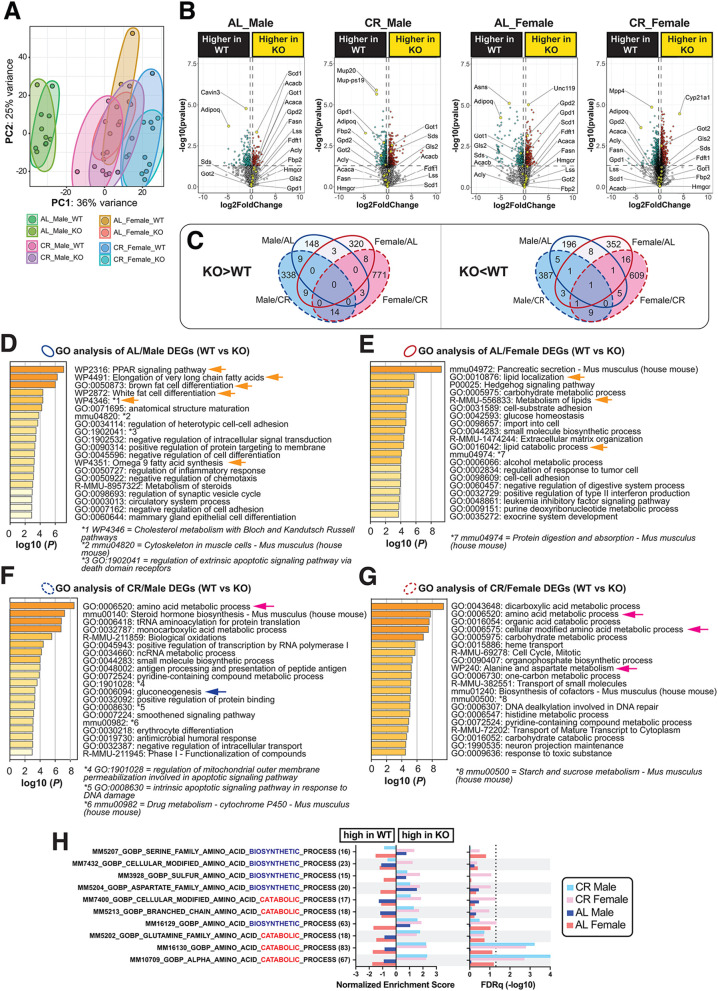
Adiponectin KO enhances hepatic expression of amino acid catabolism genes during CR in both sexes. Male and female WT and *Adipoq* KO mice were fed AL or CR as described for Fig 1. At 13 weeks of age, mice were culled, and livers were collected and analyzed by bulk RNA-seq. The following numbers of mice were used for each group: *male WT AL*, *n* = 6; *male WT CR*, *n* = 5; *male KO AL*, *n* = 5; *male KO CR*, *n* = 5; *female WT AL*, *n* = 5; *female WT CR*, *n* = 6; *female KO AL*, *n* = 6; *female KO CR*, *n* = 6. **(A)** Principal component analysis (PCA) for all 8 groups. **(B)** Volcano plots showing differently expressed genes between WT and KO in each subgroup (AL Male, CR Male, AL Female, and CR Female). Genes with rawp value < 0.05 and absolute fold change >1.2 are shown in blue or red dots. The names of some genes of interest are shown. **(C)** Overlap of genes differentially expressed between WT and KO in each subgroup (AL Male, CR Male, AL Female, and CR Female). Genes increased in KO vs. WT (rawp < 0.05, fold change > 1.2) are shown in the left Venn diagram and genes decreased in KO vs. WT (rawp < 0.05, fold change < −1.2) are shown in the right Venn diagram. **(D–G)** Results of GO analyses conducted with genes differentially expressed between WT vs. KO mice for AL males (380 DEGs), AL females (723 DEGs), CR males (777 DEGs), and CR females (1,438 DEGs); labels above each graph indicate the corresponding sections of the Venn diagrams in (C), The top 20 clusters of GO results are shown for each sex-diet subgroup. Yellow arrows are used to highlight GO terms related to lipid metabolism, blue arrows for gluconeogenesis, and pink arrows for amino acid metabolism. **(H)** GSEA results for gene sets related to amino acid catabolism and amino acid biosynthesis in each subgroup (AL Male, AL Female, CR Male, CR Female), with comparison of WT vs. KO mice. Data were extracted from GSEA results with the M5 gene set library (m5.all.v2023.2.Mm.symbols.gmt). Gene sets are arranged according to the normalized enrichment score (NES) value in CR Male. The left graph shows NESs and the right graph shows −log10 (FDRq) values. For male CR, gene set MM10709 returned an FDRq result of 0; for visualization purposes, this is shown with a −log10 (FDRq) value of 4 in the graph. The dashed line indicates FDRq of 0.05. Some genes are not presented in the volcano plots in (B) because of their high raw *p* values or high absolute FC values; these genes are summarized in S4 Table. The underlying data for this figure can be found in the S1 Data file.

To interrogate the hepatic effects of adiponectin KO in more detail, we analyzed livers by bulk RNA-seq. Principal component analysis (PCA) demonstrated that sex and diet caused many more transcriptional changes than adiponectin deficiency, with WT and KO samples colocalising within each sex-diet subgroup (Fig 5A). Nevertheless, between WT and KO mice we identified 380 differentially expressed genes in AL males, 777 in CR males, 723 in AL females, and 1,438 in CR females (Fig 5B and 5C and S4 Table). KO-affected genes showed minimal overlap across subgroups (Fig 5C), indicating that adiponectin’s impacts on hepatic gene expression are highly dependent on sex and nutritional state.

Gene ontology (GO) analysis of the genes differentially expressed between KO and WT revealed that, in AL males and females, adiponectin KO altered transcripts relating to lipid metabolism (Fig 5D and 5E, yellow arrows). Gene set enrichment analysis (GSEA) further revealed that sterol biosynthesis-related genes were upregulated in KO under AL in both sexes (S7A and S7B Fig). Changes in FA biosynthesis-related genes were also highly enriched in KO in both sexes under AL, and in females but not males under CR (S7C and S7D Fig). In contrast, during CR, amino acid metabolism-related genes were among the most significantly enriched in KO versus WT mice of both sexes (Fig 5D and 5E; pink arrows). GSEA further revealed that, among these genes, transcripts related to amino acid catabolism were particularly enriched in KO versus WT in both sexes (Figs 5G, S8A, and S8B). Indeed, 35 genes in CR males and 39 genes in CR females showed core enrichment for the MM10709_GOBP_ALPHA_AMINO_ACID_CATABOLIC_PROCESS gene set, among which 19 genes were common between the sexes (S8C and S8D Fig).

### 2.9. Adiponectin KO does not alter skeletal muscle mass or myofibre size

SkM is a major source of gluconeogenic amino acids during fasting [24]. Thus, we speculated that, if adiponectin KO increases hepatic amino acid catabolism, this is fueled by amino acids from SkM. To test this, we analyzed the masses of gastrocnemius and soleus muscles and myofibre cross-sectional area (CSA) of gastrocnemius muscle. Overall, muscle mass was lower in females versus males and was significantly decreased by CR (S9A–S9D Fig), which also lowered gastrocnemius myofibre CSA (S10A and S10B Fig). However, adiponectin KO did not affect these parameters, nor did it alter their modulation by CR (S9 and S10 Figs). Thus, adiponectin is unlikely to substantially influence SkM protein breakdown and amino acid release to the circulation under CR.

### 2.10. Adiponectin KO does not alter hepatic or plasma amino acid concentrations

To further assess the impact of adiponectin KO on amino acid metabolism, we used liquid chromatography tandem mass spectrometry (LC–MS/MS) to measure amino acid concentrations in the liver and plasma. Of 20 species, only Asp, Gln, and Tyr were undetectable. In the liver, both diet effects and sex differences were relatively modest: CR increased glycine and glutamate concentrations and the latter were higher in females than in males, but no other diet or sex effects occurred (S5 Table). Under AL feeding, glycine levels tended to be lower in adiponectin KO livers compared to WT controls, whereas under CR, KO livers tended to exhibit higher glycine levels (S5 Table). This suggests that adiponectin KO may suppress glycine catabolism under CR.

CR exerted more widespread effects on plasma amino acids, 12 of which were decreased but none increased in CR versus AL mice (S6 Table). In addition, sex differences were detected for 13 amino acids, with males in each case exhibiting higher concentrations than females. However, adiponectin KO did not influence plasma amino acid concentrations or their modulation by CR (S6 Table).

Adiponectin KO increases plasma branched-chain amino acids (BCAAs) in obese mice [25]. Therefore, we also calculated the absolute amounts of BCAAs in liver and plasma, as well as the proportion of BCAAs relative to total amino acids (excluding Asp, Gln, and Tyr). BCAAs in plasma, but not liver, were higher in males and decreased by CR in both sexes; however, adiponectin KO did not affect hepatic or plasma BCAAs, regardless of sex or diet (S5 and S6 Tables). These data suggest that adiponectin-mediated regulation of amino acid catabolism-related genes in the liver under CR does not exert a major impact on hepatic or systemic amino acid metabolism.

### 2.11. Adiponectin KO does not alter gluconeogenesis from glycerol during CR

Our RNA-seq data also showed that, in CR males, KO altered hepatic expression of gluconeogenesis-related genes, including highly expressed genes such as *Got1*, *Sds*, and *Gpd1*, and genes with lower expression, such as *Gpd2* and *Fbp2* (Figs 5B, 5F [gene set GO0006094, blue arrow], and S11A– S11E). In contrast, no gluconeogenesis-related gene sets were among the top GO results in CR females (Fig 5G). The lower expressions of *Gpd1* and *Gpd2* in KO versus WT males during CR (Figs 5B, 5F, S11A, and S11B) suggest that KO may compromise gluconeogenesis from glycerol. This could explain the lower fasting blood glucose levels in KO males under CR (Fig 2D), as glycerol is the main gluconeogenic substrate in fasting mice [26]. However, glycerol tolerance tests revealed that adiponectin KO moderately impaired gluconeogenesis in AL males only, with no KO effects in CR in either sex (S11F and S11G Fig).

Together, our liver RNA-seq data demonstrate a clear contrast in the roles of adiponectin under AL and CR: in both male and female livers, adiponectin primarily targets lipid biosynthesis-related genes during AL feeding but amino acid catabolism-related genes during CR. These hepatic effects provide further clues about the mechanisms through which adiponectin KO may impact systemic glucose and lipid metabolism and highlight that adiponectin’s functions are highly diet- and sex-specific.

## 3. Discussion

**Fig 6:**
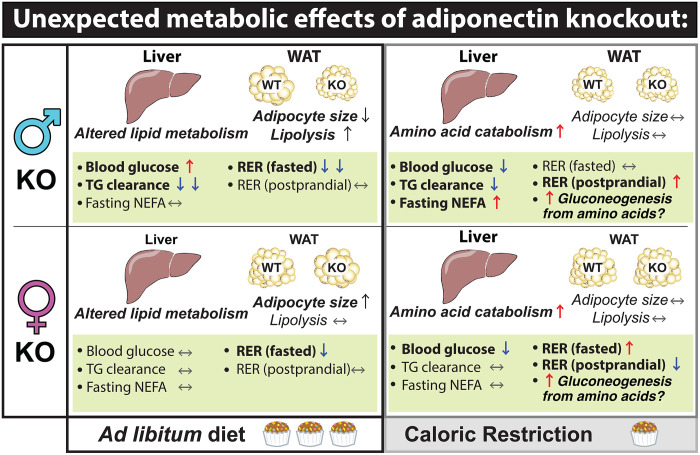
Adiponectin has distinct effects on lipid, amino acid, and glucose metabolism under AL and CR. Under AL diet, adiponectin KO alters hepatic lipid metabolism in both sexes, including effects on FA and sterol biosynthesis. Adiponectin KO also decreases RER during fasting (daytime) in AL males and females, suggesting increased lipid vs. carbohydrate utilization. However, some KO effects are sexually dimorphic: in AL males, KO causes mild hyperglycemia, impaired TG clearance, increased adipocyte lipolysis and adipocyte hypotrophy, but these KO effects are absent in AL females, in which KO increases adipocyte size. Under CR, adiponectin KO enhances hepatic amino acid catabolism through the regulation of gene expression. In contrast to AL, adiponectin KO promotes mild hypoglycemia and increased fasting NEFA under CR, and this may be driven, in part, by differential effects on RER in the fasted and postprandial states. Liver, WAT, and cake illustrations were drawn in Illustrator by William Cawthorn.

Adiponectin has been studied extensively in obesity and insulin-resistant states, in which circulating adiponectin decreases. Yet adiponectin’s fundamental roles in resilience to evolutionarily relevant stressors, such as sustained negative energy balance, have been largely overlooked. Several studies report that CR in animal models or humans increases glucose tolerance and insulin sensitivity without increasing circulating adiponectin [27–31], suggesting that elevated adiponectin is not required for these metabolic benefits. We have now directly tested this by investigating the impact of adiponectin KO on the metabolic effects of CR. We show that adiponectin KO does not influence CR-induced weight loss, changes in body composition, suppression of energy expenditure, or decreases in average 24 h RER. However, unlike in obesity, during CR adiponectin KO unexpectedly decreases blood glucose during fasting and following an oral glucose load. Adiponectin KO also augments CR-induced increases in plasma fatty acids in both sexes, without altering markers of lipolysis in WAT, and significantly reduces lipid clearance in males. These KO effects on systemic glucose and lipid metabolism are supported by our indirect calorimetry data, which suggest that adiponectin KO alters the shifts between carbohydrate and lipid utilization that occur during transitions between fed and fasted states. Finally, our bulk liver transcriptomic analyses identify diet-dependent effects: in both sexes, adiponectin KO predominantly alters hepatic amino acid catabolism-related gene expression during CR, but expression of lipid metabolism-related genes under AL diet. Together, our findings reveal new roles of adiponectin in the adaptive response to CR and highlight sex and diet as key determinants of adiponectin’s systemic and tissue-specific metabolic effects (Fig 6).

Previous studies have used different lines of adiponectin KO mice that often have different phenotypes. A recent preprint revealed that two previous KO lines maintain some expression of exon 3, which encodes the functional globular domain, and that this influences the phenotype compared to KO mice that lack exon 3 [32]. Thus, an important aspect of our study is that we used a new adiponectin KO model that completely lacks exon 3, distinguishing it from prior partial KOs.

Among the metabolic effects, it is particularly striking that adiponectin KO lowers blood glucose levels in OGTT during CR, because adiponectin is typically regarded as an insulin-sensitizing hormone that improves glucose tolerance and prevents hyperglycemia. However, on a normal, non-obesogenic diet, adiponectin KO mice have unaltered glucose tolerance, whether they are young, aged, or insulin-deficient [13,16,33–35]. Moreover, one human study found that CR *decreases* high-molecular-weight adiponectin and that this is associated with increased glucose tolerance [31]. Thus, the impact of adiponectin deficiency on glucose homeostasis appears critically dependent on dietary context.

We show that, during CR, adiponectin KO lowers blood glucose without augmenting insulin concentrations or insulin sensitivity, or impairing gluconeogenesis from glycerol. Instead, KO alters the changes in RER during transitions between fed and fasted states. In CR males, the rapid postprandial increase in RER is augmented in KO versus WT mice. This suggests that, during CR, adiponectin KO enhances postprandial glucose utilization in males, an effect that likely also occurs after oral glucose administration. This may explain why, in CR males, KO decreases fasting and peak glucose during the OGTT. In contrast, in CR females, adiponectin KO *attenuates* the postprandial rise in RER, which may explain why KO does not suppress OGTT peak glucose in CR females. Instead, KO suppresses the decline in RER as CR females progress from the fed (daytime) to fasted (nighttime) state. This suggests that, under CR, KO females resist the increase in lipid oxidation and/or sustain elevated carbohydrate oxidation during the onset of fasting, indicating impaired metabolic flexibility. These effects may underlie the lower OGTT blood glucose and increased fasting NEFA observed in KO versus WT females during CR.

It remains less clear how KO increases fasting NEFA during CR. Our WAT analyses show that CR increases expression of several regulators of FA metabolism, including *Pparg2*, *Ppara*, *Fabp4*, *Fasn*, and *Dgat2*, which is consistent with previous CR studies [36]. Adiponectin KO also decreases adipocyte size and increases HSL activation and perilipin A expression in AL-fed males, consistent with adiponectin’s anti-lipolytic effects [37]. During CR, we show that KO significantly decreases WAT CD36 expression in females and tends to do so in males, which might contribute to increased fasting NEFA in KO mice [38]. However, adiponectin KO does not alter adiposity, CR-induced fat loss, adipocyte size, or HSL activation during CR; thus, increased WAT lipolysis is unlikely to play a role.

We report several other effects of adiponectin KO on the expression of lipid-metabolism-related genes and proteins in WAT, but these also are unlikely to mediate the changes in plasma NEFA and lipid clearance. For example, increased *Fabp4*, *Ppara*, and *Cpt1a* expression in KO WAT might be expected to decrease plasma NEFA, because *Fabp4* deficiency increases plasma NEFA during fasting [39] and PPARα and CPT1a promote adipocyte FA oxidation [40]. A limitation is that transcript and protein expression alone do not always reflect metabolic activity. Thus, our data show that adiponectin KO exerts diet- and sex-dependent effects on WAT, but the systemic relevance of these remains to be determined. Future studies using labeled FA tracers would be helpful to determine how adiponectin KO impacts systemic and tissue-specific FA metabolism during CR.

In the liver, we show that adiponectin KO enhances expression of genes involved in amino acid catabolism during CR in both sexes, underscoring the robustness of this effect. However, KO does not alter hepatic or plasma amino acid concentrations. This is in contrast to humans, in which plasma adiponectin associates inversely with circulating amino acid levels [41]. This could reflect species differences in the relationship between adiponectin and plasma amino acids; however, another possibility is that, in CR-fed KO mice, increased hepatic amino acid catabolism leads to compensatory mechanisms that prevent changes in net amino acid concentrations. If so, studies using labeled amino acid tracers may be required to uncover the full impact of adiponectin KO on amino acid metabolism.

This knowledge could help to elucidate how adiponectin KO influences systemic glucose homeostasis during CR. Indeed, our transcriptomic data suggests that KO males and females have increased reliance on amino acid catabolism to support gluconeogenesis, exemplified by increased expression of *Got1* and *Sds* in KO versus WT males during CR. These KO effects are notable because gluconeogenesis from amino acids is less metabolically efficient than from glycerol [42]. Moreover, when lipid utilization is constrained, amino acid metabolism becomes rate-limiting for gluconeogenesis, resulting in fasting hypoglycemia [43]. We speculate that these hepatic effects also contribute to KO-induced hypoglycemia and lower OGTT glucose during CR. Importantly, the RER for amino acid oxidation is ~0.8 whereas for lipids it is 0.7; thus, if KO does cause a shift from lipid or glucose to protein oxidation during CR, this might not be detectable through systemic RER measurements. Further investigation will be required to determine the mechanism by which KO mice exhibit hypoglycemia under CR.

The basis for adiponectin KO upregulating amino acid catabolism genes also remains unclear. PPARα is critical for the hepatic response to fasting, regulates both lipid and amino acid metabolism [44–49], and can be activated by adiponectin. Consistent with this, there is extensive overlap between PPARα target genes related to amino acid metabolism and the genes enriched in KO livers under CR (S8C and S8D Fig) [50]. Thus, one possibility is that adiponectin acts via PPARα to regulate hepatic amino acid metabolism under CR. Hepatic amino acid metabolism also contributes to ammonia detoxification and the ability to salvage amino acids [24]. Therefore, beyond gluconeogenesis, adiponectin may also have evolved to facilitate ammonia detoxification and support amino acid homeostasis in energy-deficient, catabolic states.

Our findings highlight several other hepatic effects of CR and adiponectin. The adiponectin receptors have ceramidase activity, and many studies report that adiponectin suppresses hepatic ceramide content [20–23]. Yet we show no effect of adiponectin KO on hepatic ceramides, DHCs, and ceramide:DHC ratios in AL or CR mice, whether for total concentrations or for the individual species. This echoes previous studies finding limited or no effect of adiponectin on ceramides in SkM or the pancreas [13,35]. Therefore, adiponectin’s hepatic ceramide-lowering actions may be limited to states of obesity, insulin resistance, and/or hyperinsulinaemia.

Another insight relates to the roles of adiponectin in normal dietary contexts, beyond obesity or CR. Given that KO alters lipid metabolism genes in AL-fed mice, it is surprising that adiponectin KO does not affect their hepatic TG content; however, this is consistent with earlier reports showing that adiponectin KO does not alter hepatic TGs in chow- or HFD-fed males [33] or affect liver TG secretion [51]. Liu and colleagues demonstrated that, in chow-fed male mice, adiponectin KO alters expression of lipid metabolism genes and downregulates genes linked to liver detoxification, but does not influence genes relating to mitochondrial function or beta oxidation [52]. Our RNAseq data confirm these KO effects in AL males and show that they extend to AL females. However, we further demonstrate that adiponectin KO does not decrease hepatic expression of glycolytic enzymes (*Gck*, *Pdhb*, or *Pfkb1*) or the transcription factors *Ppara*, *Hnf4a*, and *Srebf1.* This differs to the results from Liu *and colleagues* but aligns with those from other adiponectin KO studies [35]. Our results thereby refine previous observations by identifying the hepatic functions most robustly influenced by adiponectin deficiency in normal physiological states.

Despite having no impact on hepatic TG or sphingolipid content, we show that adiponectin KO has diet- and sex-specific effects on systemic lipid metabolism. Notably, KO significantly reduces lipid clearance in males and this effect is stronger on AL than CR diet. This adiponectin KO effect in male mice occurs in many other contexts, including in aged males fed chow or high-fat diet [16], in type 1 diabetic males [35], and in males with tissue-specific adiponectin deletion in WAT or the kidney [53,54]. In many cases, this relates to the ability of adiponectin to stimulate lipoprotein lipase activity [51], which decreases with adiponectin deficiency in mice and humans [54,55]. In adiponectin KO mice, this decrease is associated with reduced *Lpl* expression in WAT [54]. However, we show that KO does not alter *Lpl* expression in WAT. This supports the possibility that impaired lipid clearance in KO males is not a result of decreased LPL activity, echoing findings from type 1 diabetic mouse models [35]. Thus, in our AL and CR mice, and in type 1 diabetic models, impaired lipid clearance in KO males may result from effects on other organs, such as impaired intestinal lipid absorption. No previous studies have assessed lipid tolerance in KO females; however, transgenically increased adiponectin improves lipid clearance in mice of both sexes [16,56]. Together, the present and previous observations suggest that increased lipid clearance is among the most-robust metabolic effects of adiponectin in males, whereas in females this is more context-dependent.

When interpreting our findings, it is important to note that our CR regimen involves administering a single daily ration at 9–10 AM (during the light phase), whereas AL mice consume most of their food during the dark phase. This altered feeding pattern can affect metabolic phenotypes and transcriptomic patterns, including in the liver [57]; hence, one possibility is that the KO effects would differ on a night-fed CR regimen. However, we previously showed that, in WT mice, CR’s effects on adiposity and glucose homeostasis are similar in morning- versus night-fed mice [5]. Moreover, our CR regimen elicits transcriptional effects similar to those in night-fed CR mice, including increasing *Pparg2*, *Ppara*, *Fabp4*, *Fasn*, and *Dgat2* in WAT [36] and upregulating transcriptional signatures of glycolysis, the TCA cycle, beta-oxidation, and gluconeogenesis in the liver [5,58]. Therefore, our observed KO effects during CR are likely to be generalizable to other CR regimens.

While we have focused on the liver as the key target of CR and adiponectin, a recent study highlights roles for adiponectin in renal gluconeogenesis [53]. This is notable because renal gluconeogenesis contributes to ~40% of glucose production in starvation [59] and can compensate for impaired hepatic gluconeogenesis [60,61]. Adiponectin also targets many other cells and tissues, including SkM, beta cells, the heart, endothelia, leukocytes, and adipose tissue. Thus, investigating the kidney and other targets may further resolve the mechanisms underlying adiponectin’s unexpected effects in CR.

Finally, across our experiments, we consistently find that the impact of adiponectin deficiency is diet- and sex-dependent. This suggests that adiponectin’s metabolic actions depend critically on other variables influenced by these factors, including insulin, glucagon, leptin, ghrelin, glucocorticoids, estrogens, and androgens. Indeed, each of these hormones exerts fundamental metabolic effects, including during CR [3], while sex steroids often underpin sex differences in metabolic function [62]. Importantly, most preclinical studies of increased or decreased adiponectin action have been in male mice, overlooking potential sex differences. It will therefore be critical to further establish the extent of, and basis for, adiponectin’s diet-dependent, sexually dimorphic effects.

Together, our study shows that the roles of adiponectin in tissue-specific and systemic metabolism differ significantly under AL and CR, providing insight into the potential evolutionary functions of this enigmatic hormone. Future research should seek to determine the molecular mechanisms by which adiponectin affects lipid, amino acid, and glucose metabolism under CR, and if adiponectin regulates other aspects of the CR response. Given that our CR regimen is relatively mild, it would also be informative to determine the consequences of adiponectin deficiency in the context of starvation or other states of greater energy deficit. Finally, while CR can serve as a paradigm to understand the fundamental roles of adiponectin, it is more widely studied for its potential therapeutic benefits. In this light, it will be important to test if variation in adiponectin influences human responses to CR, time-restricted feeding, intermittent fasting, incretin-based weight loss treatments, or other therapeutic nutritional interventions.

## 4. Materials and methods

Reagents and resources used in this study are described in S7 Table.

### 4.1. Animals

All mouse studies were approved by the University of Edinburgh Animal Welfare and Ethical Review Board and were conducted under project licenses granted by the UK Home Office (license numbers PPL708617 and PP2299608). To generate *Adipoq* KO mice, sperm from transgenic *Adipoq*^tm1a(KOMP)Wtsi^ mice on a C57BL/6N background was purchased from The Knockout Mouse Project (KOMP) Repository (mouse colony TCPA0796, Toronto Centre for Phenogenomics, Toronto, Canada) and used by the Central Transgenic Core facility (University of Edinburgh) for in vitro fertilization (IVF) of eggs from WT C57BL/6NCrl females. During IVF, Tat-Cre recombinase was injected into eggs for recombination at the LoxP sites [63]. This excised *Adipoq* exon 3 at the single-cell stage, converting the *Adipoq*^tm1a(KOMP)Wtsi^ allele to the full KO allele (S1A Fig). *Adipoq*^tm1b(KOMP)Wtsi^ heterozygotes were mated to generate KO and control (WT) mice. Littermate controls were used throughout, except for four separate C57BL/6NCrl males (which were included in the OGTT and ITT studies). Genotyping was done by Transnetyx (Cordova, Tennessee, USA).

Mice were housed on a 12 h light/dark cycle in a specific-pathogen-free facility with free access to water. Mice had free access to food unless they were undergoing CR, as described below. Sample sizes were determined by power calculations using G*Power software, with effect sizes based on previously published data for glucose tolerance and fat loss during CR [64]. Single mice were used as experimental units because mice were single-housed. Comparisons were made between AL versus CR mice (within each genotype), and WT versus KO mice (within each diet). The exact number of mice is stated in each figure legend. Randomization, blinding, and exclusion of mice from final analyses were as described [5], with KO and WT mice randomly assigned to each diet. Seven mice were excluded because of confounding health and/or technical issues, including one KO AL male with esophageal damage from OGTT gavage; four males (two WT AL, two WT CR) with gavage damage during OLTT; and two females (one KO, one WT) with unexplained excessive weight loss during CR. For some cohorts, specific tissues were not weighed at necropsy, and therefore these mice have no data for these readouts; further details will be provided in the Source Data file.

### 4.2. Mouse CR studies

Mice were fed AL (Research Diets D12450B) or CR (Research Diets D10012703, administered at 70% of the average daily AL diet consumption) as described previously, with the daily ration of CR diet provided at 09.00–10.00 each morning [5]. Mice were assessed non-invasively for body fat, lean mass, and free fluid weekly from weeks 0 to 4 of AL or CR feeding (~9–13 weeks of age) using TD-NMR (Minispec LF90II; Bruker Optics, Billerica, MA, USA). Blood glucose was measured from weeks 0 to 4 using a OneTouch Verio Glucometer (LifeScan IP Holdings, Zug, Switzerland). Before necropsy, mice from both AL and CR groups were fasted for ~6–12 h to ensure that any AL versus CR differences were not simply a result of a longer fasting duration in the CR mice; this approach has been recommended by other recent CR studies [65]. Mice were then humanely sacrificed via cervical dislocation and decapitation. Tissues were dissected, weighed, and stored in 10% formalin or on dry ice. Tissues in formalin were fixed at 4 °C before being washed and stored in DPBS (Life Technologies). Tissues on dry ice were stored at −80 °C prior to downstream analysis.

### 4.3. Adiponectin, NEFA, and TG analysis in plasma samples

Plasma concentrations of adiponectin (MRP300, Bio-Techne) or TG (Serum Triglyceride Determination Kit, TR0100, Sigma-Aldrich) were measured by ELISA or colorimetric assays as per manufacturer’s instructions. Plasma NEFA were measured as described [5]. Assay absorbances were measured using an Infinite 200 Pro plate reader (Tecan Life Sciences). Immunoblotting of plasma to confirm the presence or absence of adiponectin in WT and KO mice was done as described [64]. Total plasma protein was determined using total protein stain (926-11010, LiCor).

### 4.4. Histology and quantification of adipocyte sizes, liver lipid droplet accumulation, and myofibre cross-sectional area (CSA)

For WAT and liver assessment, formalin-fixed iWAT, gWAT, and livers were paraffin-embedded and sections were made (5 µm thick for WAT; 3–4 µm thick for liver). The sections were stained with hematoxylin and eosin (H&E). Five images (20× objective) were taken per section using a Keyence BZ-X800 microscope. Adipocyte sizes were quantified using the ImageJ plug-in Adiposoft [66]. Lipid droplet area in liver sections was estimated by using the Keyence BZ-X800 Analyzer (Hybrid cell count: BZ-H4C and Macro cell count: BZ-H4CM) to measure the area of white vacuoles within the tissue; note that some sections showed a whitening of cytoplasm in hepatocytes, possibly because of cell damage or glycogen deposition, which may over-estimate lipid droplet area.

For muscle analysis, formalin-fixed gastrocnemius muscles were paraffin-embedded and sections (4 µm thick) were made at the widest lateral point of the muscle, de-waxed, stained with WGA-Alexa Fluor 488 (1:200) for 1 h, washed with PBS, stained with DAPI (1:100), and mounted with PermaFluor mounting medium. Three-to-seven images (10× objective) were taken per section using a Nikon Eclipse microscope and myofibre CSA analyzed using ImageJ plug-in StarDist and MorphoJ. Particles >250 µm^2^ were used for calculation, with at least 689 fibers assessed per section.

### 4.5. Protein isolation and western blotting

Protein isolation from frozen gWAT samples, quantification of protein concentrations, SDS polyacrylamide gel electrophoresis, transfer to Immobilon-FL membranes (Millipore, MA, USA), incubation in primary and secondary antibodies, and detection of fluorescent signal from antibodies was done as described previously [5], except that 24 µg of protein was loaded per lane on Criterion TGX gels (Bio-Rad, Hercules, CA, USA). Details of primary and secondary antibodies are in S7 Table. Coomassie-stained membranes were visualized with a Licor Odyssey M using the RGB channel and quantified using ImageJ.

### 4.6. RNA isolation, reverse transcription, and qPCR

RNA isolation, reverse transcription, primer design/validation, and qPCR were done as described previously [67,68]. For each transcript, mRNA levels were normalized to the geometric mean of the expression of three housekeeping genes (*Hprt1*, *Ppia*, *Tbp*) and expressed relative to whichever sex/diet/KO subgroup has the highest expression. Taqman assays (Thermo Fisher) were used to analyze *Dgat2* (cat. no. Mm00499536_m1) and *Fasn* (cat. no. Mm00662319_m1) expression. All other primers are described in Table 1.

**Table 1:** Primers used for Sybr Green qPCR.

Transcript	Forward Primer (5′-3′)	Reverse Primer (5′-3′)
*Adipoq (*exons 1–2)	GACGACACCAAAAGGGCTCA	AGTGCCATCTCTGCCATCAC
*Adipoq* (exon 3)	AAGAAGGACAAGGCCGTTCTCTT	GCTATGGGTAGTTGCAGTCAGTT
*Cebpa*	TGGACAAGAACAGCAACGAG	TCACTGGTCAACTCCAGCAC
*Cpt1a*	CATGATTGCAAAGATCAATCGG	CTTGACATGCGGCCAGTG
*Dlk1* (Pref1)	CCTCCTGTTGCAGTATAACAGCG	GGTCATGTCAATCTTCTCGGG
*Fabp4*	TGGAAGCTTGTCTCCAGTGA	AATCCCCATTTACGCTGATG
*Ppara*	CCTGAACATCGAGTGTCGAATAT	TCTTCTTCTGAATCTTGCAGCT
*Pparg2*	CCAGAGCATGGTGCCTTCGC	TTCCGAAGTTGGTGGGCCAGA
*Hprt1*	TCATTATGCCGAGGATTTGGA	GCACACAGAGGGCCACAAT
*Lpl*	GGGAGTTTGGCTCCAGAGTT	TACATTCCCGTTACCGTCCA
*Ppia*	CACCGTGTTCTTCGACATCA	CAGTGCTCAGAGCTCGAAAGT
*Tbp*	ACCTTATGCTCAGGGCTTGG	GCCGTAAGGCATCATTGGAC

### 4.7. TG quantification in the liver

Approximately 100 mg of frozen liver tissue was placed in 1 mL cooled PBS containing 5% v/v IGEPAL CA-630 (Sigma, I8896) on ice. Tissue was homogenized using steel ball bearings and high-frequency shaking and this homogenate was centrifuged at 12,000 rcf for 10 min at 4 °C to pellet debris. Each supernatant was transferred to a new microtube, and any floating lipid was resuspended. Pre-prepared 500 mg/dL TG standard (Sigma, 17811-1AMP) was serially diluted to 250, 125, 62.5, 31.25, 15.625, and 7.8125 mg/dL; a solution containing no TG standard was included as a negative control. TG concentrations in standards or liver homogenates (2 µL) were determined using an enzymatic colorimetric assay (Sigma TR0100) according to the manufacturer’s instructions. Concentrations were normalized to the mass of liver used for each sample.

### 4.8. Metabolic tolerance tests

Mice were fed a half portion of food the evening (18:00) prior to the test. At 12:00 on the day of the test, basal blood glucose from lateral tail vein blood was measured using a OneTouch Verio glucometer; for OGTT and OLTT, a basal blood sample was also collected into EDTA-coated tubes (Microvette 16.444, Sarstedt, Leicester, UK). For OGTT, D-glucose (25% w/v in water) was then administered at 2 mg/g body mass by oral gavage. For OLTT, Intralipid was administered by gavage at 15 µL/g body mass. For ITT, Humulin (Eli Lilly and Company, Basingstoke, UK) was injected intraperitoneally at 0.7 mlU/g of lean mass (assessed by TD-NMR the day before ITT). For glycerol tolerance tests, glycerol (10% w/v in saline) was injected intraperitoneally at 1.5 mg/g body mass. Glucose, lipid, and glycerol were dosed relative to body mass to ensure that both lean and fat mass were included; this is important because adaptations in adipose tissue contribute to the systemic metabolic effects of CR. However, insulin was dosed relative to lean mass because CR mice are prone to pathological hypoglycemia following insulin administration; we found that dosing insulin relative to lean mass gives a more predictable response, with lower incidence of hypoglycemia, and therefore used this method for our studies. Blood glucose was measured at the following timepoints post-substance administration: 15-, 30-, 60-, and 120-min for OGTT and glycerol tolerance tests; and 15-, 30-, 60-, and 90-min for ITT. For OGTT and OLTT, tail vein blood was sampled at each time point in EDTA tubes and kept on ice until plasma isolation.

Plasma insulin during OGTT was measured using the Ultra-Sensitive Mouse Insulin ELISA Kit (#90,080, ChrystalChem, Chicago, USA,) as per manufacturer’s instructions. HOMA-IR was calculated as described [69].

### 4.9. Indirect calorimetry

Mice were housed individually in Promethion CORE System cages (Sable Systems International) from weeks 2 to 3 after beginning AL or CR feeding. Mice entered cages around 11:00 on day 1 and were housed for three nights (four days in total). Data are from the 48 h between 07:00 on day 2 and 07:00 on day 4 to allow environmental habituation during day 1. Before and after Promethion housing, mice were weighed and body composition determined by TD-NMR. Energy expenditure, O_2_ consumption, and CO_2_ production were analyzed using ExpeData software as per manufacturer’s instructions.

### 4.10. Liver ceramide quantification

Liver ceramide and DHC content was determined using LC–MS (Lipidomics Core Facility; University of the Highlands and the Islands, Inverness, UK) as described previously [5]. In the final analysis, 26:0 ceramide values were excluded from three mice (two AL WT males and one CR KO male) because they were extreme statistical outliers (as identified using the Rout method with *Q* = 1%, based on variance of combined values from across all diet and genotype groups). The ceramide:DHC ratio was also excluded for these mice.

### 4.11. Amino acid profiling in mouse tissue and plasma by LC–MS/MS

Frozen powdered mouse liver tissue was weighed (~20 mg) directly into plastic 2 mL bead tubes containing 2.8 mm ceramic beads. To each tube, 500 µL of methanol/water (90:10 (v/v)) was added. Tissues were homogenized using a Bead Ruptor elite with a cyro cooling unit (Omni International, Georgia, USA) for 3 rounds of 30 s at 5 m/s. For mouse plasma extraction, ~ 15 µL of plasma was added to 500 µL of chilled methanol water (90:10 (v/v)) in a 1.5 mL Eppendorf tube. Tubes were vortexed for 30 s and left to precipitate at room temperature for 20 min.

Analytical calibration standards were prepared by serially diluting an amino acid analytical standard mix (Sigma Aldrich, p/n: AAS18 in methanol:water (90:10 (v/v) from 0.008 to 16 ng.

Tubes containing tissue homogenates or plasma sample extracts were centrifuged at 14,000 rpm for 10 min. Calibration standards and sample supernatants were filtered through a Filter+ plate (Biotage, Uppsala, Sweden), and the extracts collected into a 96-well collection plate for LC–MS/MS analysis.

Targeted analysis of calibration standards and extracts by LC–MS/MS was performed on a 6500+ QTrap (Sciex, UK) coupled to an I-Class UPLC system (Waters, UK). The injection volume was 1 µL. Analytes were separated on a Poroshell zHILIC 50 × 2.1 mm, 1.7 µm chromatography column (Agilent, UK) with a gradient elution of mobile phase A: Water with 20 mM ammonium formate (pH 2.8), and mobile phase B: acetonitrile:water 90:10 (v/v) with 20 mM ammonium formate (pH 2.8). The column was maintained at 30 °C. The chromatographic gradient began at 100% B, held for 0.5 min, rising to 30% A over7.5 min with a hold at 30% B for 0.5 min. The column was then set to re-equilibrate to the starting conditions of 100% B for 2 min with a total run time per sample of 10 min.

The IonDrive turbo V ion spray source on the 6500+ QTrap was operated in positive ion mode. Multiple reaction monitoring (MRM) was used for the detection of the amino acids by selecting a parent and product ion. Positive ion spray voltage was set to 4,500 V, with the source temperature maintained at 600 °C. The method included a curtain gas (nitrogen (N_2_)) at 40 psi, collision gas (N_2_) medium, air ion source gas 1 and air ion source gas 2 of 40 psi and 60 psi, respectively. Parent – product ion mass transition parameters and retention times for each of the amino acids are presented in Table 2 below.

**Table 2:** Retention time and mass spectrometry parameters (Q1 – DP, declustering potential; EP, entrance potential; CE, collision energy; CXP, collision exit potential; V, voltage).

Analyte name	Parent ion *(m*/*z*)	Product ion (*m*/*z*)	DP (V)	EP (V)	CE (V)	CXP (V)	Time (min)
Glycine	76.1	30.0	6	7.6	19	14	3.9
Alanine	90.1	44.0	6	4.5	17	6	3.6
Serine	106.1	60.0	6	10.5	15.5	7	4.1
Proline	116.1	70.0	20	13.5	21	10	3.1
Valine	118.1	55.0	11	13.5	27	8	3.0
Threonine	120.1	103.2	105	14.5	25	7	2.1
Isoleucine	132.1	69.0	8	14.5	23	8	2.3
Leucine	132.1	86.0	8	14.5	13	10	2.4
Aspartic Acid	134.1	74.0	7	14.5	19	10	4.8
Lysine	147.1	84.0	15	13.5	23	10	5.9
Glutamic Acid	147.97	84.0	46	10	23	8	3.9
Methionine	150.2	104.0	6	12	15	12	2.6
Histidine	156.1	110	16	13	19	12	5.3
Phenylalanine	166.1	120.1	11	14	20	12	2.1
Arginine	175.2	70	40	11	27	8	5.7
Tyrosine	182.1	136.1	20	10	20	8	2.9
Cystine	241.2	152	20	14	19	10	5.9

Quantities of each amino acid were calculated per sample, following integration of each chromatographic peak in calibration standards and samples, using MultiQuant software (version 2.1, AB Sciex, UK). The calibration curves were assessed using linear regression with a weighting factor of 1/× and amounts calculated in ng before further data analysis.

### 4.12. RNA sequencing

For liver samples, RNA isolation, quantification, determination of the RNA Integrity Number (RIN), library preparation, and sequencing were done as described [5]; data for livers of WT mice were also reported in this previous publication [5].

Calculations were done using Eddie Mark 3, the University of Edinburgh’s high-performance computing cluster. After trimming by TrimGalore and quality control by FastQC, sequences were aligned to the mouse genome (mm10) with annotation data from the website of University of California Santa Cruz (https://hgdownload.soe.ucsc.edu) using STAR (v2.7.10a) [70]. Mapped reads were counted using featureCounts in Subread package (v1.5.2) [71] and subsequent analyses performed using R, RStudio, GSEA, Cytoscape, and Metascape. Count data were normalized and analyzed using DESeq2 (v1.44.0) to detect differentially expressed genes [72].

PCA was done using the prcomp function for 500 genes, with the highest variation among samples after transforming the raw count data using the vst function from the DESeq2 package. The stats and ggplot2 packages were used for gene clustering and heatmapping. GO analysis was done with Metascape (https://metascape.org/gp/index.html#/main/step1) [73]. Metascape shows bar graph for viewing top non-redundant enrichment clusters, one per cluster. Each result shows Top 20 GO terms with −log 10 (p) > 2.0. For GESA, 1,000 gene-set permutations were used to estimate the null distribution for the data given the sample size, and “gene_set” was used for permutation type. But default setting was used otherwise.

### 4.13. Graphs and statistical analysis

Data are shown as mean ± SEM or as box-and-whisker plots. For the latter, the centre line is the median, the box extends from 25th to 75th percentile, the whiskers denote the minimum and maximum values, and individual data points are overlaid. Normal distributions were assumed for statistical analyses. Differences between experimental variables, and interactions between variables, were assessed by 3-way ANOVA (for data with three independent variables), 2-way ANOVA (for data with two independent variables), or mixed models (for repeated-measures analyses in which some data points were missing or had to be excluded for some mice). Where indicated, the Rout method (*Q* = 1%) was used to identify statistical outliers. For RNA-seq data, a hypergeometric test was used to determine if the genes differentially expressed between KO and WT mice showed significant overlap between diet-sex subgroups. Further details are provided within each figure legend. A *P*-value <0.05 (after adjustment for multiple comparisons) was considered statistically significant. *P*-values are either shown directly or are indicated by * (*p* < 0.05), ** (*p* < 0.01), or *** (*p* < 0.001).

## Supporting information

S1 Fig Adiponectin KO strategy and validation. **(A)** Details of modified *Adipoq* locus and KO strategy. Sperm containing the *Adipoq*^tm1a(KOMP)Wtsi^ allele (*tm1a*) was used to fertilize eggs from WT C57BL/6NCrl females. During the IVF process, recombinant Tat-Cre recombinase was also injected, causing conversion to the *Adipoq*^tm1a(KOMP)Wtsi^ allele (*tm1b*) by excision of exon 3 of the *Adipoq* gene. Gray boxes are exons (numbered above); *FRT*, flippase recognition target; *lacZ*, open reading frame for beta-galactosidase reporter gene; *loxP*, recombination site for Cre recombinase; *neo*, open reading frame for neomycin resistance gene. Adapted from https://www.komp.org. **(B–E)** Male and female WT and *Adipoq* KO mice were fed AL or a 30% CR diet from 9 to 13 weeks of age (0–4 weeks of CR). (B–C) Expression of exon 3 (B) or exons 1–2 (C) of *Adipoq* mRNA was determined by qPCR and is shown normalized to the geometric mean of *Tbp*, *Ppia*, and *Hprt* expression. (D–E) Adiponectin protein expression was determined by immunoblotting and quantified relative to ERK1/2 expression (loading control). Data in (B–C) are shown as box-and-whisker plots of 8 mice per group, except for male WT CR mice, for which there are 6 mice. Data in (D–E) are from six mice per group. Statistical analyses were done as described for Fig 1E. The underlying data for this figure can be found in the S1 Data file and S1 Raw Images.
(PDF)

S2 Fig Adiponectin KO does not alter the effect of CR on insulin sensitivity. Male and female WT and *Adipoq* KO mice were fed AL or CR as described for Fig 1. **(A)** At 12.5 weeks of age (3.5 weeks of AL or CR diet) mice underwent an oral glucose tolerance test (OGTT). HOMA-IR of mice calculated from glucose and insulin concentrations during the OGTT. **(B–C)** At 12 weeks of age (3 weeks of AL or CR diet) mice underwent an insulin tolerance test (ITT). Blood glucose concentrations during the ITT are shown for males (B) and females (C). Data presentation and statistical analysis are as described for Fig 1, with data from the following numbers of mice per group: *male WT AL*, *n* = 11 (A) or 13 (B); *male WT CR*, *n* = 14 (A) or 13 (B); *male KO AL*, *n* = 4 (A) or 11 (B); *male KO CR*, *n* = 4 (A) or 10 (B); *female WT AL*, *n* = 10 (A) or 7 (C); *female WT CR*, *n* = 12 (A) or 11 (C); *female KO AL*, *n* = 9 (A) or 11 (C); *female KO CR*, *n* = 7 (A) or 10 (C). The underlying data for this figure can be found in the S1 Data file.
(PDF)

S3 Fig Adiponectin KO decreases iWAT adipocyte size in males but not females. Male and female WT and *Adipoq* KO mice were fed AL or CR as described for Fig 1. At 13 weeks of age, mice were culled, and iWAT samples were collected. Micrographs of H&E-stained sections of iWAT **(A)** were used for histomorphometric analysis of adipocyte area **(B)**. Scale bars in (B) = 100 µm. Data presentation and statistical analysis are as described for Fig 3E and 3F. The underlying data for this figure can be found in the S1 Data file.
(PDF)

S4 Fig Effects of adiponectin KO on protein expression in gWAT. Male and female WT and *Adipoq* KO mice were fed AL or CR as described for Fig 1. At 13 weeks of age, mice were culled, and gWAT samples were collected. The indicated proteins were then analyzed by Western blotting: P-HSL and total HSL were analyzed on one pair of membranes while Perilipin A and CD36 were analyzed on a second pair; Coomassie total protein staining was used as a loading control and is shown separately for each membrane. **(B)** Quantification of the fluorescent Western blots **(A)** using LICOR and Image J software. Data presentation and statistical analysis are as described for Fig 1, with data from six mice per group. The underlying data for this figure can be found in the S1 Data file and S1 Raw Images.
(PDF)

S5 Fig Effects of adiponectin KO on gene expression in gWAT. Male and female WT and *Adipoq* KO mice were fed AL or CR as described for Fig 1. At 13 weeks of age, mice were culled, and gWAT was collected. Expression of the indicated transcripts was then determined by qPCR. Data presentation, numbers of mice per group, and statistical analyses are as described for Fig 3G. The underlying data for this figure can be found in the S1 Data file.
(PDF)

S6 Fig Adiponectin KO does not alter the effects of CR on liver mass or lipid accumulation. Male and female WT and *Adipoq* KO mice were fed AL or CR as described for Fig 1. At 13 weeks of age, mice were culled, and liver samples were collected. **(A)** Liver masses at necropsy, shown as box-and-whisker plots of the following numbers of mice per group: *male WT AL*, *n* = 35; *male WT CR*, *n* = 35; *male KO AL*, *n* = 26; *male KO CR*, *n* = 31; *female WT AL*, *n* = 36; *female WT CR*, *n* = 37; *female KO AL*, *n* = 31; *female KO CR*, *n* = 27. **(B)** Hepatic TG concentrations are shown as box-and-whisker plots of the following numbers of mice per group: *male WT AL*, *n* = 10; *male WT CR*, *n* = 9; *male KO AL*, *n* = 5; *male KO CR*, *n* = 9; *female WT AL*, *n* = 7; *female WT CR*, *n* = 8; *female KO AL*, *n* = 9; *female KO CR*, *n* = 7. **(C, D)** Micrographs of H&E-stained liver sections (C) were used for histomorphometric analysis of lipid droplet area (D), based on the white area in the micrographs; in (C), scale bar = 100 µm. Data in (D) are from five or six mice per group. In (B) and (D), outlier analyses (Rout method, *Q* = 1%) identified four data as statistical outliers: one KO CR female in (B) and (D), one WT CR female and one KO AL male in (D). These are excluded from (B) and (D) but are shown in the Source Data file. Statistical analyses are as described for Fig 1E. The underlying data for this figure can be found in the S1 Data file.
(PDF)

S7 Fig Adiponectin KO alters lipid metabolism-related genes in AL but not CR mice. Male and Female WT and *Adipoq* KO mice were fed AL or CR and their livers analyzed by bulk RNA-seq, as described for Fig 5. **(A, B)** Bubble plots of the top 10 GO gene sets (with lowest FDRq values) enriched in KO (NES > 0) or WT (NES < 0) within AL Male (A) or AL Female (B) subgroups. The GO terms are sorted by the NES values. Gene set names related to sterol metabolism are highlighted in orange, lipid catabolism in yellow **(C)** GSEA result between KO vs. WT in AL Male (upper left), AL Female (upper right), CR Male (lower left), or CR Female (lower right) subgroups for MM5828_GOBP_STEROL_BIOSYNTHETIC_PROCESS (left), MM4791_GOBP_FATTY_ACID_BIOSYNTHETIC_PROCESS (middle), and MM5199_GOBP_FATTY_ACID_CATABOLIC_PROCESS (right). For (A-C), data were extracted from GSEA results conducted with the M5 gene set library (m5.all.v2023.2.Mm.symbols.gmt). For (A, B) bubble size and color coding for FDRq values are shown below the bubble plots. In cases where the FDR-q value is 0, a value of 0.0001 has been used (−log10(FDRq) = 4) to allow visualization. **(D)** Expression of key genes in the MM4791_GOBP_FATTY_ACID_BIOSYNTHETIC_PROCESS gene set (*Fasn*, *Acaca*, and *Scd1*) based on normalized count data from liver RNAseq. To allow comparison across genes, the normalized counts are shown relative to the sex-genotype subgroup with the highest expression of each gene. Sample numbers for males and females are as described for Fig 5. Significant effects of genotype, sex and gene, and interactions thereof, were determined by 3-way ANOVA. The underlying data for this figure can be found in the S1 Data file.
(PDF)

S8 Fig Adiponectin KO leads to upregulated expression of genes related to amino acid catabolism under CR. Male and Female WT and *Adipoq* KO mice were fed AL or CR and their livers analyzed by bulk RNA-seq, as described for Fig 5. **(A, B)** Bubble plots of the top 10 GO gene sets (with lowest FDRq values) enriched in KO (NES > 0) or WT (NES < 0) within CR Male (D) or CR Female (E) subgroups. The GO terms are sorted by the NES values. Gene set names related to amino acid metabolism are highlighted in pink. Data were extracted from GSEA results conducted with the M5 gene set library (m5.all.v2023.2.Mm.symbols.gmt). For (A–B) and (D–E), bubble size and color coding for FDRq values are shown below the bubble plots. In cases where the FDR-q value is 0, a value of 0.0001 has been used (−log10(FDRq) = 4) to allow visualization. **(C,D)** Clustered heat map of the genes that showed core enrichment in MM10709_GOBP_ALPHA_AMINO_ACID_CATABOLIC_PROCESS gene set in comparisons between KO vs. WT for CR Male (A) or CR Female (B) subgroups. The common genes between (C) and (D) are shown in bold. Sample numbers for liver RNA-seq are as follows: *Male WT AL*, *n* = 6; *Male WT CR*, *n* = 5; *Male KO AL*, *n* = 5; *Male KO CR*, *n* = 5; *Female WT AL*, *n* = 5; *Female WT CR*, *n* = 6; *Female KO AL*, *n* = 6; *Female KO CR*, *n* = 6. The underlying data for this figure can be found in the S1 Data file.
(PDF)

S9 Fig Adiponectin KO does not alter muscle mass under AL nor CR diet. Male and female WT and *Adipoq* KO mice were fed AL or CR as described for Fig 1. At 13 weeks of age, mice were culled, and SkM samples were collected. **(A–D)** Gastrocnemius and soleus masses were recorded at necropsy (13 weeks of age) and are shown as absolute mass (A and B) or % body mass (C and D). Data areown as box-and-whisker plots of the following numbers of mice per group *male WT AL*, *n* = 26; *male WT CR*, *n* = 28; *male KO AL*, *n* = 21; *male KO CR*, *n* = 23 for gastrocnemius or 22 for soleus; *female WT AL*, *n* = 31 for gastrocnemius or 30 for soleus; *female WT CR*, *n* = 30 for gastrocnemius or 29 for soleus; *female KO AL*, *n* = 22 for gastrocnemius or 23 for soleus; *female KO CR*, *n* = 19 for gastrocnemius or 18 for soleus. Statistical analyses were as described for Fig 1E. The underlying data for this figure can be found in the S1 Data file.
(PDF)

S10 Fig Adiponectin KO does not alter muscle fiber CSA in gastrocnemius under AL nor CR diet. Male and female WT and *Adipoq* KO mice were fed AL or CR as described for Fig 1. At 13 weeks of age, mice were culled, and SkM samples were collected. **(A)** CSA of gastrocnemius mass at the cull. **(B)** Representative images of gastrocnemius cross-sectional sections. Scale bars indicate 100 μm. Data are shown as box-and-whisker plots of the following numbers of mice per group *male WT AL*, *n* = 5; *male WT CR*, *n* = 6; *male KO AL*, *n* = 4; *male KO CR*, *n* = 6; *female WT AL*, *n* = 6; *female WT CR*, *n* = 6; *female KO AL*, *n* = 5; *female KO CR*, *n* = 5. Statistical analyses were as described for Fig 1E. The underlying data for this figure can be found in the S1 Data file.
(PDF)

S11 Fig Effects of adiponectin KO on hepatic expression of gluconeogenesis-related genes and on glycerol-driven gluconeogenesis. Male and female WT and *Adipoq* KO mice were fed AL or CR and their livers analyzed by bulk RNA-seq, as described for Fig 5. **(A–E)** Normalized count data for *Gpd1* (A), *Gpd2* (B), *Got1* (C), *Sds* (D), and *Fbp2* (E); for space reasons, the y-axes for (C) and (D) show counts in thousands (k). Sample numbers for males and females are as described for Fig 5. Statistical analyses are as described for Fig 1E. **(F–G)** Glycerol tolerance tests in male (F) and female (G) mice, shown as mean ± SEM of the following numbers of mice per group: *males*, *n* = 14 (WT AL, WT CR), 10 (KO AL), or 11 (KO CR); *females*, *n* = 16 (WT AL), 14 (WT CR), 10 (KO AL), or 7 (KO CR). Significant effects of diet, time, and/or genotype, and interactions thereof, were determined by 3-way ANOVA. Within each diet and sex, significant genotype effects at each time point were determined by 2-way ANOVA with Šidák’s multiple comparisons test; ** (*P* < 0.01). The underlying data for this figure can be found in the S1 Data file.
(PDF)

S1 Table Ceramide concentrations for different sphingolipid species. The table shows LC–MS-measured concentrations of total and each species of ceramide. Average ± SEM are shown. Outlier identification analysis was conducted with the Rout method (*Q* = 1%) and three outliers were excluded from the data for Ceramide 26:0. The underlying data for this table can be found in the S1 Data file.
(PDF)

S2 Table DHC concentrations for different sphingolipid species. The table shows LC–MS-measured concentrations of total and each species of DHC. Average ± SEM are shown. DHC 26:0 was not detected for 7 samples and DHC 26:1 was not detected for 1 sample. The cells for those values were left blank. The underlying data for this table can be found in the S1 Data file.
(PDF)

S3 Table Ceramide:DHC ratio for different sphingolipid species. The table shows Ceramide:DHC ratio based on LC–MS-measured concentrations of total and each species of ceramide (S1 Table) and DHC (S2 Table). Average ± SEM are shown. Outlier identification analysis was conducted with the Rout method (*Q* = 1%) and three outliers were excluded from the data for Ceramide 26:0. The Ceramide:DHC ratio data for these three samples were also excluded. DHC 26:0 was not detected for 7 samples and DHC 26:1 was not detected for 1 sample, and the corresponding ceramide:DHC ratio values were left as blank. The underlying data for this table can be found in the S1 Data file.
(PDF)

S4 Table Genes not included in the volcano plots in Fig 5B. The list of genes not included in the volcano plots in Fig 5B because the absolute log2FoldChange is over 10 or −log10(pvalue) is over 7.5. For CR/Females no genes meet these criteria (“n/a/”). The underlying data for this table can be found in the S1 Data file, in the sheets relating to Fig 5B.
(PDF)

S5 Table Amino acid concentrations in the liver. Hepatic amino acid concentrations (ng/g) were measured by LC–MS/MS. Data for each group (sex/diet/genotype) are shown as average ± SEM. *P* values from 3-way ANOVA are shown for effects of each independent variable and their interactions. Asp, Gln, and Tyr were not detectable.
(PDF)

S6 Table Amino acid concentrations in plasma. Plams amino acid concentrations (ng/g) were measured by LC–MS/MS. Data for each group (sex/diet/genotype) are shown as average ± SEM. *P* values from 3-way ANOVA are shown for effects of each independent variable and their interactions. Asp, Gln, and Tyr were not detectable.
(PDF)

S7 Table Reagents and resources used in this study. (PDF)

S1 Raw Images Raw images for western blots used in Figs 1B, S1D, and S4A. The blots for Fig 1B (plasma adiponectin and total protein) were captured using a LiCor Odyssey CLx scanner, whereas those for S1B and S4A Figs were captured using a LiCor Odyssey M scanner. For S1B and S4A Figs, most membranes were cut before incubating in primary antibodies, allowing multiple separate incubations in different antibodies at the same time; the location of the cuts, where visible, are indicated by * in the images and, for S4B, are clear in the Coomassie scans. Because of this, the original, uncropped scans for each antibody cover only a portion of the full membrane. Note that for S4A Fig, membrane 1 was cut before incubating in primary antibodies whereas membrane 2 was first imaged for Perilipin A and was then cut between 50 and 75 kDa, with the upper portion then incubated in anti-CD36 Ab. Perilipin A used a goat secondary antibody, which visualized the 75 kDa marker but not the 50 kDa marker.
(PDF)

S1 Data Raw Data underlying Figs 1A, 1C–1E, 2A–2F, 3A–3D, 3F, 3G, 4A–4F, 5A–5H, S1B, S1C, S1E, S2A–S2C, S3A, S4B, S5A, S5B, S6A, S6B, S6D, S7A–S7D, S8A–S8D, S9A–S9D, S10A, and S11A–S11G and S1–S3 Tables. (XLSX)

## Abbreviations

AL: *ad libitum*
BAT: brown adipose tissue
BCAAs: branched-chain amino acids
CR: caloric restriction
CSA: cross-sectional area
DHC: dihydroceramides
EE: energy expenditure
GO: gene ontology
GSEA: gene set enrichment analysis
H&E: hematoxylin and eosin
HOMA-IR: Homeostatic Model Assessment for Insulin Resistance
HSL: hormone-sensitive lipase
IVF: in vitro fertilization
KO: knockout
KOMP: Knockout Mouse Project
LC–MS/MS: liquid chromatography tandem mass spectrometry
MRM: multiple reaction monitoring
NEFA: non-esterified fatty acid
OGTT: oral glucose tolerance test
OLTT: oral lipid tolerance tests
PCA: principal component analysis
RER: respiratory exchange ratio
RIN: RNA Integrity Number
SkM: skeletal muscle
tAUC: total area under the curve
TG: triacylglycerol
WAT: white adipose tissue
WT: wild-type
