# Supplementary material for: Adiponectin exerts sex-dependent effects on lipid, amino acid, and glucose metabolism during caloric restriction
Source: PLoS Biol. 2026 Jun 18;24(6):e3003821. doi: 10.1371/journal.pbio.3003821 (PMC13278438; doi:10.1371/journal.pbio.3003821)
Supplement: S1 Fig — (A) Details of modified Adipoq locus and KO strategy. Sperm containing the Adipoqtm1a(KOMP)Wtsi allele (tm1a) was used to fertilize eggs from WT C57BL/6NCrl females. During the IVF process, recombinant Tat-Cre recombinase was also injected, causing conversion to the Adipoqtm1a(KOMP)Wtsi allele (tm1b) by excision of exon 3 of the Adipoq gene. Gray boxes are exons (numbered above); FRT, flippase recognition target; lacZ, open reading frame for beta-galactosidase reporter gene; loxP, recombination site for Cre recombinase; neo, open reading frame for neomycin resistance gene. Adapted from https://www.komp.org. (B–E) Male and female WT and Adipoq KO mice were fed AL or a 30% CR diet from 9 to 13 weeks of age (0–4 weeks of CR). (B–C) Expression of exon 3 (B) or exons 1–2 (C) of Adipoq mRNA was determined by qPCR and is shown normalized to the geometric mean of Tbp, Ppia, and Hprt expression. (D–E) Adiponectin protein expression was determined by immunoblotting and quantified relative to ERK1/2 expression (loading control). Data in (B–C) are shown as box-and-whisker plots of 8 mice per group, except for male WT CR mice, for which there are 6 mice. Data in (D–E) are from six mice per group. Statistical analyses were done as described for Fig 1E. The underlying data for this figure can be found in the S1 Data file and S1 Raw Images. (PDF) [file pbio.3003821.s001.pdf]

S1 Figure

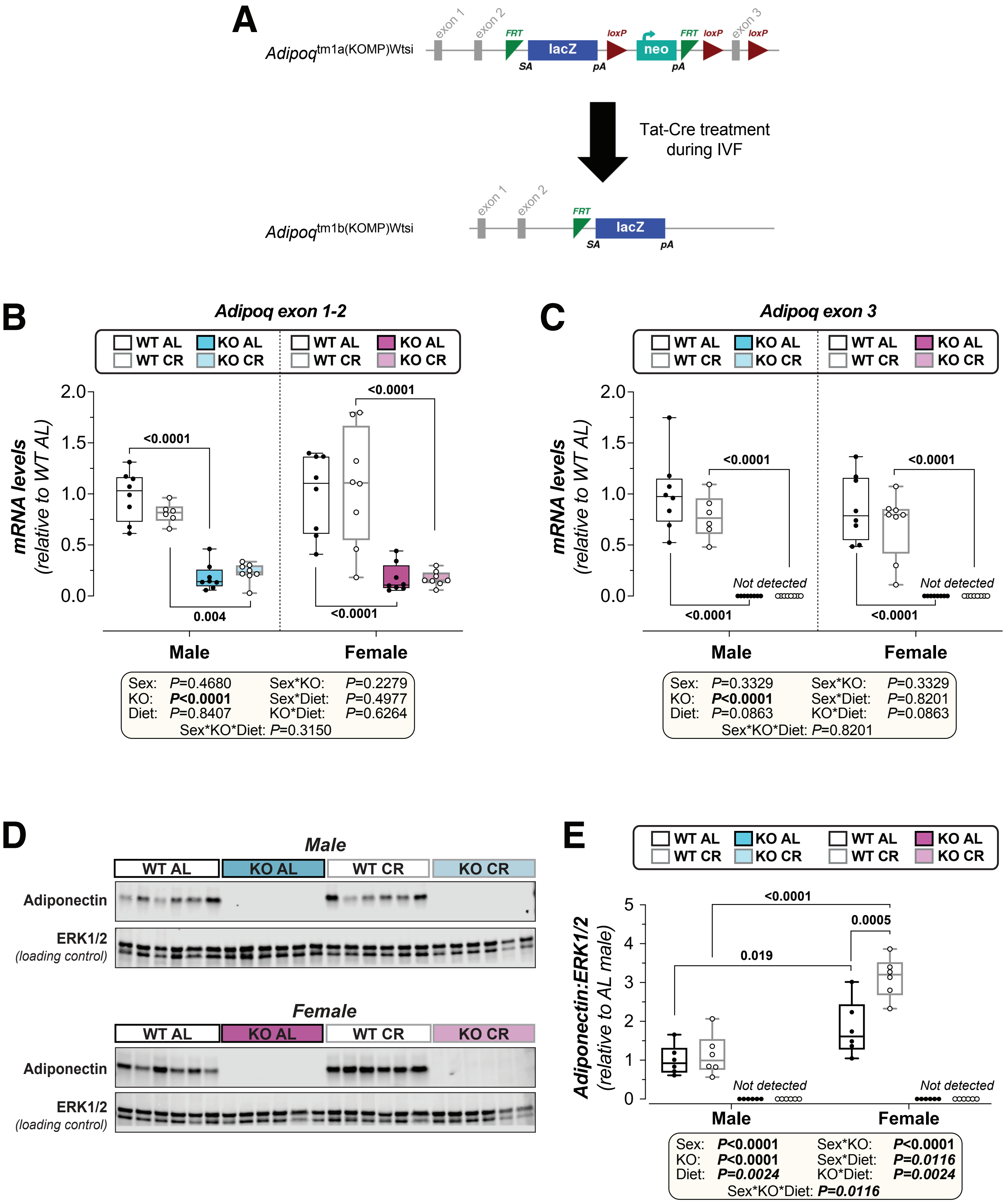

A
Adipoqtm1a(KOMP)Wtsi
exon 1
exon 2
FRT
lacZ
loxP
neo
FRT
loxP
exon 3
loxP
SA
pA
pA
Tat-Cre treatment during IVF
Adipoqtm1b(KOMP)Wtsi
exon 1
exon 2
FRT
lacZ
SA
pA
B
Adipoq exon 1-2
WT AL
KO AL
WT CR
KO CR
mRNA levels (relative to WT AL)
<0.0001
0.004
<0.0001
<0.0001
Male
Female
Sex: P=0.4680 Sex*KO: P=0.2279
KO: P<0.0001 Sex*Diet: P=0.4977
Diet: P=0.8407 KO*Diet: P=0.6264
Sex*KO*Diet: P=0.3150
C
Adipoq exon 3
mRNA levels (relative to WT AL)
<0.0001
<0.0001
<0.0001
<0.0001
Not detected
Not detected
Male
Female
Sex: P=0.3329 Sex*KO: P=0.3329
KO: P<0.0001 Sex*Diet: P=0.8201
Diet: P=0.0863 KO*Diet: P=0.0863
Sex*KO*Diet: P=0.8201
D
Male
WT AL
KO AL
WT CR
KO CR
Adiponectin
ERK1/2 (loading control)
Female
WT AL
KO AL
WT CR
KO CR
Adiponectin
ERK1/2 (loading control)
E
Adiponectin:ERK1/2 (relative to AL male)
<0.0001
0.0005
0.019
Not detected
Not detected
Male
Female
Sex: P<0.0001 Sex*KO: P<0.0001
KO: P<0.0001 Sex*Diet: P=0.0116
Diet: P=0.0024 KO*Diet: P=0.0024
Sex*KO*Diet: P=0.0116

S1 Fig. Adiponectin KO strategy and validation. (A) Details of modified *Adipoq* locus and KO strategy. Sperm containing the *Adipoq*$^{tm1a(KOMP)Wtsi}$ allele (*tm1a*) was used to fertilise eggs from WT C57BL/6NCrl females. During the IVF process, recombinant Tat-Cre recombinase was also injected, causing conversion to the *Adipoq*$^{tm1a(KOMP)Wtsi}$ allele (*tm1b*) by excision of exon 3 of the *Adipoq* gene. Grey boxes are exons (numbered above); *FRT*, flippase recognition target; *lacZ*, open reading frame for beta-galactosidase reporter gene; *loxP,* recombination site for Cre recombinase; *neo,* open reading frame for neomycin resistance gene. Adapted from www.komp.org. **(B-E)** Male and female WT and *Adipoq* KO mice were fed AL or a 30% CR diet from 9-13 weeks of age (0-4 weeks of CR). (B-C) Expression of exon 3 (B) or exons 1-2 (C) of *Adipoq* mRNA was determined by qPCR and is shown normalised to the geometric mean of *Tbp, Ppia* and *Hprt* expression. (D-E) Adiponectin protein expression was determined by immunoblotting and quantified relative to ERK1/2 expression (loading control). Data in (B-C) are shown as box-and-whisker plots of 8 mice per group, except for male WT CR mice, for which there are 6 mice. Data in (D-E) are from six mice per group. Statistical analyses were done as described for Fig 1E. The underlying data for this figure can be found in the S1_Data file and S1_Raw_Images.
