# Supplementary material for: Adiponectin exerts sex-dependent effects on lipid, amino acid, and glucose metabolism during caloric restriction
Source: PLoS Biol. 2026 Jun 18;24(6):e3003821. doi: 10.1371/journal.pbio.3003821 (PMC13278438; doi:10.1371/journal.pbio.3003821)
Supplement: S2 Fig — Male and female WT and Adipoq KO mice were fed AL or CR as described for Fig 1. (A) At 12.5 weeks of age (3.5 weeks of AL or CR diet) mice underwent an oral glucose tolerance test (OGTT). HOMA-IR of mice calculated from glucose and insulin concentrations during the OGTT. (B–C) At 12 weeks of age (3 weeks of AL or CR diet) mice underwent an insulin tolerance test (ITT). Blood glucose concentrations during the ITT are shown for males (B) and females (C). Data presentation and statistical analysis are as described for Fig 1, with data from the following numbers of mice per group: male WT AL, n = 11 (A) or 13 (B); male WT CR, n = 14 (A) or 13 (B); male KO AL, n = 4 (A) or 11 (B); male KO CR, n = 4 (A) or 10 (B); female WT AL, n = 10 (A) or 7 (C); female WT CR, n = 12 (A) or 11 (C); female KO AL, n = 9 (A) or 11 (C); female KO CR, n = 7 (A) or 10 (C). The underlying data for this figure can be found in the S1 Data file. (PDF) [file pbio.3003821.s002.pdf]

S2 Figure

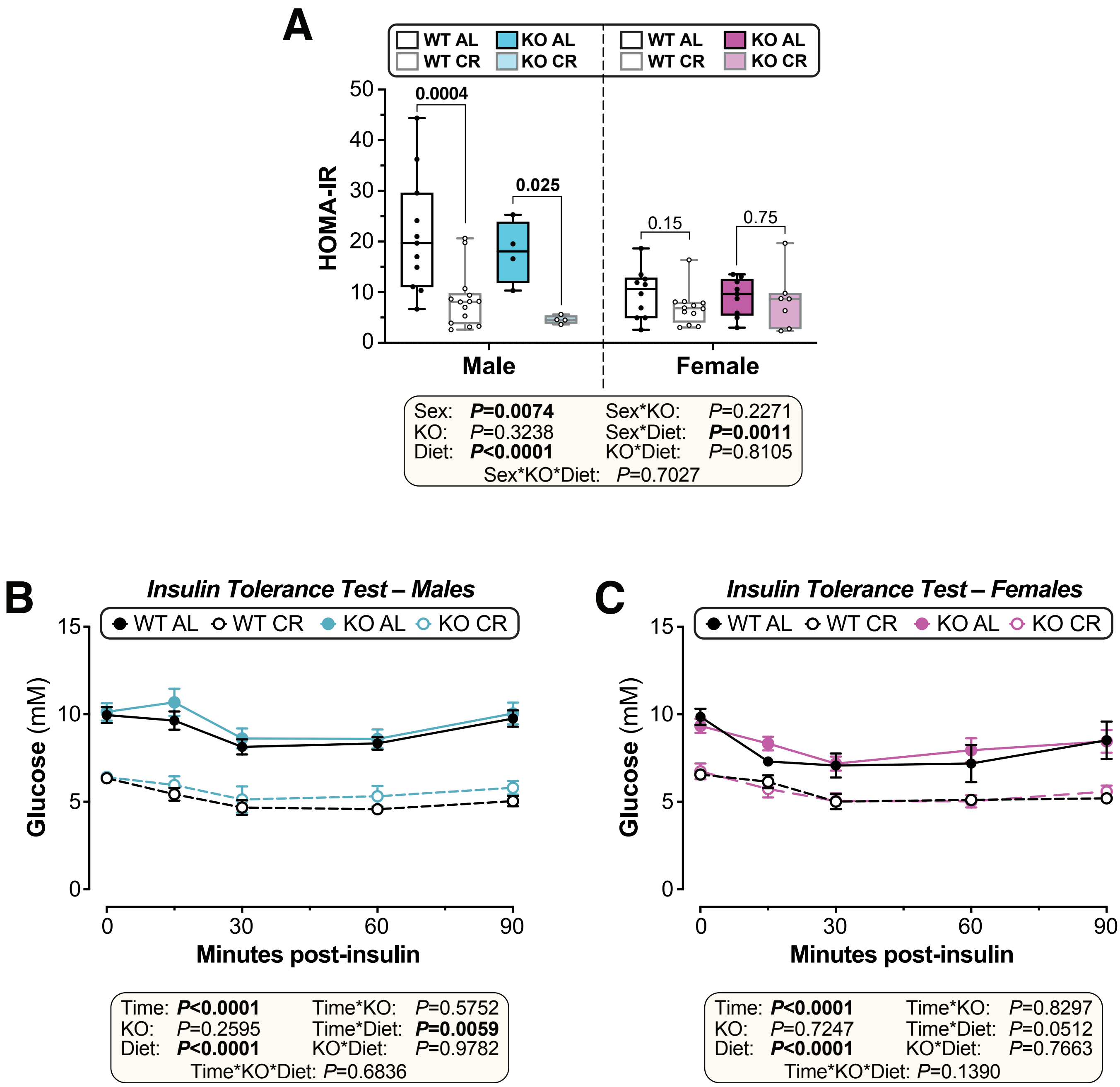

S2 Fig. Adiponectin KO does not alter the effect of CR on insulin sensitivity. Male and female WT and *Adipoq* KO mice were fed AL or CR as described for Fig 1. **(A)** At 12.5 weeks of age (3.5 weeks of AL or CR diet) mice underwent an oral glucose tolerance test (OGTT). HOMA-IR of mice calculated from glucose and insulin concentrations during the OGTT. **(B-C)** At 12 weeks of age (3 weeks of AL or CR diet) mice underwent an insulin tolerance test (ITT). Blood glucose concentrations during the ITT are shown for males (B) and females (C). Data presentation and statistical analysis are as described for Fig 1, with data from the following numbers of mice per group: *male WT AL*, n= 11 (A) or 13 (B); *male WT CR,* n= 14 (A) or 13 (B); *male KO AL,* n= 4 (A) or 11 (B); *male KO CR,* n= 4 (A) or 10 (B); *female WT AL*, n= 10 (A) or 7 (C); *female WT CR,* n= 12 (A) or 11 (C); *female KO AL,* n= 9 (A) or 11 (C); *female KO CR,* n= 7 (A) or 10 (C). The underlying data for this figure can be found in the S1_Data file.
