# Supplementary material for: Adiponectin exerts sex-dependent effects on lipid, amino acid, and glucose metabolism during caloric restriction
Source: PLoS Biol. 2026 Jun 18;24(6):e3003821. doi: 10.1371/journal.pbio.3003821 (PMC13278438; doi:10.1371/journal.pbio.3003821)
Supplement: S3 Fig — Male and female WT and Adipoq KO mice were fed AL or CR as described for Fig 1. At 13 weeks of age, mice were culled, and iWAT samples were collected. Micrographs of H&E-stained sections of iWAT (A) were used for histomorphometric analysis of adipocyte area (B). Scale bars in (B) = 100 µm. Data presentation and statistical analysis are as described for Fig 3E and 3F. The underlying data for this figure can be found in the S1 Data file. (PDF) [file pbio.3003821.s003.pdf]

S3 Figure

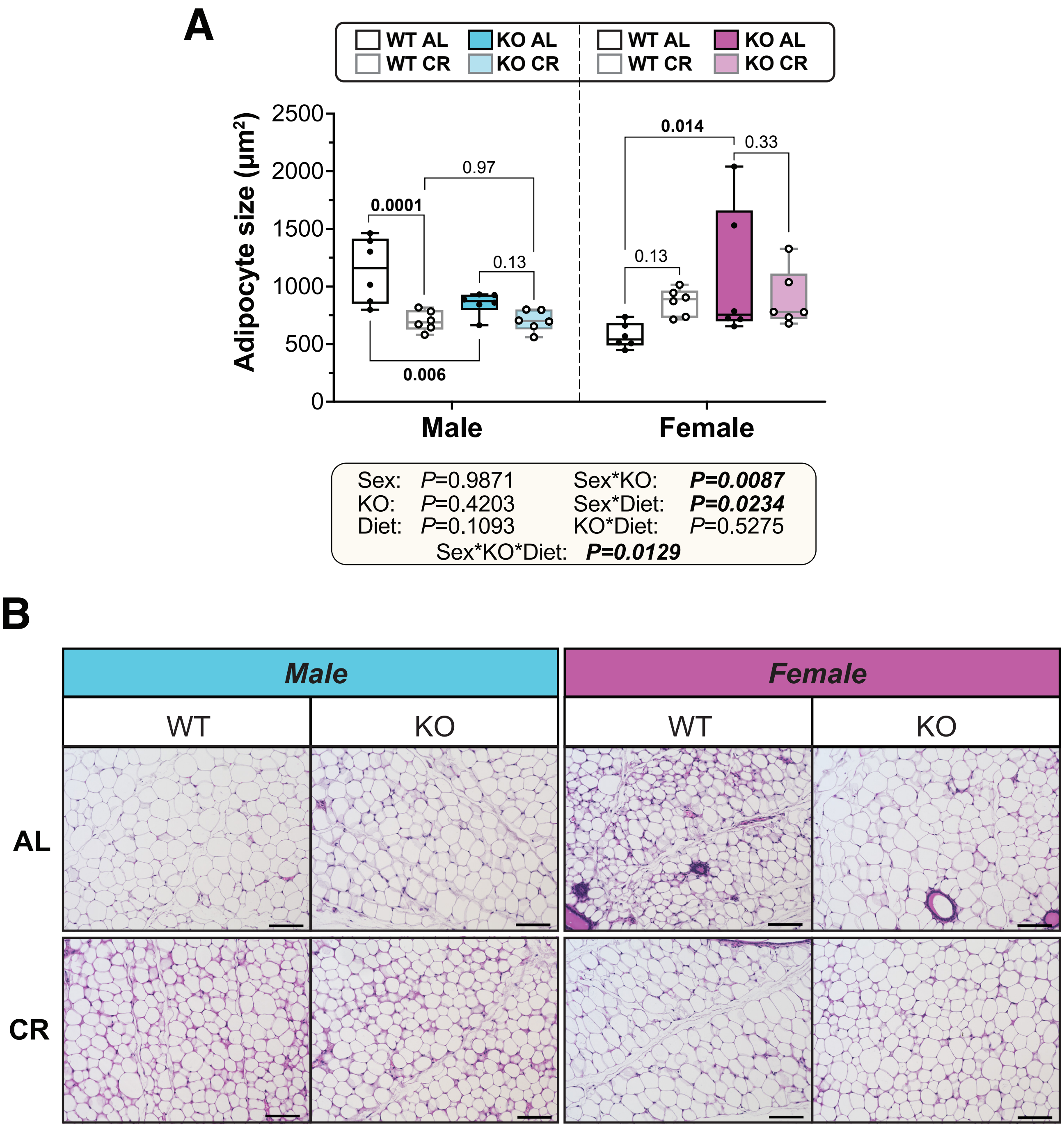

S3 Fig. Adiponectin KO decreases iWAT adipocyte size in males but not females. Male and female WT and *Adipoq* KO mice were fed AL or CR as described for Fig 1. At 13 weeks of age, mice were culled and iWAT samples were collected. Micrographs of H&E-stained sections of iWAT (A) were used for histomorphometric analysis of adipocyte area (B). Scale bars in (B) = 100 µm. Data presentation and statistical analysis are as described for Fig 3E-F. The underlying data for this figure can be found in the S1_Data file.
