# Supplementary material for: Adiponectin exerts sex-dependent effects on lipid, amino acid, and glucose metabolism during caloric restriction
Source: PLoS Biol. 2026 Jun 18;24(6):e3003821. doi: 10.1371/journal.pbio.3003821 (PMC13278438; doi:10.1371/journal.pbio.3003821)
Supplement: S4 Fig — Male and female WT and Adipoq KO mice were fed AL or CR as described for Fig 1. At 13 weeks of age, mice were culled, and gWAT samples were collected. The indicated proteins were then analyzed by Western blotting: P-HSL and total HSL were analyzed on one pair of membranes while Perilipin A and CD36 were analyzed on a second pair; Coomassie total protein staining was used as a loading control and is shown separately for each membrane. (B) Quantification of the fluorescent Western blots (A) using LICOR and Image J software. Data presentation and statistical analysis are as described for Fig 1, with data from six mice per group. The underlying data for this figure can be found in the S1 Data file and S1 Raw Images. (PDF) [file pbio.3003821.s004.pdf]

S4 Figure

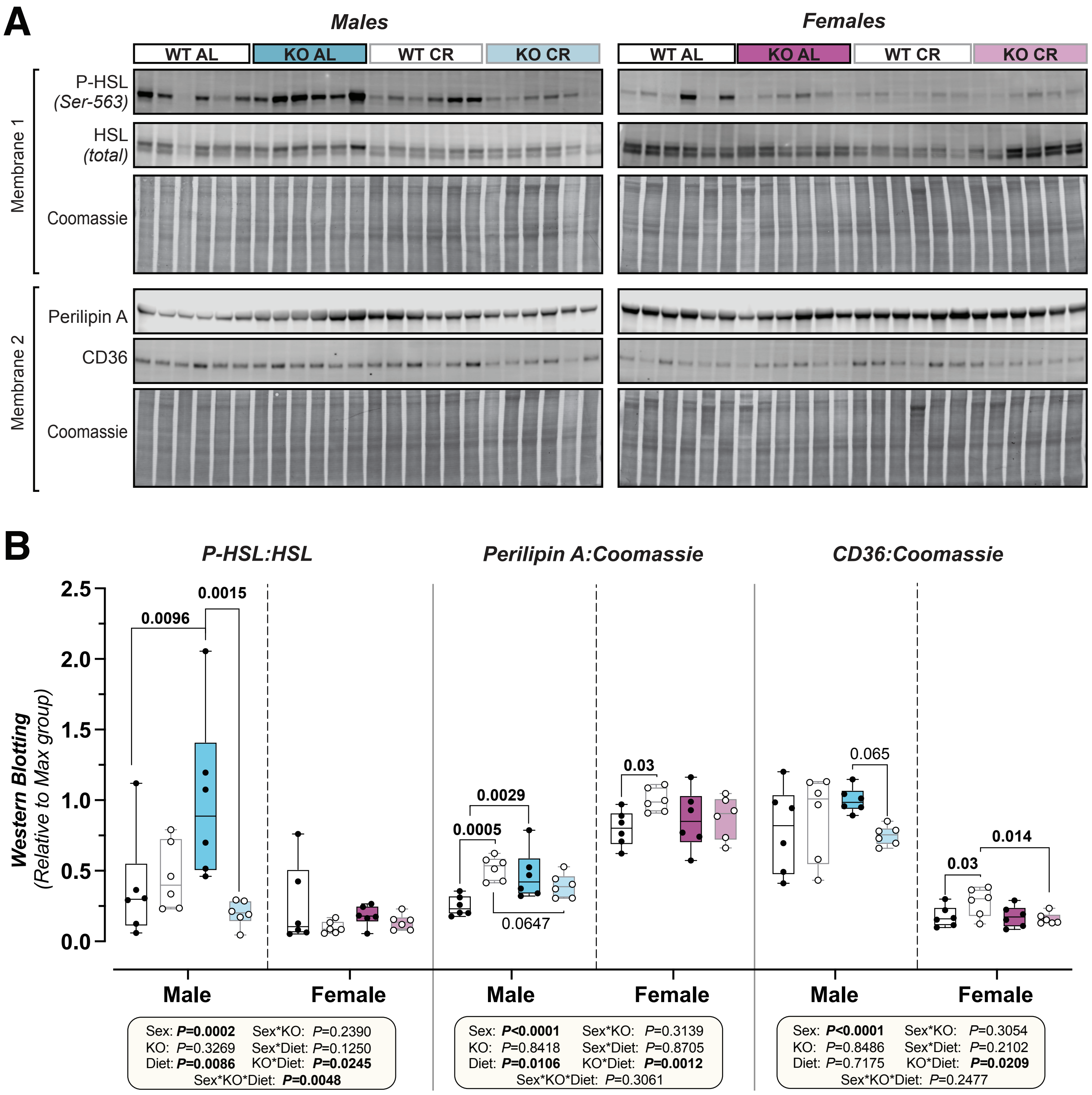

S4 Fig. Effects of adiponectin KO on protein expression in gWAT. Male and female WT and *Adipoq* KO mice were fed AL or CR as described for Fig 1. At 13 weeks of age, mice were culled and gWAT samples were collected. The indicated proteins were then analysed by Western blotting: P-HSL and total HSL were analysed on one pair of membranes while Perilipin A and CD36 were analysed on a second pair; Coomassie total protein staining was used as a loading control and is shown separately for each membrane. (B) Quantification of the fluorescent Western blots (A) using LICOR and Image J software. Data presentation and statistical analysis are as described for Fig 1, with data from six mice per group. The underlying data for this figure can be found in the S1_Data file and S1_Raw_Images.
