# Supplementary material for: Adiponectin exerts sex-dependent effects on lipid, amino acid, and glucose metabolism during caloric restriction
Source: PLoS Biol. 2026 Jun 18;24(6):e3003821. doi: 10.1371/journal.pbio.3003821 (PMC13278438; doi:10.1371/journal.pbio.3003821)
Supplement: S5 Fig — Male and female WT and Adipoq KO mice were fed AL or CR as described for Fig 1. At 13 weeks of age, mice were culled, and gWAT was collected. Expression of the indicated transcripts was then determined by qPCR. Data presentation, numbers of mice per group, and statistical analyses are as described for Fig 3G. The underlying data for this figure can be found in the S1 Data file. (PDF) [file pbio.3003821.s005.pdf]

S5 Figure

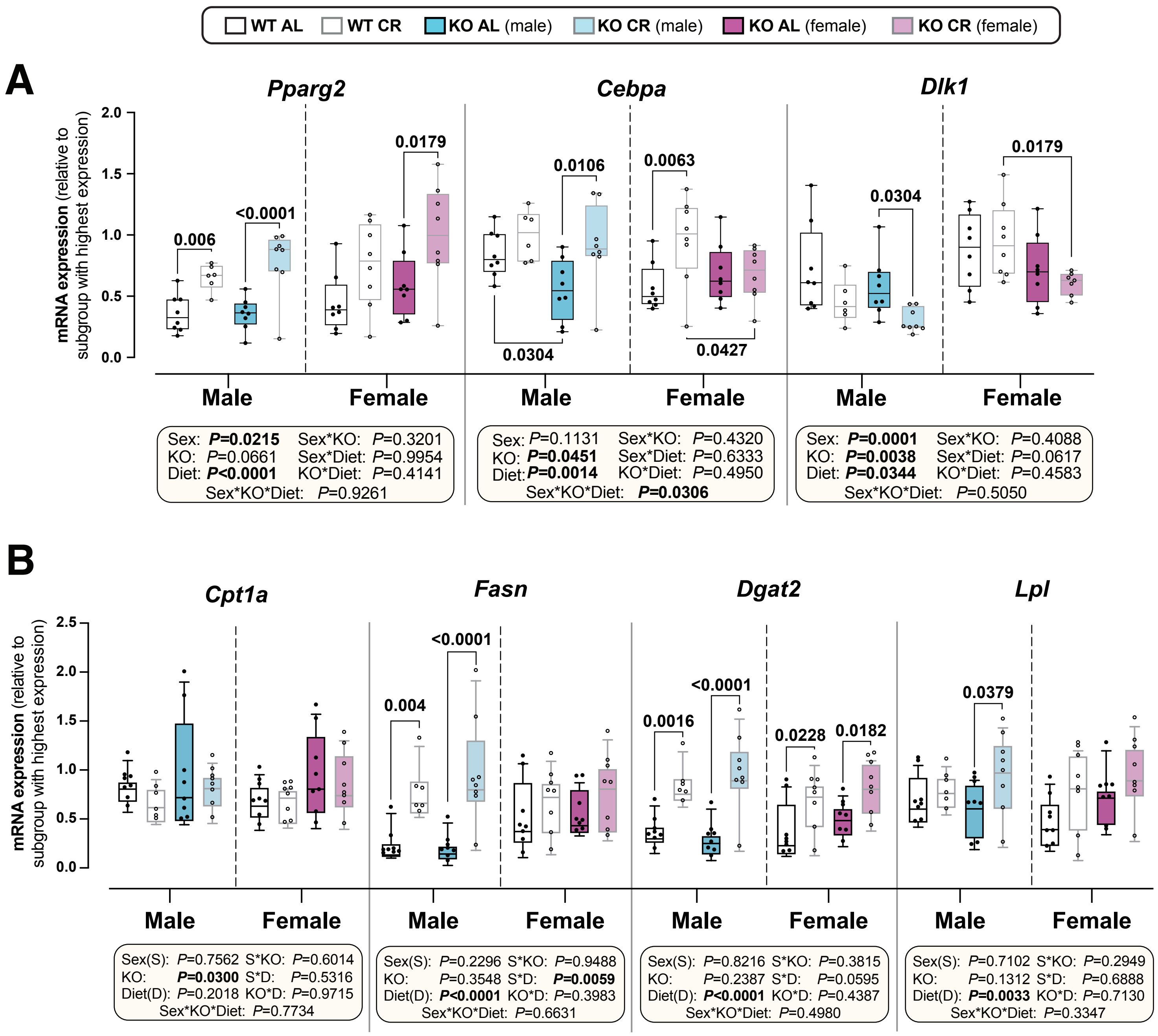

S5 Fig. Effects of adiponectin KO on gene expression in gWAT. Male and female WT and *Adipoq* KO mice were fed AL or CR as described for Fig 1. At 13 weeks of age, mice were culled and gWAT was collected. Expression of the indicated transcripts was then determined by qPCR. Data presentation, numbers of mice per group, and statistical analyses are as described for Fig 3G. The underlying data for this figure can be found in the S1_Data file.
