# Supplementary material for: Adiponectin exerts sex-dependent effects on lipid, amino acid, and glucose metabolism during caloric restriction
Source: PLoS Biol. 2026 Jun 18;24(6):e3003821. doi: 10.1371/journal.pbio.3003821 (PMC13278438; doi:10.1371/journal.pbio.3003821)
Supplement: S6 Fig — Male and female WT and Adipoq KO mice were fed AL or CR as described for Fig 1. At 13 weeks of age, mice were culled, and liver samples were collected. (A) Liver masses at necropsy, shown as box-and-whisker plots of the following numbers of mice per group: male WT AL, n = 35; male WT CR, n = 35; male KO AL, n = 26; male KO CR, n = 31; female WT AL, n = 36; female WT CR, n = 37; female KO AL, n = 31; female KO CR, n = 27. (B) Hepatic TG concentrations are shown as box-and-whisker plots of the following numbers of mice per group: male WT AL, n = 10; male WT CR, n = 9; male KO AL, n = 5; male KO CR, n = 9; female WT AL, n = 7; female WT CR, n = 8; female KO AL, n = 9; female KO CR, n = 7. (C, D) Micrographs of H&E-stained liver sections (C) were used for histomorphometric analysis of lipid droplet area (D), based on the white area in the micrographs; in (C), scale bar = 100 µm. Data in (D) are from five or six mice per group. In (B) and (D), outlier analyses (Rout method, Q = 1%) identified four data as statistical outliers: one KO CR female in (B) and (D), one WT CR female and one KO AL male in (D). These are excluded from (B) and (D) but are shown in the Source Data file. Statistical analyses are as described for Fig 1E. The underlying data for this figure can be found in the S1 Data file. (PDF) [file pbio.3003821.s006.pdf]

S6 Figure

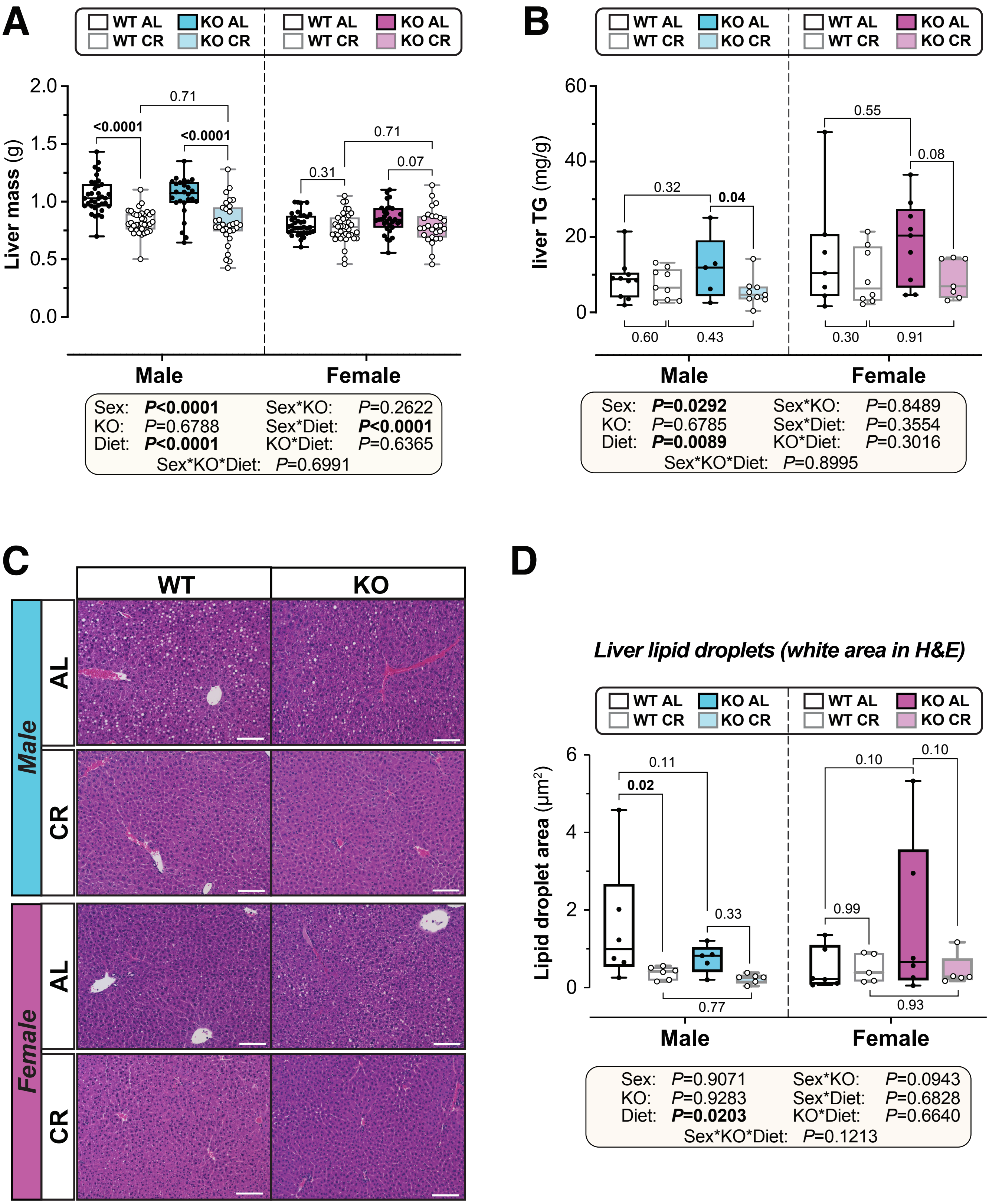

A
WT AL
KO AL
WT CR
KO CR
Liver mass (g)
<0.0001
<0.0001
0.71
0.31
0.07
Male
Female
Sex: P<0.0001
KO: P=0.6788
Diet: P<0.0001
Sex*KO: P=0.2622
Sex*Diet: P<0.0001
KO*Diet: P=0.6365
Sex*KO*Diet: P=0.6991
B
liver TG (mg/g)
0.32
0.04
0.60
0.43
0.55
0.08
0.30
0.91
Sex: P=0.0292
KO: P=0.6785
Diet: P=0.0089
Sex*KO: P=0.8489
Sex*Diet: P=0.3554
KO*Diet: P=0.3016
Sex*KO*Diet: P=0.8995
C
WT
KO
Male
Female
AL
CR
D
Liver lipid droplets (white area in H&E)
Lipid droplet area (μm²)
0.11
0.02
0.33
0.77
0.10
0.10
0.99
0.93
Sex: P=0.9071
KO: P=0.9283
Diet: P=0.0203
Sex*KO: P=0.0943
Sex*Diet: P=0.6828
KO*Diet: P=0.6640
Sex*KO*Diet: P=0.1213

S6 Fig. Adiponectin KO does not alter the effects of CR on liver mass or lipid accumulation. Male and female WT and *Adipoq* KO mice were fed AL or CR as described for Fig 1. At 13 weeks of age, mice were culled, and liver samples were collected. **(A)** Liver masses at necropsy, shown as box-and-whisker plots of the following numbers of mice per group: *male WT AL*, n=35 ; *male WT CR,* n=35; *male KO AL,* n=26; *male KO CR,* n=31; *female WT AL*, n=36; *female WT CR,* n=37; *female KO AL,* n=31; *female KO CR,* n=27. **(B)** Hepatic TG concentrations are shown as box-and-whisker plots of the following numbers of mice per group: *male WT AL*, n=10; *male WT CR,* n=9; *male KO AL,* n=5; *male KO CR,* n=9; *female WT AL*, n=7; *female WT CR,* n=8; *female KO AL,* n=9; *female KO CR,* n=7. **(C,D)** Micrographs of H&E-stained liver sections (C) were used for histomorphometric analysis of lipid droplet area (D), based on the white area in the micrographs; in (C), scale bar = 100 µm. Data in (D) are from five or six mice per group. In (B) and (D), outlier analyses (Rout method, Q = 1%) identified four data as statistical outliers: one KO CR female in (B) and (D), one WT CR female and one KO AL male in (D). These are excluded from (B) and (D) but are shown in the Source Data file. Statistical analyses are as described for Fig 1E. The underlying data for this figure can be found in the S1_Data file.
