# Supplementary material for: Adiponectin exerts sex-dependent effects on lipid, amino acid, and glucose metabolism during caloric restriction
Source: PLoS Biol. 2026 Jun 18;24(6):e3003821. doi: 10.1371/journal.pbio.3003821 (PMC13278438; doi:10.1371/journal.pbio.3003821)
Supplement: S7 Fig — Male and Female WT and Adipoq KO mice were fed AL or CR and their livers analyzed by bulk RNA-seq, as described for Fig 5. (A, B) Bubble plots of the top 10 GO gene sets (with lowest FDRq values) enriched in KO (NES > 0) or WT (NES < 0) within AL Male (A) or AL Female (B) subgroups. The GO terms are sorted by the NES values. Gene set names related to sterol metabolism are highlighted in orange, lipid catabolism in yellow (C) GSEA result between KO vs. WT in AL Male (upper left), AL Female (upper right), CR Male (lower left), or CR Female (lower right) subgroups for MM5828_GOBP_STEROL_BIOSYNTHETIC_PROCESS (left), MM4791_GOBP_FATTY_ACID_BIOSYNTHETIC_PROCESS (middle), and MM5199_GOBP_FATTY_ACID_CATABOLIC_PROCESS (right). For (A-C), data were extracted from GSEA results conducted with the M5 gene set library (m5.all.v2023.2.Mm.symbols.gmt). For (A, B) bubble size and color coding for FDRq values are shown below the bubble plots. In cases where the FDR-q value is 0, a value of 0.0001 has been used (−log10(FDRq) = 4) to allow visualization. (D) Expression of key genes in the MM4791_GOBP_FATTY_ACID_BIOSYNTHETIC_PROCESS gene set (Fasn, Acaca, and Scd1) based on normalized count data from liver RNAseq. To allow comparison across genes, the normalized counts are shown relative to the sex-genotype subgroup with the highest expression of each gene. Sample numbers for males and females are as described for Fig 5. Significant effects of genotype, sex and gene, and interactions thereof, were determined by 3-way ANOVA. The underlying data for this figure can be found in the S1 Data file. (PDF) [file pbio.3003821.s007.pdf]

S7 Figure

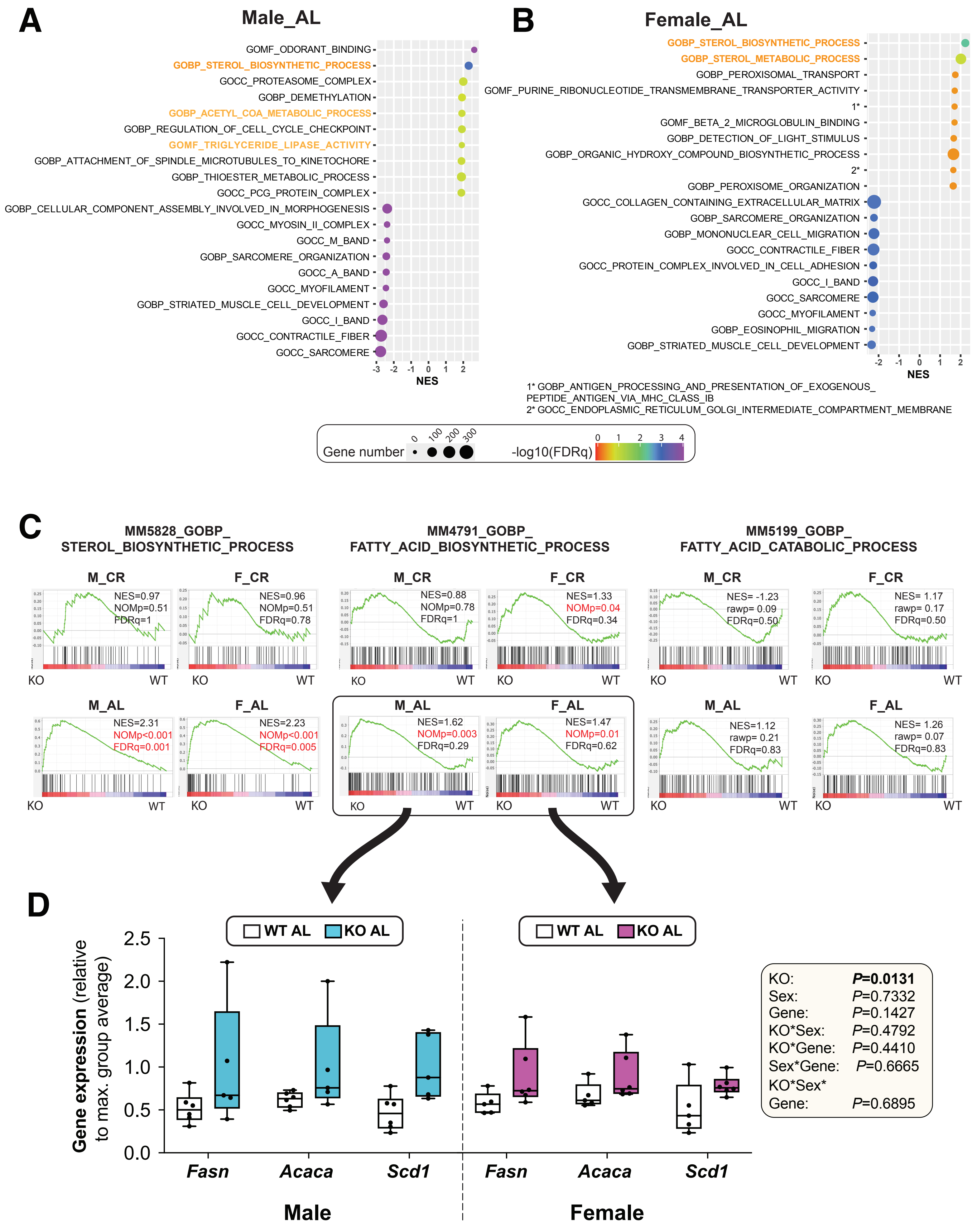

Ikushima et al, Metabolic functions of Adiponectin during caloric restriction, PLoS Biology, 2026

S7 Fig. Adiponectin KO alters lipid metabolism-related genes in AL but not CR mice. Male and Female WT and *Adipoq* KO mice were fed AL or CR and their livers analysed by bulk RNA-seq, as described for Fig 5. **(A-B)** Bubble plots of the top 10 GO gene sets (with lowest FDRq values) enriched in KO (NES>0) or WT (NES<0) within AL Male (A) or AL Female (B) subgroups. The GO terms are sorted by the NES values. Gene set names related to sterol metabolism are highlighted in orange, lipid catabolism in yellow **(C)** GSEA result between KO vs WT in AL Male (upper left), AL Female (upper right), CR Male (lower left), or CR Female (lower right) subgroups for MM5828_GOBP_STEROL_BIOSYNTHETIC_PROCESS (left), MM4791_GOBP_FATTY_ACID_BIOSYNTHETIC_PROCESS (middle), and MM5199_GOBP_FATTY_ACID_CATABOLIC_PROCESS (right). For (A-C), data were extracted from GSEA results conducted with the M5 gene set library (m5.all.v2023.2.Mm.symbols.gmt). For (A-B) bubble size and colour coding for FDRq values are shown below the bubble plots. In cases where the FDR-q value is 0, a value of 0.0001 has been used (-log10(FDRq) = 4) to allow visualisation. **(D)** Expression of key genes in the MM4791_GOBP_FATTY_ACID_BIOSYNTHETIC_PROCESS gene set (*Fasn, Acaca* and *Scd1*) based on normalised count data from liver RNAseq. To allow comparison across genes, the normalised counts are shown relative to the sex-genotype subgroup with the highest expression of each gene. Sample numbers for males and females are as described for Fig 5. Significant effects of genotype, sex and gene, and interactions thereof, were determined by 3-way ANOVA. The underlying data for this figure can be found in the S1_Data file.
