# Supplementary material for: Adiponectin exerts sex-dependent effects on lipid, amino acid, and glucose metabolism during caloric restriction
Source: PLoS Biol. 2026 Jun 18;24(6):e3003821. doi: 10.1371/journal.pbio.3003821 (PMC13278438; doi:10.1371/journal.pbio.3003821)
Supplement: S8 Fig — Male and Female WT and Adipoq KO mice were fed AL or CR and their livers analyzed by bulk RNA-seq, as described for Fig 5. (A, B) Bubble plots of the top 10 GO gene sets (with lowest FDRq values) enriched in KO (NES > 0) or WT (NES < 0) within CR Male (D) or CR Female (E) subgroups. The GO terms are sorted by the NES values. Gene set names related to amino acid metabolism are highlighted in pink. Data were extracted from GSEA results conducted with the M5 gene set library (m5.all.v2023.2.Mm.symbols.gmt). For (A–B) and (D–E), bubble size and color coding for FDRq values are shown below the bubble plots. In cases where the FDR-q value is 0, a value of 0.0001 has been used (−log10(FDRq) = 4) to allow visualization. (C,D) Clustered heat map of the genes that showed core enrichment in MM10709_GOBP_ALPHA_AMINO_ACID_CATABOLIC_PROCESS gene set in comparisons between KO vs. WT for CR Male (A) or CR Female (B) subgroups. The common genes between (C) and (D) are shown in bold. Sample numbers for liver RNA-seq are as follows: Male WT AL, n = 6; Male WT CR, n = 5; Male KO AL, n = 5; Male KO CR, n = 5; Female WT AL, n = 5; Female WT CR, n = 6; Female KO AL, n = 6; Female KO CR, n = 6. The underlying data for this figure can be found in the S1 Data file. (PDF) [file pbio.3003821.s008.pdf]

S8 Figure

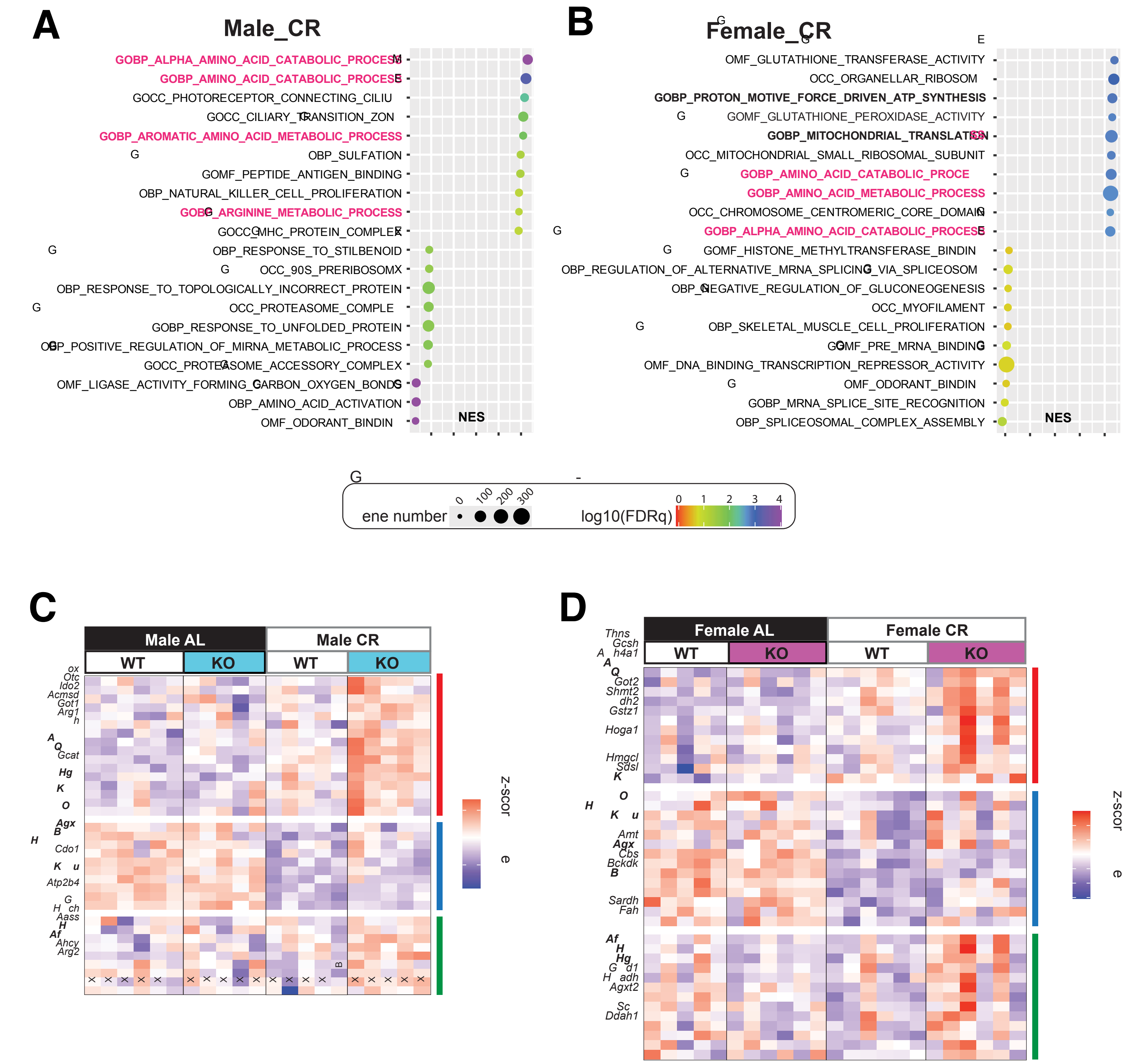

A
Male_CR
GOBP_ALPHA_AMINO_ACID_CATABOLIC_PROCESS
GOBP_AMINO_ACID_CATABOLIC_PROCESS
GOCC_PHOTORECEPTOR_CONNECTING_CILIU
GOCC_CILIARY_TRANSITION_ZON
GOBP_AROMATIC_AMINO_ACID_METABOLIC_PROCESS
OBP_SULFATION
GOMF_PEPTIDE_ANTIGEN_BINDING
OBP_NATURAL_KILLER_CELL_PROLIFERATION
GOBP_ARGININE_METABOLIC_PROCESS
GOCC_MHC_PROTEIN_COMPLEX
OBP_RESPONSE_TO_STILBENOID
OCC_90S_PRERIBOSOM
OBP_RESPONSE_TO_TOPOLOGICALLY_INCORRECT_PROTEIN
OCC_PROTEASOME_COMPLE
GOBP_RESPONSE_TO_UNFOLDED_PROTEIN
OBP_POSITIVE_REGULATION_OF_MIRNA_METABOLIC_PROCESS
GOCC_PROTEASOME_ACCESSORY_COMPLEX
OMF_LIGASE_ACTIVITY_FORMING_CARBON_OXYGEN_BONDS
OBP_AMINO_ACID_ACTIVATION
OMF_ODORANT_BINDIN
NES
B
Female_CR
OMF_GLUTATHIONE_TRANSFERASE_ACTIVITY
OCC_ORGANELLAR_RIBOSOM
GOBP_PROTON_MOTIVE_FORCE_DRIVEN_ATP_SYNTHESIS
GOMF_GLUTATHIONE_PEROXIDASE_ACTIVITY
GOBP_MITOCHONDRIAL_TRANSLATION
OCC_MITOCHONDRIAL_SMALL_RIBOSOMAL_SUBUNIT
GOBP_AMINO_ACID_CATABOLIC_PROCE
GOBP_AMINO_ACID_METABOLIC_PROCESS
OCC_CHROMOSOME_CENTROMERIC_CORE_DOMAIN
GOBP_ALPHA_AMINO_ACID_CATABOLIC_PROCESS
GOMF_HISTONE_METHYLTRANSFERASE_BINDIN
OBP_REGULATION_OF_ALTERNATIVE_MRNA_SPLICING_VIA_SPLICEOSOM
OBP_NEGATIVE_REGULATION_OF_GLUCONEOGENESIS
OCC_MYOFILAMENT
OBP_SKELETAL_MUSCLE_CELL_PROLIFERATION
GOMF_PRE_MRNA_BINDING
OMF_DNA_BINDING_TRANSCRIPTION_REPRESSOR_ACTIVITY
OMF_ODORANT_BINDIN
GOBP_MRNA_SPLICE_SITE_RECOGNITION
OBP_SPLICEOSOMAL_COMPLEX_ASSEMBLY
NES
ene number
0 100 200 300
log10(FDRq)
0 1 2 3 4
C
Male AL
Male CR
WT
KO
Otc
Ido2
Acmsd
Got1
Arg1
Gcat
Hg
Cdo1
Atp2b4
Aass
Ahcy
Arg2
Z-scor
D
Female AL
Female CR
WT
KO
Thns
Gcsh
Got2
Shmt2
Gstz1
Hoga1
Hmgcl
Sdsl
Amt
Agx
Cbs
Bckdk
Sardh
Fah
Agxt2
Ddah1
Z-scor

S8 Fig. Adiponectin KO leads to upregulated expression of genes related to amino acid catabolism under CR. Male and Female WT and *Adipoq* KO mice were fed AL or CR and their livers analysed by bulk RNA-seq, as described for Fig 5. **(A, B)** Bubble plots of the top 10 GO gene sets (with lowest FDRq values) enriched in KO (NES>0) or WT (NES<0) within CR Male (D) or CR Female (E) subgroups. The GO terms are sorted by the NES values. Gene set names related to amino acid metabolism are highlighted in pink. Data were extracted from GSEA results conducted with the M5 gene set library (m5.all.v2023.2.Mm.symbols.gmt). For (A-B) and (D-E), bubble size and colour coding for FDRq values are shown below the bubble plots. In cases where the FDR-q value is 0, a value of 0.0001 has been used (-log10(FDRq) = 4) to allow visualisation. **(C,D)** Clustered heat map of the genes that showed core enrichment in MM10709_GOBP_ALPHA_AMINO_ACID_CATABOLIC_PROCESS gene set in comparisons between KO vs WT for CR Male (A) or CR Female (B) subgroups. The common genes between (C) and (D) are shown in bold. Sample numbers for liver RNA-seq are as follows: *Male WT AL*, n=6; *Male WT CR,* n=5; *Male KO AL*, n=5; *Male KO CR*, n=5; *Female WT AL*, n=5; *Female WT CR*, n=6; *Female KO AL*, n=6; *Female KO CR*, n=6. The underlying data for this figure can be found in the S1_Data file.
