# Supplementary material for: Adiponectin exerts sex-dependent effects on lipid, amino acid, and glucose metabolism during caloric restriction
Source: PLoS Biol. 2026 Jun 18;24(6):e3003821. doi: 10.1371/journal.pbio.3003821 (PMC13278438; doi:10.1371/journal.pbio.3003821)
Supplement: S9 Fig — Male and female WT and Adipoq KO mice were fed AL or CR as described for Fig 1. At 13 weeks of age, mice were culled, and SkM samples were collected. (A–D) Gastrocnemius and soleus masses were recorded at necropsy (13 weeks of age) and are shown as absolute mass (A and B) or % body mass (C and D). Data areown as box-and-whisker plots of the following numbers of mice per group male WT AL, n = 26; male WT CR, n = 28; male KO AL, n = 21; male KO CR, n = 23 for gastrocnemius or 22 for soleus; female WT AL, n = 31 for gastrocnemius or 30 for soleus; female WT CR, n = 30 for gastrocnemius or 29 for soleus; female KO AL, n = 22 for gastrocnemius or 23 for soleus; female KO CR, n = 19 for gastrocnemius or 18 for soleus. Statistical analyses were as described for Fig 1E. The underlying data for this figure can be found in the S1 Data file. (PDF) [file pbio.3003821.s009.pdf]

S9 Figure

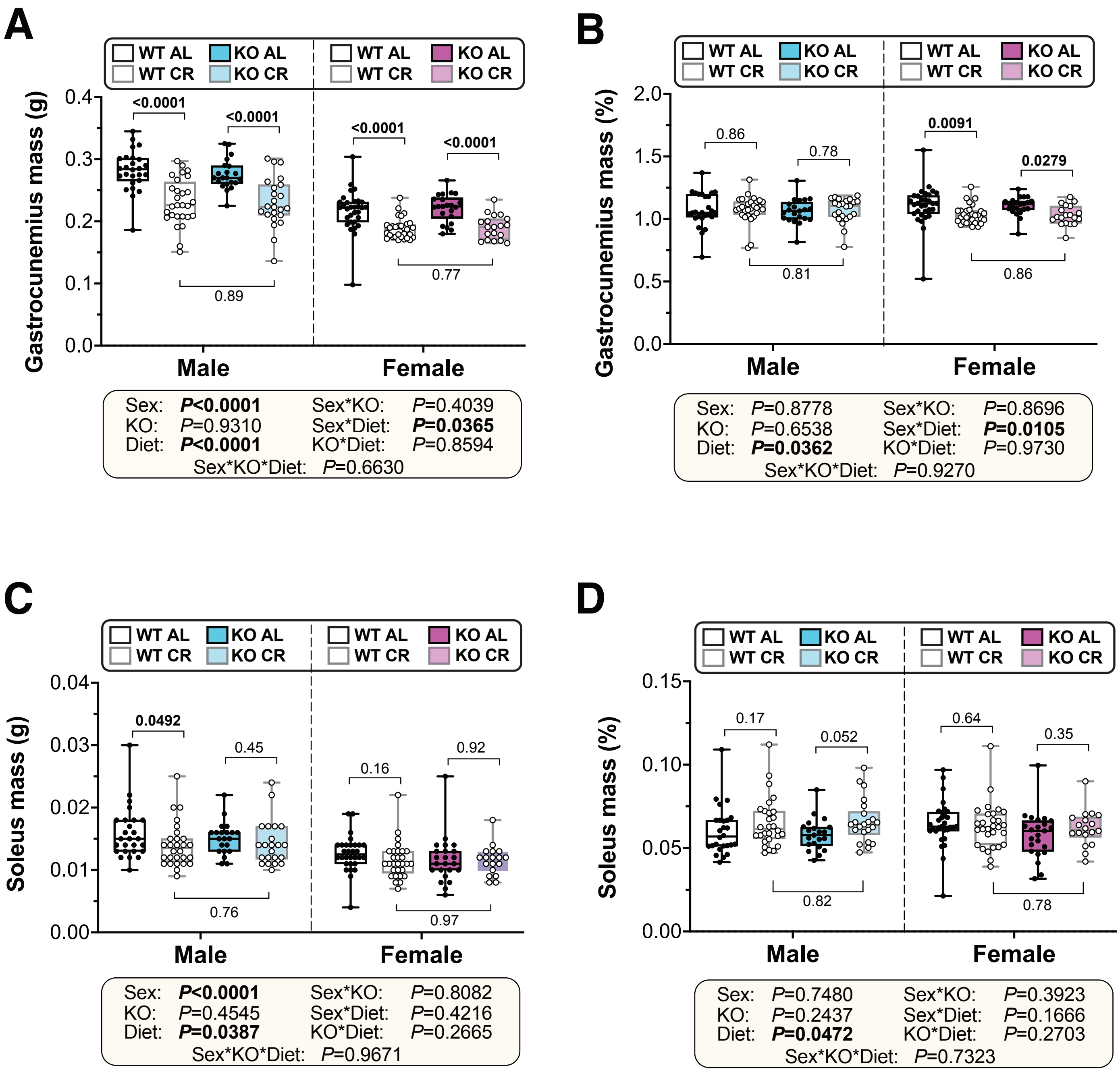

S9 Fig. Adiponectin KO does not alter muscle mass under AL nor CR diet. Male and female WT and *Adipoq* KO mice were fed AL or CR as described for Fig 1. At 13 weeks of age, mice were culled, and SkM samples were collected. **(A-D)** Gastrocnemius and soleus masses were recorded at necropsy (13 weeks of age) and are shown as absolute mass (A and B) or % body mass (C and D). Data areown as box-and-whisker plots of the following numbers of mice per group *male WT AL*, n=26 ; *male WT CR,* n=28 ; *male KO AL,* n=21; *male KO CR,* n=23 for gastrocnemius or 22 for soleus; *female WT AL*, n=31 for gastrocnemius or 30 for soleus; *female WT CR,* n=30 for gastrocnemius or 29 for soleus; *female KO AL,* n=22 for gastrocnemius or 23 for soleus; *female KO CR,* n=19 for gastrocnemius or 18 for soleus. Statistical analyses were as described for Fig 1E. The underlying data for this figure can be found in the S1_Data file.
