# Supplementary material for: Adiponectin exerts sex-dependent effects on lipid, amino acid, and glucose metabolism during caloric restriction
Source: PLoS Biol. 2026 Jun 18;24(6):e3003821. doi: 10.1371/journal.pbio.3003821 (PMC13278438; doi:10.1371/journal.pbio.3003821)
Supplement: S10 Fig — Male and female WT and Adipoq KO mice were fed AL or CR as described for Fig 1. At 13 weeks of age, mice were culled, and SkM samples were collected. (A) CSA of gastrocnemius mass at the cull. (B) Representative images of gastrocnemius cross-sectional sections. Scale bars indicate 100 μm. Data are shown as box-and-whisker plots of the following numbers of mice per group male WT AL, n = 5; male WT CR, n = 6; male KO AL, n = 4; male KO CR, n = 6; female WT AL, n = 6; female WT CR, n = 6; female KO AL, n = 5; female KO CR, n = 5. Statistical analyses were as described for Fig 1E. The underlying data for this figure can be found in the S1 Data file. (PDF) [file pbio.3003821.s010.pdf]

S10 Figure

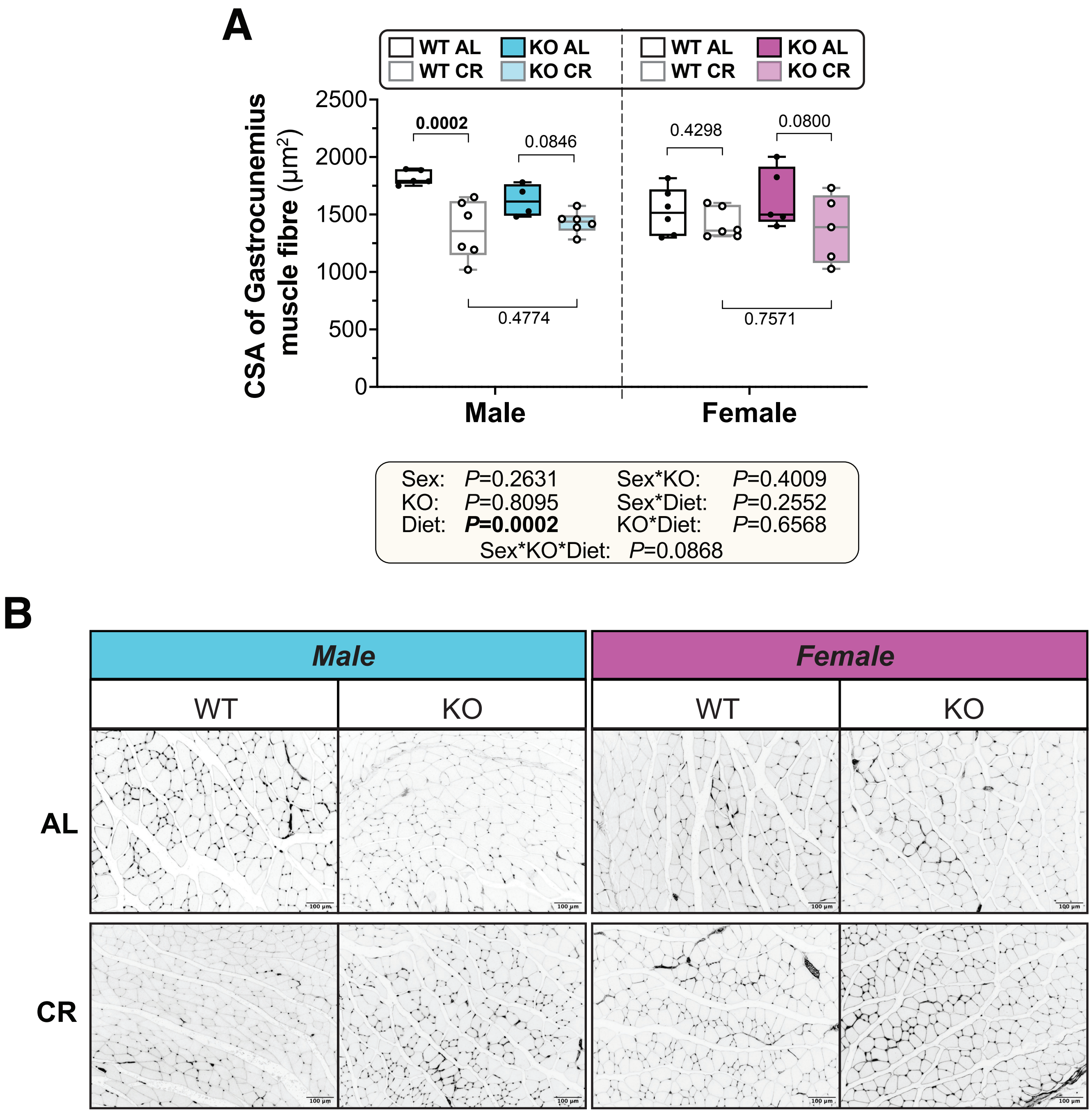

S10 Fig. Adiponectin KO does not alter muscle fibre CSA in gastrocnemius under AL nor CR diet. Male and female WT and *Adipoq* KO mice were fed AL or CR as described for Fig 1. At 13 weeks of age, mice were culled, and SkM samples were collected. **(A)** CSA of gastrocnemius mass at the cull. **(B)** Representative images of gastrocnemius cross-sectional sections. Scale bars indicate 100um. Data are shown as box-and-whisker plots of the following numbers of mice per group *male WT AL*, n=5; *male WT CR,* n=6 ; *male KO AL,* n=4; *male KO CR,* n=6 ; *female WT AL*, n=6 ; *female WT CR,* n=6; *female KO AL,* n=5; *female KO CR,* n=5. Statistical analyses were as described for Fig 1E. The underlying data for this figure can be found in the S1_Data file.
