# Supplementary material for: Adiponectin exerts sex-dependent effects on lipid, amino acid, and glucose metabolism during caloric restriction
Source: PLoS Biol. 2026 Jun 18;24(6):e3003821. doi: 10.1371/journal.pbio.3003821 (PMC13278438; doi:10.1371/journal.pbio.3003821)
Supplement: S11 Fig — Male and female WT and Adipoq KO mice were fed AL or CR and their livers analyzed by bulk RNA-seq, as described for Fig 5. (A–E) Normalized count data for Gpd1 (A), Gpd2 (B), Got1 (C), Sds (D), and Fbp2 (E); for space reasons, the y-axes for (C) and (D) show counts in thousands (k). Sample numbers for males and females are as described for Fig 5. Statistical analyses are as described for Fig 1E. (F–G) Glycerol tolerance tests in male (F) and female (G) mice, shown as mean ± SEM of the following numbers of mice per group: males, n = 14 (WT AL, WT CR), 10 (KO AL), or 11 (KO CR); females, n = 16 (WT AL), 14 (WT CR), 10 (KO AL), or 7 (KO CR). Significant effects of diet, time, and/or genotype, and interactions thereof, were determined by 3-way ANOVA. Within each diet and sex, significant genotype effects at each time point were determined by 2-way ANOVA with Šidák’s multiple comparisons test; ** (P < 0.01). The underlying data for this figure can be found in the S1 Data file. (PDF) [file pbio.3003821.s011.pdf]

S11 Figure

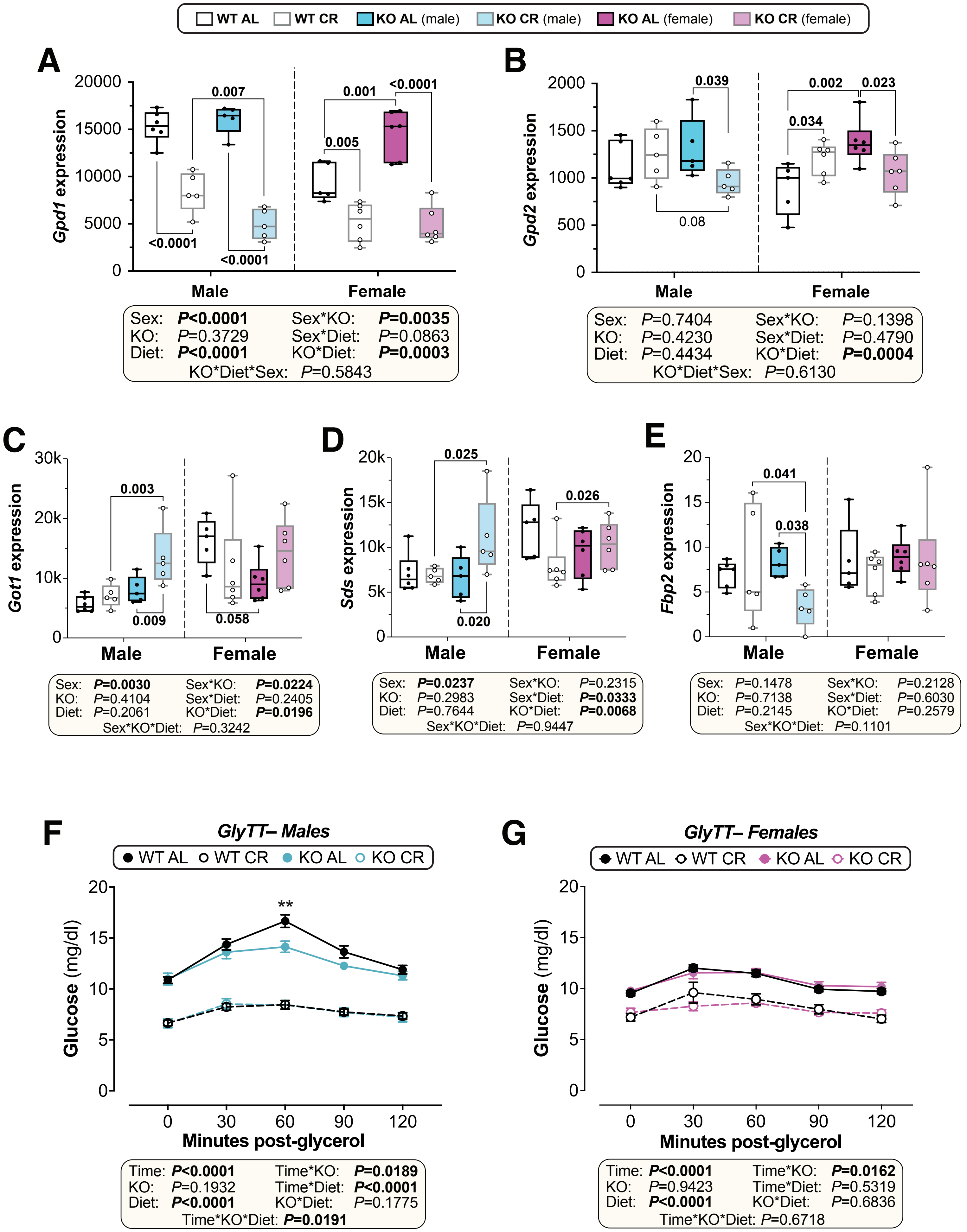

WT AL
WT CR
KO AL (male)
KO CR (male)
KO AL (female)
KO CR (female)
A
Gpd1 expression
20000
15000
10000
5000
0
0.007
<0.0001
<0.0001
0.001
<0.0001
0.005
Male
Female
Sex: P<0.0001
KO: P=0.3729
Diet: P<0.0001
Sex*KO: P=0.0035
Sex*Diet: P=0.0863
KO*Diet: P=0.0003
KO*Diet*Sex: P=0.5843
B
Gpd2 expression
2000
1500
1000
500
0
0.039
0.08
0.002
0.023
0.034
Male
Female
Sex: P=0.7404
KO: P=0.4230
Diet: P=0.4434
Sex*KO: P=0.1398
Sex*Diet: P=0.4790
KO*Diet: P=0.0004
KO*Diet*Sex: P=0.6130
C
Got1 expression
30k
20k
10k
0
0.003
0.009
0.058
Male
Female
Sex: P=0.0030
KO: P=0.4104
Diet: P=0.2061
Sex*KO: P=0.0224
Sex*Diet: P=0.2405
KO*Diet: P=0.0196
Sex*KO*Diet: P=0.3242
D
Sds expression
20k
15k
10k
5k
0
0.025
0.020
0.026
Male
Female
Sex: P=0.0237
KO: P=0.2983
Diet: P=0.7644
Sex*KO: P=0.2315
Sex*Diet: P=0.0333
KO*Diet: P=0.0068
Sex*KO*Diet: P=0.9447
E
Fbp2 expression
20
15
10
5
0
0.041
0.038
Male
Female
Sex: P=0.1478
KO: P=0.7138
Diet: P=0.2145
Sex*KO: P=0.2128
Sex*Diet: P=0.6030
KO*Diet: P=0.2579
Sex*KO*Diet: P=0.1101
F
GlyTT– Males
WT AL
WT CR
KO AL
KO CR
Glucose (mg/dl)
20
15
10
5
0
**
0
30
60
90
120
Minutes post-glycerol
Time: P<0.0001
KO: P=0.1932
Diet: P<0.0001
Time*KO: P=0.0189
Time*Diet: P<0.0001
KO*Diet: P=0.1775
Time*KO*Diet: P=0.0191
G
GlyTT– Females
WT AL
WT CR
KO AL
KO CR
Glucose (mg/dl)
20
15
10
5
0
0
30
60
90
120
Minutes post-glycerol
Time: P<0.0001
KO: P=0.9423
Diet: P<0.0001
Time*KO: P=0.0162
Time*Diet: P=0.5319
KO*Diet: P=0.6836
Time*KO*Diet: P=0.6718

S11 Fig. Effects of adiponectin KO on hepatic expression of gluconeogenesis-related genes and on glycerol-driven gluconeogenesis. Male and female WT and *Adipoq* KO mice were fed AL or CR and their livers analysed by bulk RNA-seq, as described for Fig 5. **(A-E)** Normalised count data for *Gpd1* (A), *Gpd2* (B), *Got1* (C), *Sds* (D) and *Fbp2* (E); for space reasons, the y-axes for (C) and (D) show counts in thousands (k). Sample numbers for males and females are as described for Fig 5. Statistical analyses are as described for Fig 1E. **(F-G)** Glycerol tolerance tests in male (F) and female (G) mice, shown as mean ± SEM of the following numbers of mice per group: *males*, n= 14 (WT AL, WT CR), 10 (KO AL), or 11 (KO CR); *females*, n= 16 (WT AL), 14 (WT CR), 10 (KO AL), or 7 (KO CR). Significant effects of diet, time, and/or genotype, and interactions thereof, were determined by 3-way ANOVA. Within each diet and sex, significant genotype effects at each time point were determined by 2-way ANOVA with Šidák's multiple comparisons test; ** ($P<0.01$). The underlying data for this figure can be found in the S1_Data file.
