# Supplementary material for: Adiponectin exerts sex-dependent effects on lipid, amino acid, and glucose metabolism during caloric restriction
Source: PLoS Biol. 2026 Jun 18;24(6):e3003821. doi: 10.1371/journal.pbio.3003821 (PMC13278438; doi:10.1371/journal.pbio.3003821)
Supplement: S1 Table — The table shows LC–MS-measured concentrations of total and each species of ceramide. Average ± SEM are shown. Outlier identification analysis was conducted with the Rout method (Q = 1%) and three outliers were excluded from the data for Ceramide 26:0. The underlying data for this table can be found in the S1 Data file. (PDF) [file pbio.3003821.s012.pdf]

	**Ceramide concentrations** (pmol/100 mg tissue)				***P* (2-way ANOVA)**			**Number per group for Ceramide analysis**			
	**Male AL WT**	**Male AL KO**	**Male CR WT**	**Male CR KO**	**Genotype*Diet**	**Genotype**	**Diet**	**Male AL WT**	**Male AL KO**	**Male CR WT**	**Male CR KO**
**14:0**	19.26 ± 3.17	16.88 ± 5.66	46.19 ± 5.65	32.8 ± 4.9	0.2869	0.131	**0.0002**	9	5	9	9
**16:0**	838.28 ± 87.98	657.79 ± 77.39	1306.29 ± 93.49	1357.7 ± 77.7	0.2133	0.4843	**<0.0001**	9	5	9	9
**18:0**	223.44 ± 26.03	135.6 ± 19.8	454.84 ± 36.29	441.04 ± 51.55	0.3673	0.2188	**<0.0001**	9	5	9	9
**18:1**	4.86 ± 0.89	4.28 ± 1.45	10.54 ± 1.37	9.5 ± 0.93	0.8469	0.4986	**<0.0001**	9	5	9	9
**20:0**	992.16 ± 207.89	876.97 ± 224.07	695.78 ± 149.61	572.94 ± 115.92	0.9831	0.5105	0.1038	9	5	9	9
**20:1**	10.47 ± 1.65	12.65 ± 3.42	21.81 ± 2.05	16.68 ± 2.98	0.1636	0.5682	**0.0055**	9	5	9	9
**22:0**	3321.9 ± 321.42	3625.44 ± 258.71	1766.43 ± 141.47	1583.24 ± 139.29	0.3096	0.7999	**<0.0001**	9	5	9	9
**22:1**	575.89 ± 74.23	745.27 ± 144.54	252.81 ± 34.23	172.13 ± 22.2	0.0714	0.5116	**<0.0001**	9	5	9	9
**23:0**	903.84 ± 63.76	882.09 ± 68.95	1426.78 ± 99.53	1413.06 ± 79	0.963	0.8379	**<0.0001**	9	5	9	9
**23:1**	53.23 ± 3.64	62.87 ± 7.61	66.34 ± 4.13	52.12 ± 4.76	**0.0207**	0.6413	0.8095	9	5	9	9
**24:0**	2374.85 ± 126.99	2400.7 ± 142.03	2423.81 ± 124.37	2385.37 ± 111.35	0.8071	0.9619	0.8983	9	5	9	9
**24:1**	2891.56 ± 234.71	2753.28 ± 272.46	2649.42 ± 199.14	2613.44 ± 321.52	0.8532	0.7527	0.4913	9	5	9	9
**26:0**	8.58 ± 1.02	8.82 ± 1.14	18.51 ± 1.15	19.81 ± 1.73	0.705	0.5843	**<0.0001**	7	5	9	8
**26:1**	13.55 ± 2.69	8.61 ± 1.3	24.61 ± 1.83	20.89 ± 1.6	0.7787	0.0549	**<0.0001**	9	5	9	9
**Total**	12270.12 ± 775.84	12191.23 ± 996.31	11164.16 ± 559.52	10699.35 ± 722.81	0.8031	0.7255	0.1012	9	5	9	9

S1 Table. Ceramide concentrations for different sphingolipid species.
The table shows LC-MS-measured concentrations of total and each species of ceramide. Average ± SEM are shown. Outlier identification analysis was conducted with the Rout method (Q=1%) and three outliers were excluded from the data for Ceramide 26:0. The underlying data for this table can be found in the S1_Data file.
