# Supplementary material for: Adiponectin exerts sex-dependent effects on lipid, amino acid, and glucose metabolism during caloric restriction
Source: PLoS Biol. 2026 Jun 18;24(6):e3003821. doi: 10.1371/journal.pbio.3003821 (PMC13278438; doi:10.1371/journal.pbio.3003821)
Supplement: S2 Table — The table shows LC–MS-measured concentrations of total and each species of DHC. Average ± SEM are shown. DHC 26:0 was not detected for 7 samples and DHC 26:1 was not detected for 1 sample. The cells for those values were left blank. The underlying data for this table can be found in the S1 Data file. (PDF) [file pbio.3003821.s013.pdf]

	DHC concentrations (pmol/100 mg tissue)				*P* (2-way ANOVA)			Number per group for DHC analysis			
	**Male AL WT**	**Male AL KO**	**Male CR WT**	**Male CR KO**	**Genotype*Diet**	**Genotype**	**Diet**	**Male AL WT**	**Male AL KO**	**Male CR WT**	**Male CR KO**
**14:0**	0.64 ± 0.22	0.51 ± 0.32	2.87 ± 1.04	3.4 ± 1.13	0.7226	0.83	**0.0091**	9	5	9	9
**16:0**	60.41 ± 7.26	47.3 ± 7.26	122.53 ± 16.08	132.28 ± 16.45	0.4311	0.9074	**<0.0001**	9	5	9	9
**18:0**	30.97 ± 7.7	18.24 ± 3.64	80.45 ± 10.94	93.79 ± 23.18	0.414	0.9846	**0.0004**	9	5	9	9
**18:1**	1.74 ± 0.47	1.19 ± 0.24	5.06 ± 1.05	4.94 ± 0.9	0.8025	0.6973	**0.0003**	9	5	9	9
**20:0**	52.11 ± 12.53	42.45 ± 3.36	58.57 ± 5.09	58.21 ± 11.99	0.6651	0.6408	0.3051	9	5	9	9
**20:1**	1.46 ± 0.61	1.61 ± 0.54	2.93 ± 0.5	3.61 ± 1.11	0.7433	0.6113	**0.0409**	9	5	9	9
**22:0**	179.2 ± 21.41	169.51 ± 18.83	111.89 ± 8.09	102.26 ± 15.3	0.9989	0.5775	**0.0005**	9	5	9	9
**22:1**	46.55 ± 7.42	56.23 ± 7.47	29.98 ± 3.35	24.11 ± 4.5	0.1995	0.7502	**0.0003**	9	5	9	9
**23:0**	44.12 ± 5.5	46.4 ± 6.76	108.17 ± 6.31	102.8 ± 16.27	0.7284	0.8884	**<0.0001**	9	5	9	9
**23:1**	ND	ND	ND	ND				0	0	0	0
**24:0**	103.03 ± 10.96	119.56 ± 14.06	163.76 ± 10.26	147.9 ± 17.77	0.2669	0.9814	**0.0042**	9	5	9	9
**24:1**	199.66 ± 13.52	189.28 ± 12.94	246.29 ± 29.73	213.46 ± 24.67	0.6476	0.3815	0.1561	9	5	9	9
**26:0**	0.45 ± 0.14	0.34 ± 0.19	0.24 ± 0.06	0.27 ± 0.05	0.5747	0.7487	0.2459	7	2	7	9
**26:1**	0.48 ± 0.12	0.95 ± 0.13	1.73 ± 0.39	1.35 ± 0.35	0.224	0.8971	**0.0208**	9	4	9	9
**Total**	303.52 ± 22.99	309.73 ± 26.94	411.96 ± 37.38	362.99 ± 41.44	0.4536	0.5607	**0.0342**	9	5	9	9

S2 Table. DHC concentrations for different sphingolipid species.
The table shows LC-MS-measured concentrations of total and each species of DHC. Average ± SEM are shown. DHC 26:0 was not detected for 7 samples and DHC 26:1 was not detected for 1 sample. The cells for those values were left blank. The underlying data for this table can be found in the S1_Data file.
