# Supplementary material for: Adiponectin exerts sex-dependent effects on lipid, amino acid, and glucose metabolism during caloric restriction
Source: PLoS Biol. 2026 Jun 18;24(6):e3003821. doi: 10.1371/journal.pbio.3003821 (PMC13278438; doi:10.1371/journal.pbio.3003821)
Supplement: S3 Table — The table shows Ceramide:DHC ratio based on LC–MS-measured concentrations of total and each species of ceramide (S1 Table) and DHC (S2 Table). Average ± SEM are shown. Outlier identification analysis was conducted with the Rout method (Q = 1%) and three outliers were excluded from the data for Ceramide 26:0. The Ceramide:DHC ratio data for these three samples were also excluded. DHC 26:0 was not detected for 7 samples and DHC 26:1 was not detected for 1 sample, and the corresponding ceramide:DHC ratio values were left as blank. The underlying data for this table can be found in the S1 Data file. (PDF) [file pbio.3003821.s014.pdf]

	Ceramide:DHC ratio				*P* (2-way ANOVA)			Number per group for Ceramide:DHC ratio			
	Male AL WT	Male AL KO	Male CR WT	Male CR KO	Genotype*Diet	Genotype	Diet	Male AL WT	Male AL KO	Male CR WT	Male CR KO
**14:0**	68.16 ± 22.08	110.07 ± 41.48	47.55 ± 18.94	30.1 ± 13.88	0.2037	0.5962	**0.0358**	9	5	9	9
**16:0**	14.25 ± 0.93	14.15 ± 0.5	11.48 ± 0.99	11.32 ± 1.2	0.9785	0.9051	**0.0143**	9	5	9	9
**18:0**	8.89 ± 0.95	7.74 ± 0.57	6.2 ± 0.6	7.33 ± 1.66	0.3443	0.9937	0.2019	9	5	9	9
**18:1**	4.87 ± 2.24	4.2 ± 1.13	2.31 ± 0.2	2.32 ± 0.35	0.807	0.8153	0.1183	9	5	9	9
**20:0**	22.58 ± 3.95	19.83 ± 3.6	12.18 ± 2.42	14.74 ± 4.76	0.5172	0.9803	0.0659	9	5	9	9
**20:1**	22.35 ± 7.78	10.25 ± 3.75	9.12 ± 1.48	7.8 ± 1.54	0.2773	0.1785	0.118	9	5	9	9
**22:0**	20.23 ± 2.16	22.01 ± 1.88	16.05 ± 1.31	20.16 ± 4.7	0.7163	0.3627	0.3507	9	5	9	9
**22:1**	13.76 ± 1.63	12.92 ± 1.1	8.94 ± 1.21	11.05 ± 3.64	0.5546	0.7991	0.1849	9	5	9	9
**23:0**	22.37 ± 2.46	20.91 ± 3.93	13.66 ± 1.47	17.55 ± 3.43	0.3622	0.676	**0.0454**	9	5	9	9
**23:1**								0	0	0	0
**24:0**	24.54 ± 2.31	21.15 ± 2.37	15.24 ± 1.25	18.52 ± 2.79	0.168	0.9813	**0.0173**	9	5	9	9
**24:1**	15.05 ± 1.66	14.52 ± 0.88	11.75 ± 1.34	15.29 ± 3.82	0.4364	0.5645	0.6274	9	5	9	9
**26:0**	49.13 ± 12.55	31.53 ± 16.3	93.4 ± 14.95	97.45 ± 18.84	0.6132	0.7512	**0.0174**	5	2	7	8
**26:1**	43.61 ± 10.49	11.01 ± 2.66	19.67 ± 3.98	27.85 ± 8.8	**0.0304**	0.1824	0.6935	9	4	9	9
**Total**	41.58 ± 2.93	40.05 ± 3.1	28.9 ± 3.1	34.54 ± 6.37	0.4412	0.6574	0.0574	9	5	9	9

S3 Table. Ceramide:DHC ratio for different sphingolipid species.
The table shows Ceramide:DHC ratio based on LC-MS-measured concentrations of total and each species of ceramide (Supplementary Table1) and DHC (Supplementary Table 2). Average ± SEM are shown. Outlier identification analysis was conducted with the Rout method (Q=1%) and three outliers were excluded from the data for Ceramide 26:0. The Ceramide:DHC ratio data for these three samples were also excluded. DHC 26:0 was not detected for 7 samples and DHC 26:1 was not detected for 1 sample, and the correnponding ceramide:DHC ratio values were left as blank. The underlying data for this table can be found in the S1_Data file.
