# Supplementary material for: Adiponectin exerts sex-dependent effects on lipid, amino acid, and glucose metabolism during caloric restriction
Source: PLoS Biol. 2026 Jun 18;24(6):e3003821. doi: 10.1371/journal.pbio.3003821 (PMC13278438; doi:10.1371/journal.pbio.3003821)
Supplement: S4 Table — The list of genes not included in the volcano plots in Fig 5B because the absolute log2FoldChange is over 10 or −log10(pvalue) is over 7.5. For CR/Females no genes meet these criteria (“n/a/”). The underlying data for this table can be found in the S1 Data file, in the sheets relating to Fig 5B. (PDF) [file pbio.3003821.s015.pdf]

Diet/Sex	geneID	baseMean	log2 Fold-Change	lfcSE	stat	pvalue	padj
AL/Male	*3425401B19Rik*	5.81	-19.2	2.50	-7.65	1.97E-14	3.38E-10
	Glycam1	10.96	-32.4	4.48	-7.21	5.39E-13	4.61E-09
CR/Male	*Myl2*	9.19	-19.7	2.65	-7.43	1.11E-13	1.90E-09
	Akp3	8.16	15.8	2.66	5.93	3.08E-09	2.63E-05
AL/Female	*Glycam1*	10.96	-20.3	4.39	-4.63	3.69E-06	0.052
CR/Female	n/a	n/a	n/a	n/a	n/a	n/a	n/a

S4 Table. Genes not included in the volcano plots in Figure 5B. The list of genes not **included** in the volcano plots in Figure 5B because the absolute log2FoldChange is over 10 or -log10(pvalue) is over 7.5. For CR/Females no genes meet these criteria (“n/a/”). The underlying data for this table can be found in the S1_Data file, in the sheets relating to Figure 5B.
