# Supplementary material for: Adiponectin exerts sex-dependent effects on lipid, amino acid, and glucose metabolism during caloric restriction
Source: PLoS Biol. 2026 Jun 18;24(6):e3003821. doi: 10.1371/journal.pbio.3003821 (PMC13278438; doi:10.1371/journal.pbio.3003821)
Supplement: S5 Table — Hepatic amino acid concentrations (ng/g) were measured by LC–MS/MS. Data for each group (sex/diet/genotype) are shown as average ± SEM. P values from 3-way ANOVA are shown for effects of each independent variable and their interactions. Asp, Gln, and Tyr were not detectable. (PDF) [file pbio.3003821.s016.pdf]

| Amino acid (ng/g) | Male AL WT | Male CR WT | Male AL KO | Male CR KO | Female AL WT | Female CR WT | Female AL KO | Female CR KO | Sex | Genotype | Diet | Sex x Genotype | Sex x Diet | Genotype x Diet | Sex x Genotype x Diet |
|---|---|---|---|---|---|---|---|---|---|---|---|---|---|---|---|
| **Glu** | 116.9 ± 8.5 | 162.5 ± 7.8 | 102.6 ± 5.2 | 173.2 ± 23.2 | 189.9 ± 12.8 | 184.5 ± 18.5 | 143.9 ± 12 | 197.6 ± 36.6 | **0.004** | 0.493 | **0.003** | 0.582 | 0.205 | 0.118 | 0.521 |
| **Gly** | 287.3 ± 17.7 | 327.3 ± 20.7 | 277.6 ± 20.6 | 340.7 ± 17.4 | 287.1 ± 14 | 294.9 ± 22.6 | 242.8 ± 12 | 353.1 ± 38.2 | 0.373 | 0.775 | **0.001** | 0.866 | 0.808 | **0.047** | 0.201 |
| **Ala** | 567.9 ± 41.3 | 526 ± 33.3 | 529.6 ± 37 | 499.9 ± 35.2 | 489.7 ± 27.9 | 482 ± 62.2 | 488.6 ± 29.7 | 457.1 ± 21.2 | 0.060 | 0.402 | 0.305 | 0.720 | 0.763 | 0.914 | 0.736 |
| **Ser** | 74.9 ± 15.9 | 68.8 ± 24 | 60.5 ± 9.2 | 55.3 ± 8.4 | 55.4 ± 7.2 | 45.6 ± 3.7 | 55.8 ± 7.4 | 42.8 ± 4.1 | 0.078 | 0.366 | 0.311 | 0.449 | 0.729 | 0.942 | 0.901 |
| **Pro** | 26 ± 4.7 | 23.7 ± 3.5 | 22.4 ± 3.3 | 25.2 ± 1.7 | 24.7 ± 1.7 | 26.5 ± 2.8 | 24.1 ± 3.6 | 25 ± 3.7 | 0.735 | 0.647 | 0.737 | 0.998 | 0.823 | 0.654 | 0.519 |
| **Val** | 60.1 ± 10.2 | 57.2 ± 9.9 | 50 ± 7.6 | 60.7 ± 3.9 | 53.1 ± 2.7 | 56.9 ± 5.2 | 53.1 ± 6.6 | 55.1 ± 9 | 0.634 | 0.682 | 0.512 | 0.819 | 0.920 | 0.566 | 0.458 |
| **Thr** | 55.3 ± 8.8 | 52.9 ± 14.1 | 47 ± 7.6 | 42.7 ± 4.7 | 48.8 ± 3 | 44.5 ± 3.9 | 48 ± 5 | 42.1 ± 5.2 | 0.487 | 0.300 | 0.417 | 0.456 | 0.863 | 0.866 | 0.989 |
| **Leu** | 103.2 ± 19.2 | 87.8 ± 20.1 | 83.5 ± 15.9 | 82.6 ± 6.1 | 81.9 ± 6 | 76.5 ± 4.8 | 77.7 ± 6.8 | 75.4 ± 11.3 | 0.206 | 0.399 | 0.500 | 0.584 | 0.808 | 0.625 | 0.750 |
| **Ile** | 42.1 ± 6.7 | 34.8 ± 5.2 | 33.2 ± 4.7 | 34.1 ± 2.1 | 33 ± 2.2 | 33.5 ± 2.6 | 32.9 ± 3 | 31.8 ± 4.3 | 0.266 | 0.330 | 0.552 | 0.506 | 0.625 | 0.571 | 0.404 |
| **Asp** | 40.4 ± 7.3 | 37.2 ± 7.5 | 27.3 ± 4.7 | 31.1 ± 5.8 | 36.1 ± 3.7 | 27.7 ± 3.6 | 36.2 ± 2.6 | 34.9 ± 3.1 | 0.938 | 0.417 | 0.534 | 0.076 | 0.474 | 0.338 | 0.996 |
| **Lys** | 62 ± 11.5 | 52 ± 10.1 | 49.5 ± 10.2 | 54.7 ± 5.5 | 66.5 ± 4 | 56.1 ± 6.5 | 58.7 ± 5.9 | 56.5 ± 7.5 | 0.394 | 0.445 | 0.444 | 0.917 | 0.733 | 0.306 | 0.758 |
| **Met** | 10.4 ± 2.6 | 7.7 ± 2.2 | 7.4 ± 1.8 | 7 ± 0.5 | 7.4 ± 0.7 | 6.8 ± 0.9 | 7.4 ± 1.6 | 7 ± 1.3 | 0.399 | 0.441 | 0.356 | 0.390 | 0.627 | 0.564 | 0.658 |
| **His** | 64.1 ± 7 | 58.9 ± 5.4 | 62.4 ± 7.2 | 58 ± 3.9 | 60.1 ± 3.9 | 55.6 ± 2.3 | 58.3 ± 2.3 | 56.7 ± 4.6 | 0.358 | 0.818 | 0.259 | 0.891 | 0.798 | 0.783 | 0.879 |
| **Phe** | 36.9 ± 5.7 | 32.1 ± 8 | 30 ± 5 | 28.8 ± 2.2 | 30.6 ± 2.1 | 27.7 ± 1.9 | 30 ± 2.4 | 26.8 ± 2.6 | 0.299 | 0.328 | 0.312 | 0.475 | 0.997 | 0.785 | 0.741 |
| **Arg** | 153.3 ± 28.7 | 123.1 ± 22.7 | 135.8 ± 21.8 | 111.1 ± 26.5 | 129 ± 20.7 | 118.2 ± 8.7 | 108.2 ± 12.6 | 133.5 ± 22.1 | 0.578 | 0.572 | 0.516 | 0.697 | 0.266 | 0.503 | 0.624 |
| **Trp** | 35.4 ± 8 | 31.2 ± 7.9 | 28.1 ± 5.6 | 29.8 ± 2.5 | 24.7 ± 2.1 | 25.3 ± 2.2 | 25.2 ± 3.2 | 26.3 ± 4.3 | 0.110 | 0.610 | 0.954 | 0.475 | 0.769 | 0.648 | 0.698 |
| **Cys** | 0.02 ± 0.02 | 0 ± 0 | 0.02 ± 0.02 | 0.01 ± 0.01 | 0 ± 0 | 0 ± 0 | 0.02 ± 0.02 | 0.02 ± 0.02 | 0.800 | 0.267 | 0.424 | 0.424 | 0.424 | 0.800 | 0.800 |
| **BCAA** | 205.3 ± 36 | 179.8 ± 34.7 | 166.7 ± 28.1 | 177.4 ± 11.5 | 168 ± 10.7 | 166.9 ± 11.4 | 163.7 ± 15.6 | 162.3 ± 24.5 | 0.307 | 0.454 | 0.794 | 0.631 | 0.854 | 0.589 | 0.584 |
| **BCAA** (% total) | 11.4 ± 1 | 10.3 ± 1 | 10.5 ± 0.8 | 10.8 ± 0.5 | 10.4 ± 0.3 | 10.7 ± 0.6 | 10.9 ± 0.4 | 9.8 ± 0.8 | 0.495 | 0.671 | 0.445 | 0.982 | 0.990 | 0.970 | 0.156 |
| **Number of mice** | 6 | 6 | 6 | 7 | 6 | 6 | 6 | 6 | | | | | | | |

**S5 Table. Amino acid concentrations in the liver**

Hepatic amino acid concentrations (ng/g) were measured by LC-MS/MS. Data for each group (sex/diet/genotype) are shown as average ± SEM. P values from 3-way ANOVA are shown for effects of each independent variable and their interactions. Asp, Gln, and Tyr were not detectable.
