# Supplementary material for: Adiponectin exerts sex-dependent effects on lipid, amino acid, and glucose metabolism during caloric restriction
Source: PLoS Biol. 2026 Jun 18;24(6):e3003821. doi: 10.1371/journal.pbio.3003821 (PMC13278438; doi:10.1371/journal.pbio.3003821)
Supplement: S6 Table — Plams amino acid concentrations (ng/g) were measured by LC–MS/MS. Data for each group (sex/diet/genotype) are shown as average ± SEM. P values from 3-way ANOVA are shown for effects of each independent variable and their interactions. Asp, Gln, and Tyr were not detectable. (PDF) [file pbio.3003821.s017.pdf]

**Amino acid** (ng/g)	**Male AL WT**	**Male CR WT**	**Male AL KO**	**Male CR KO**	**Female AL WT**	**Female CR WT**	**Female AL KO**	**Female CR KO**	**Sex**	**Genotype**	**Diet**	**Sex x Genotype**	**Sex x Diet**	**Genotype x Diet**	**Sex x Genotype x Diet**
**Glu**	17.1 ± 3.2	15 ± 3.5	24.6 ± 5.3	15 ± 2.1	12.8 ± 1.3	15.3 ± 1.6	14.4 ± 1.4	14.2 ± 2.4	**0.050**	0.285	0.221	0.366	0.069	0.184	0.529
**Gly**	45.4 ± 3.7	41 ± 2.5	47.4 ± 3.5	44.5 ± 2.8	39.5 ± 1.7	40.5 ± 1.4	37.1 ± 2.4	41.4 ± 2.4	**0.006**	0.501	0.676	0.273	0.069	0.599	0.714
**Ala**	58.8 ± 6	41.9 ± 6.3	68.9 ± 8.2	43 ± 4.1	41.8 ± 2	39.3 ± 5.1	44 ± 2.8	34.9 ± 2.6	**0.0002**	0.480	**0.0001**	0.300	**0.022**	0.235	0.817
**Ser**	24.5 ± 3.1	18 ± 2.7	30.1 ± 5.3	19.9 ± 2.8	18.3 ± 0.7	16.1 ± 1.3	17.5 ± 0.8	16.8 ± 1.7	**0.001**	0.309	**0.007**	0.288	0.054	0.751	0.479
**Pro**	7.3 ± 1.4	5.5 ± 1.4	10.9 ± 3	5.6 ± 1.1	4.9 ± 0.3	5.8 ± 1.7	5 ± 0.3	4.6 ± 0.4	**0.017**	0.471	0.081	0.200	0.047	0.176	0.556
**Val**	18.4 ± 1.5	14.8 ± 1.8	20.7 ± 2.7	14.3 ± 1.3	15.2 ± 0.6	14.8 ± 2.6	14.4 ± 0.7	12.5 ± 1.5	**0.021**	0.792	**0.012**	0.318	0.116	0.376	0.819
**Thr**	28.1 ± 3.4	19.1 ± 2.4	31.4 ± 4.4	21.8 ± 3	24.9 ± 1.6	18.6 ± 2.8	27 ± 1.5	18 ± 2.2	0.120	0.337	**<0.0001**	0.561	0.663	0.667	0.783
**Leu**	22.9 ± 3.8	17.8 ± 4.7	33.1 ± 8.2	16.5 ± 3.2	15.6 ± 0.6	11.9 ± 0.7	14.1 ± 0.8	11 ± 1.8	**0.0003**	0.545	**0.006**	0.275	0.148	0.289	0.240
**Ile**	10.7 ± 0.6	8.5 ± 1.1	11.2 ± 1.1	8.2 ± 0.8	10.4 ± 0.4	7.4 ± 0.5	9.9 ± 0.6	6.1 ± 0.5	**0.027**	0.336	**<0.0001**	0.363	0.369	0.493	0.920
**Asp**	6.5 ± 1.7	4.3 ± 1.3	13.6 ± 4.1	3.9 ± 0.9	4 ± 0.6	3.7 ± 0.8	3.5 ± 0.7	3.5 ± 0.3	**0.002**	0.157	**0.004**	0.081	0.007	0.100	0.068
**Lys**	46.7 ± 11.6	41.7 ± 10.8	69.7 ± 16	35.5 ± 5.9	38.2 ± 2.4	26.6 ± 2.7	36.4 ± 2	25.1 ± 1.8	**0.003**	0.523	**0.005**	0.349	0.443	0.182	0.172
**Met**	5.5 ± 0.9	3.5 ± 0.9	7.9 ± 1.7	3.3 ± 0.7	3.7 ± 0.2	2.7 ± 0.5	3.9 ± 0.2	2.5 ± 0.2	**0.001**	0.297	**<0.0001**	0.295	0.054	0.161	0.279
**His**	10.9 ± 1.1	8.9 ± 1.1	12.8 ± 1.8	9.2 ± 0.9	10.1 ± 0.8	8.3 ± 0.5	8.8 ± 0.3	8.9 ± 1.6	0.062	0.614	**0.017**	0.354	0.202	0.929	0.260
**Phe**	14.9 ± 1.3	11.5 ± 1.7	20 ± 4	11.2 ± 1.4	12.4 ± 0.3	9.6 ± 0.5	12.3 ± 0.5	10.6 ± 1.7	**0.007**	0.225	**0.001**	0.388	0.090	0.346	0.161
**Arg**	113.4 ± 11.1	116.3 ± 10.6	158.6 ± 30.9	109 ± 9.9	110 ± 11.1	115.8 ± 6.6	103.2 ± 4.8	121.1 ± 13.9	0.210	0.334	0.545	0.297	0.062	0.283	0.086
**Trp**	7.7 ± 1.3	4.5 ± 0.7	9.9 ± 1.9	5.4 ± 0.9	5 ± 0.4	5.2 ± 1.5	5.3 ± 0.5	4.9 ± 1.3	**0.034**	0.355	**0.018**	0.356	0.024	0.554	0.904
**Cys**	0.8 ± 0.2	0.7 ± 0.2	0.7 ± 0.3	0.4 ± 0.2	1 ± 0.3	0.7 ± 0.2	0.4 ± 0.1	0.8 ± 0.2	0.596	0.165	0.576	0.672	0.563	0.361	0.169
**BCAA**	52 ± 5.7	41.1 ± 7.6	65 ± 11.9	39 ± 5.2	41.2 ± 1.6	34.1 ± 2.8	38.4 ± 1.8	29.5 ± 3.3	**0.001**	0.860	**0.001**	0.263	0.199	0.301	0.421
**BCAA** (% total)	11.9 ± 0.4	10.7 ± 0.6	11.2 ± 0.4	10.4 ± 0.5	11.3 ± 0.5	10 ± 0.6	10.8 ± 0.5	8.8 ± 0.7	0.047	0.078	0.001	0.680	0.327	0.990	0.433
**Number of mice**	11	12	9	12	12	12	11	12							

S6 Table. Amino acid concentrations in plasma

Plams amino acid concentrations (ng/g) were measured by LC-MS/MS. Data for each group (sex/diet/genotype) are shown as average ± SEM. P values from 3-way ANOVA are shown for effects of each independent variable and their interactions. Asp, Gln, and Tyr were not detectable.
