# Supplementary material for: Adiponectin exerts sex-dependent effects on lipid, amino acid, and glucose metabolism during caloric restriction
Source: PLoS Biol. 2026 Jun 18;24(6):e3003821. doi: 10.1371/journal.pbio.3003821 (PMC13278438; doi:10.1371/journal.pbio.3003821)
Supplement: S7 Table — (PDF) [file pbio.3003821.s018.pdf]

REAGENT or RESOURCE	SOURCE	IDENTIFIER
**CHEMICALS, KITS and ANTIBODIES**		
D-Glucose	Sigma-Aldrich, Poole, UK	G8270
Humulin	Eli Lilly and Company, Indiana, USA	HI0210
IntraLipid	Sigma-Aldrich, Poole, UK	L141-100ML
Glycerol	Fisher Scientific, Loughborough, UK	G/P450/05
Chloroform	Fisher Scientific, Loughborough, UK	C/4960/17
TRIzol reagent	Life Technologies, Paisley, UK	15596018
Serum Triglyceride Determination Kit	Sigma-Aldrich, Poole, UK	TR0100
Ultra-Sensitive Mouse Insulin ELISA Kit	ChrystalChem, Chicago, IL, USA	90080
NEFA assay reagents	FUJIFILM Wako Chemicals Europe GmbH (Neuss, Germany)	434-91795<br>436-91995<br>270-77000
Qiagen RNeasy kit	Qiagen Ltd, Manchester, UK	74106
Microvette EDTA capillary tubes	Sarstedt, Leicester, UK	16.444
Antibody: anti-adiponectin (rabbit polyclonal)	Sigma-Aldrich, Glasgow, UK	A6354 (*RRID: AB_1078104*)
Antibody: anti-CD36 (rabbit polyclonal)	Thermo Fisher Scientific, Altrincham, UK	PA1-16813 (*RRID: AB_568487*)
Antibody: anti-ERK1/2 (rabbit monoclonal)	Cell Signaling Technology (Danvers, MA, USA)	4695 (*RRID: AB_390779*)
Antibody: anti-HSL (rabbit polyclonal)	Cell Signaling Technology (Danvers, MA, USA)	4107 (*RRID: AB_2296900*)
Antibody: anti-Phospho-HSL (Ser563) (rabbit polyclonal)	Cell Signaling Technology (Danvers, MA, USA)	4139. (*RRID: AB_2135495*)
Antibody: anti-Perilipin A (goat polyclonal)	Abcam, Cambridge, UK	ab61682 (*RRID: AB_944751*)
Total protein stain	LI-COR (Lincoln, NE, USA)	926-11010
Antibody: IRDye 800CW Donkey anti-Goat IgG Secondary Antibody	LI-COR (Lincoln, NE, USA)	925-32214 (*RRID: AB_2687553*)
Antibody: IRDye 800CW Goat anti-Rabbit IgG Secondary Antibody	LI-COR (Lincoln, NE, USA)	926-32211 (*RRID: AB_621843*)
Antibody: IRDye 680RD Donkey anti-Rabbit IgG Secondary Antibody	LI-COR (Lincoln, NE, USA)	926-68073 (*RRID: AB_10954442*)
**HISTOLOGICAL REAGENTS**		
WGA-Alexa Fluor 488	Thermo Fisher Scientific, Altrincham, UK	W11261
DAPI	Thermo Fisher Scientific, Altrincham, UK	62248
PermaFlour mounting medium	Fisher Scientific, Loughborough, UK	12695925

**MOUSE MODELS**		
Mouse: C57BL/6NCrl	Charles River, Edinburgh, UK	027
Adipoq KO	Described in “Animals”	
**SOFTWARE & PROGRAMS**		
Prism	GraphPad Software, LLC	V10.2.2
Macro Interpreter	For analysis of Promethion data	V2.46
Fiji/Image J	http://imagej.net	V2.14.0/1.54f
Adiposoft	(1).	v1.16
StarDist	https://imagej.net/plugins/stardist	
MorphoJ	https://morphometrics.uk/MorphoJ_page.html	
TrimGalore	https://www.bioinformatics.babraham.ac.uk/projects/trim_galore/	V0.6.6
FastQC	https://www.bioinformatics.babraham.ac.uk/projects/fastqc/	v0.11.7
STAR	Supplementary reference (2)	v2.7.10a
Subread	Supplementary reference (3)	v1.5.2
R	https://www.r-project.org/	v4.4.0
RStudio	https://posit.co/download/rstudio-desktop/	v2022.12.0+353
DESeq2	Supplementary reference (4)	v1.44.0
ggplot2		V3.5.1
Image Studio	Li-COR, Lincoln, NE, USA	v5.2
Metascape	Supplementary reference (5)	V3.5.20240101
Cytoscape	Supplementary reference (6)	V3.10.2
**OTHER**		
CellCrusher	CellCrusher Limited, Schull, Ireland	WC Kit
Li-Cor fluorescent blot imager	LI-COR, Lincoln, NE, USA	Odyssey CLx or Odyssey M
Nikon microscope	Nikon, Tokyo, Japan	Nikon Eclipse Ti
OneTouch Verio Glucometer	LifeScan Inc., Zug, Switzerland	User’s manual here
Promethion CORE System	Sable Systems International (Las Vegas, USA)	ExpeData software v1.9.27
TD-NMR	Bruker Optics, Billerica, MA, USA	Minispec LF90II

S7 Table. Reagents and resources used in this study.

Supplementary References cited in S7_Table

1. Galarraga M, Campión J, Muñoz-Barrutia A, et al. Adiposoft: automated software for the analysis of white adipose tissue cellularity in histological sections. J Lipid Res. 2012;53(12):2791-6.
2. Dobin A, Davis CA, Schlesinger F, et al. STAR: ultrafast universal RNA-seq aligner. Bioinformatics. 2013;29(1):15-21.
3. Liao Y, Smyth GK, Shi W. featureCounts: an efficient general purpose program for assigning sequence reads to genomic features. Bioinformatics. 2013;30(7):923-30.
4. Love MI, Huber W, Anders S. Moderated estimation of fold change and dispersion for RNA-seq data with DESeq2. Genome Biol. 2014;15(12):550.
5. Zhou Y, Zhou B, Pache L, et al. Metascape provides a biologist-oriented resource for the analysis of systems-level datasets. Nat Commun. 2019;10(1):1523.
6. Shannon P, Markiel A, Ozier O, et al. Cytoscape: a software environment for integrated models of biomolecular interaction networks. Genome Res. 2003;13(11):2498-504.
