# Supplementary material for: Adiponectin exerts sex-dependent effects on lipid, amino acid, and glucose metabolism during caloric restriction
Source: PLoS Biol. 2026 Jun 18;24(6):e3003821. doi: 10.1371/journal.pbio.3003821 (PMC13278438; doi:10.1371/journal.pbio.3003821)
Supplement: S1 Raw Images — The blots for Fig 1B (plasma adiponectin and total protein) were captured using a LiCor Odyssey CLx scanner, whereas those for S1B and S4A Figs were captured using a LiCor Odyssey M scanner. For S1B and S4A Figs, most membranes were cut before incubating in primary antibodies, allowing multiple separate incubations in different antibodies at the same time; the location of the cuts, where visible, are indicated by * in the images and, for S4B, are clear in the Coomassie scans. Because of this, the original, uncropped scans for each antibody cover only a portion of the full membrane. Note that for S4A Fig, membrane 1 was cut before incubating in primary antibodies whereas membrane 2 was first imaged for Perilipin A and was then cut between 50 and 75 kDa, with the upper portion then incubated in anti-CD36 Ab. Perilipin A used a goat secondary antibody, which visualized the 75 kDa marker but not the 50 kDa marker. (PDF) [file pbio.3003821.s019.pdf]

S1_Raw images. Unedited and uncropped full images of Western Blots used in Figure 1B

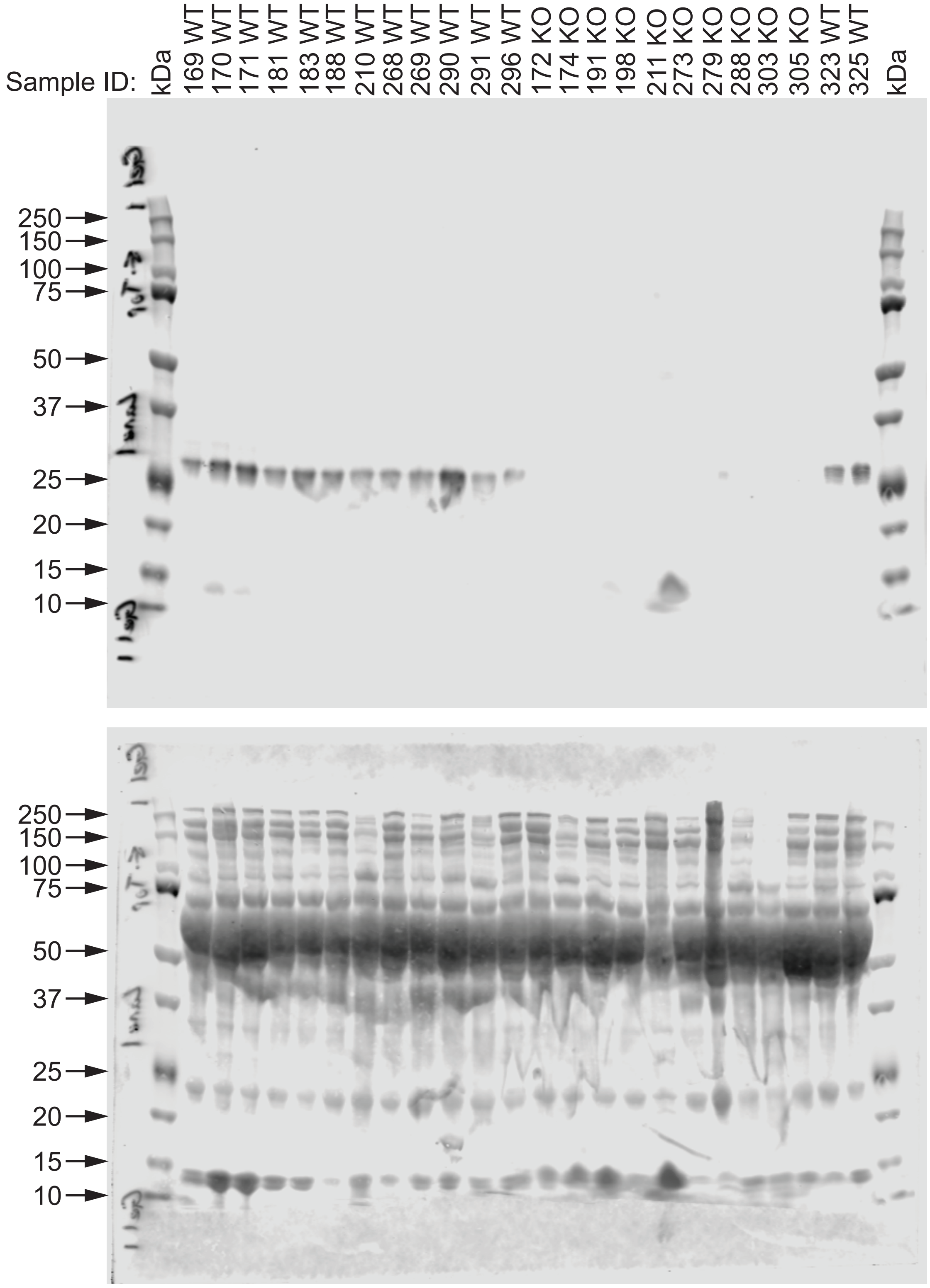

This figure shows the western blots used in Figure 1B. Images were captured using a LiCor Odyssey CLx scanner

S1_Raw images. Unedited and uncropped full image Western Blots used in Figure S1D

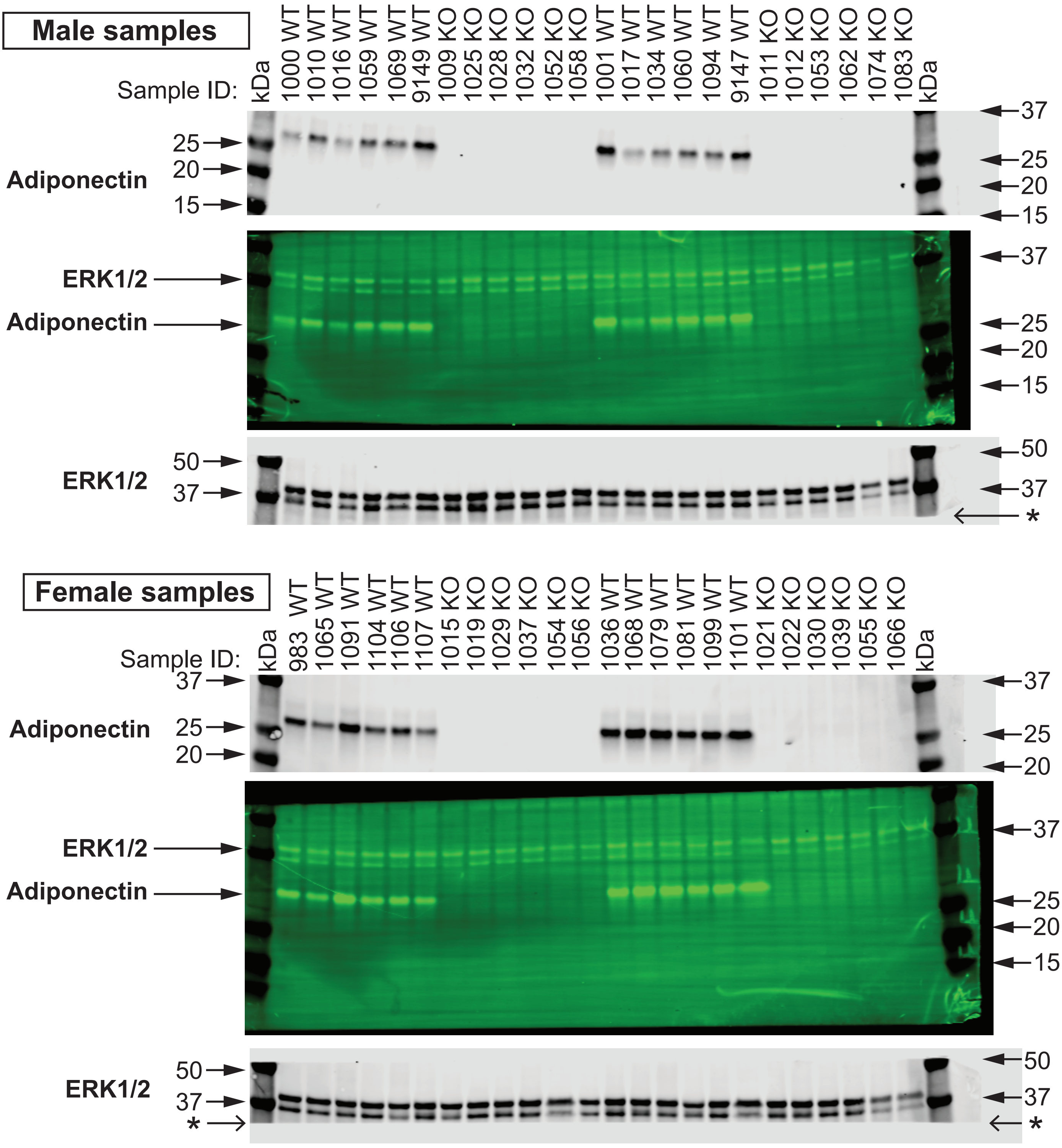

Membranes were cut at the 50 kDa marker before first incubating in anti-adiponectin Ab (top image for males and females). This portion of the membrane was then incubated in anti-ERK1/2 Ab, but when this was imaged (green fluorescent images) the adiponectin signal was still strongly visible, causing weaker ERK/12 signal (because both antibodies use a rabbit secondary). Therefore, the membrane was then cut between the 37 kDa and 25 kDa markers and reincubated and exposed for ERK1/2 (bottom image for males and females). The location of the cut, where visible, is indicated by * in the images above. Because of these cuts, the original, uncropped scans for each antibody cover only a portion of the full membrane (visible in the green fluorescent images).

S1_Raw images. Unedited and uncropped full image Western Blots used in Figure S4A membrane 1

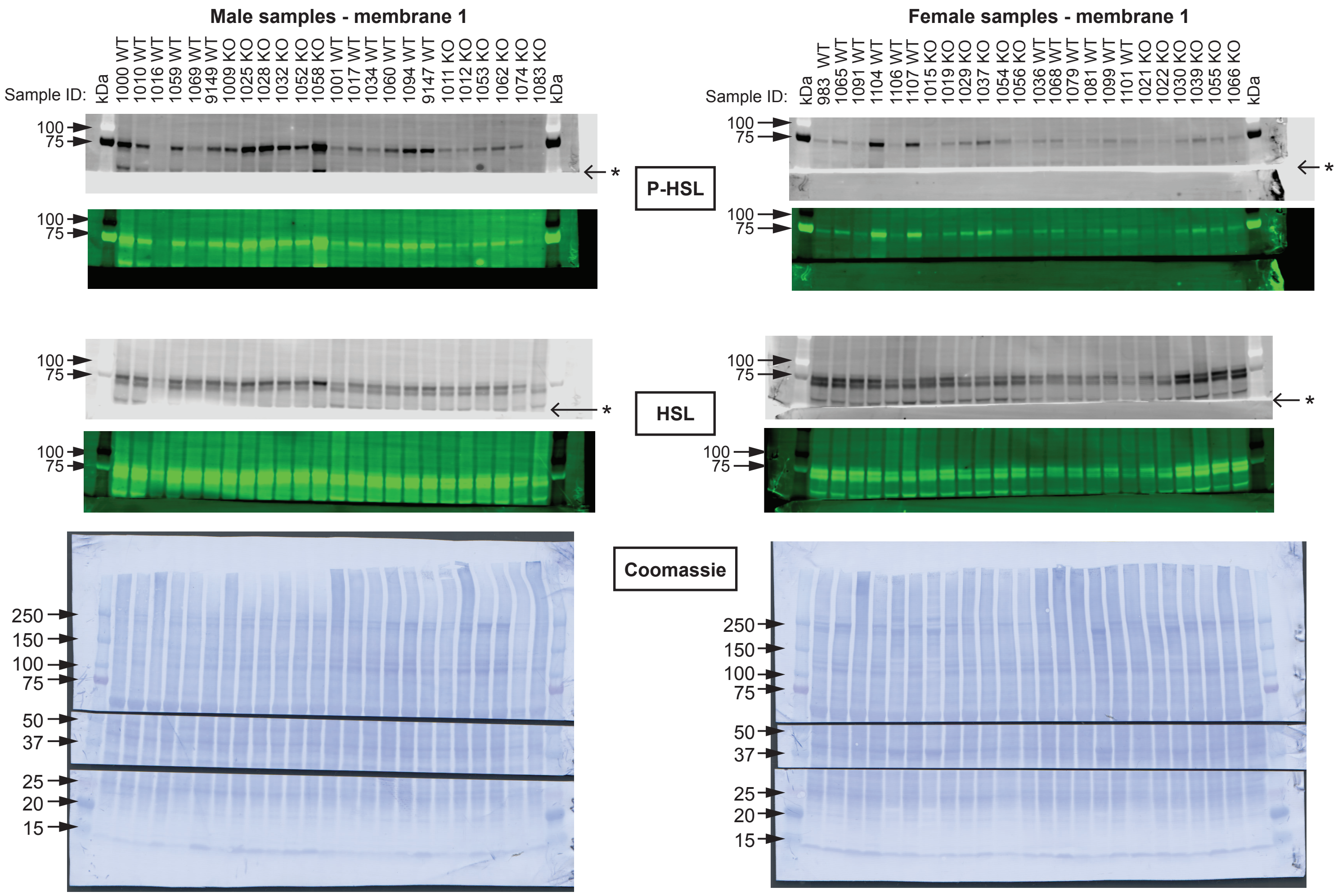

Membrane 1 was cut before incubating in primary antibodies, allowing multiple separate incubations in different antibodies at the same time. Locations of the cuts are clear in the Coomassie images and, where visible, are indicated by * in blot images. HSL and P-HSL were located in the upper portion of the membrane (visible fully in the Coomassie images). The portions visible in the HSL and P-HSL blots are smaller because, when scanned using a LiCor Odyssey M scanner, only the portion containing the visible signal (for HSL or P-HSL) was included in the scanning region. Therefore, original, uncropped scans for these antibodies cover only a portion of the full membrane. Both greyscale and original fluorescent images are shown.

S1_Raw images. Unedited and uncropped full image Western Blots used in Figure S4A membrane 2

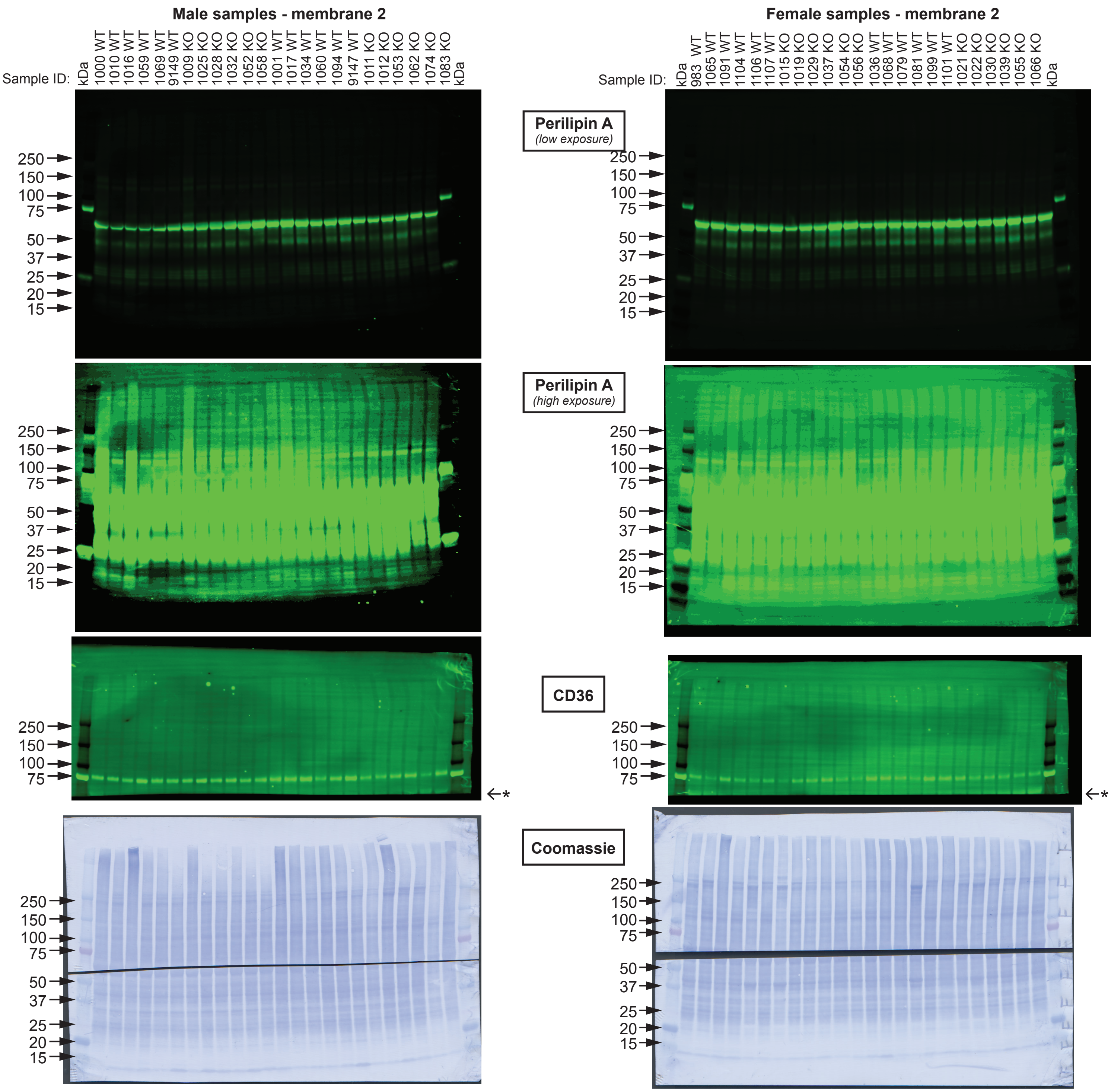

Membrane 2 was first imaged for Perilipin A and was then cut between 50-75 kDa, with the upper portion then incubated in anti-CD36 Ab. Locations of the cuts are clear in the Coomassie images and, where visible, are indicated by * in blot images. Cut membranes were scanned using a LiCor Odyssey M scanner; therefore, original, uncropped scans for CD36 cover only a portion of the full membrane.
