# Supplementary material for: Adiponectin exerts sex-dependent effects on lipid, amino acid, and glucose metabolism during caloric restriction
Source: PLoS Biol. 2026 Jun 18;24(6):e3003821. doi: 10.1371/journal.pbio.3003821 (PMC13278438; doi:10.1371/journal.pbio.3003821)
Supplement: Attachment — Submitted filename: Reviewer response_PBIOLOGY-D-25-02593R1_v20260306.docx [file pbio.3003821.s021.docx]

**Reviewer comments and responses for PLoS Biology submission PBIOLOGY-D-25-02593R1**

We thank the reviewers for their constructive feedback, which we have used to update and improve our manuscript. A summary of the major changes is as follows:

- **Definitive validation of our adiponectin KO model** beyond plasma measurements: we now include **exon-specific qPCR** showing retained low expression of Adipoq exons 1–2 but **complete absence of exon 3**, alongside **absence of adiponectin protein in WAT** (addressing recent concerns that some KO lines retain functional exon 3 expression).
- **Deepened adipose tissue phenotyping**, including **histomorphometry of iWAT and gWAT adipocytes** and expanded molecular analyses of lipid handling and adipocyte state (including markers of lipolysis and fatty-acid transport/handling). These data clarify that the KO’s effects on systemic lipids (e.g., fasting NEFA) during CR are **not explained by major CR-state increases in adipose lipolysis**, while also revealing **sex- and diet-dependent adipose phenotypes** under ad libitum feeding.
- **Added liver histology** to complement transcriptomics and biochemistry, including **quantification of lipid droplet area**. We also corrected and clarified hepatic TG analyses and updated the manuscript accordingly.
- **Improved clarity and accessibility of transcriptomic results**, including reorganising the main RNA-seq figure to present **both sexes together**, streamlining presentation, and clarifying the directionality of enrichment across conditions.
- **Strengthened reporting and interpretation of feeding/fasting context**, explicitly describing the timing of CR feeding and steps taken to ensure comparable fasting duration at cull, and adding discussion of circadian/feeding-schedule considerations and generalisability.

A full description of these updates is in the below point-by-point response to reviewers. Please refer to the ‘clean’ version of the revised manuscript for references to specific line numbers.

**Reviewer #1:** Ikushima et al. investigated the effects of calorie restriction in adiponectin knockout (KO) mice under both ad libitum and calorie-restricted conditions, in both sexes. They compared plasma adiponectin levels, body weight, and tissue weights, and found that calorie restriction significantly increased plasma adiponectin levels. However, body mass and fat mass were not different between ad libitum and calorie-restricted groups in either male or female mice.
In glucose tolerance tests (GTT), male KO mice displayed higher glucose levels under ad libitum feeding, but lower glucose levels under calorie restriction compared with wild-type controls. Interestingly, insulin levels during the oral GTT were not altered in any group. Triglyceride (TG) clearance tests showed elevated serum TG levels in male KO mice under ad libitum conditions, accompanied by increased NEFA levels, whereas females did not show these NEFA changes.
Indirect calorimetry revealed that RER was lowered under ad libitum and increased under calorie restricted condtions. Gene expression analyses revealed that male KO mice under ad libitum feeding showed upregulation of lipogenesis-related genes, while amino acid metabolic processes were enriched in calorie-restricted KO mice.
Overall, the study demonstrated that calorie restriction lowered blood glucose levels in adiponectin KO mice probably due to higher utility of carbohydrate, enhanced fasting NEFA levels, and increased postprandial RER — particularly in males — though the magnitude of change between ad libitum and calorie restriction was smaller in females than in males. To strengthen the story in this paper, the authors should address the points below.

1. The authors should present the data of a high-fat diet feeding instead of a normal chow diet and evaluate both body-weight gain and fat mass.

Response: Thank you for this suggestion, however we consider this out of scope for our CR-focussed question. The impact of adiponectin KO on diet-induced obesity has been studied extensively, whereas the goal of our study was to determine how adiponectin KO influences the metabolic effects of CR – a context in which adiponectin has received far less attention.

The appropriate control for mice undergoing CR is mice fed a control, chow diet, rather than a high-fat diet. However, we show that our adiponectin KO males on the control diet (ad libitum fed) exhibit glucose and lipid intolerance, as found for KO males on HFD or with ageing. Many of the transcriptional effects of adiponectin KO that we detect in AL mice are also consistent with those previously reported for HFD-fed males (Liu et al. 2012), something that we also now highlight in the Discussion (lines 460-473). For these reasons, it is unnecessary to include a high-fat-diet fed group in our study.

2. Since pyruvate is a major substrate for gluconeogenesis in the liver and kidney, the authors should perform a pyruvate tolerance test (PTT) in both male and female KO and wild-type mice to directly evaluate gluconeogenic capacity.

Response: Thank you for this suggestion. We planned our gluconeogenesis analyses in consultation with several other experts in metabolic tolerance tests, including Dr Samuel Virtue (Virtue et al. 2021) and Dr Adam Rose ((Okun et al. 2021)). They advised to not do pyruvate tolerance tests because the acidic nature of pyruvate can compromise the welfare of the mice, causing metabolic acidosis. We discussed this with our vets who, for welfare reasons, asked us not to proceed with pyruvate tolerance tests.

Therefore, we focussed on glycerol tolerance tests because glycerol is the major gluconeogenic substrate during CR and prolonged fasting.

3. The representation of Figure 5 and Fig. S4 should be improved for clarity. For example, for panel E, the legend and figure should clearly state which pathways are enriched in which genotype (not describe "wild-type vs KO", but clearly state "enriched in wild-type" or "enriched in KO"), rather than using the term "between WT and KO", "WT vs KO". This will make the interpretation more straightforward for readers.

Response: Thank you for suggesting ways to improve our presentation of the liver RNA-seq results; we apologise for any confusion. In Fig.5E, 5F, S4D, and S4E (from our original manuscript), the enriched pathways are based on the transcripts differentially expressed between WT vs KO: some transcripts are enriched in WT and some are enriched in KO. Therefore, these figures don’t indicate whether individual transcripts are elevated or decreased by adiponectin KO, but rather what biological process are regulated (up or down) with adiponectin KO, based on the transcriptional changes. Effects on specific transcripts relevant to these pathways are shown in the volcano plots (Figure 5B). To clarify the direction of the KO effects, we also show GSEA results in Fig. 5H (Fig. 5G of original submission) and Figures S7A-B and S8-A-B (Figure S5 of original submission). These results clearly indicate the enrichment of genesets in WT or KO under each sex/feeding condition. We have updated the figures and legends so that this point is clearer.

Please note that reviewer 4 (major point 7) asked us to show both male and female data in main figure 5. Therefore, we have updated figure 5 so that it shows both male and female data. We have deleted the heatmaps (original figure 5D and previous Supplementary Figure S4) as these are not essential for interpreting our key findings and we feel that streamlining the results improves the readability of the paper.

4. The authors showed that male adiponectin knockout (KO) mice have elevated serum triglyceride (TG) and NEFA levels, as well as upregulated amino acid catabolic pathways in the liver and higher RER. This suggests that, despite the presence of abundant circulating lipids, the KO mice preferentially utilize carbohydrate-derived energy — likely by catabolizing amino acids to support gluconeogenesis. The authors should further explain or discuss this apparent metabolic preference in adiponectin KO mice, particularly under calorie-restricted conditions, where the mice seem to favor carbohydrate production and utilization despite having elevated circulating lipids.

Response: Thank you for highlighting this point, which reflects the complexity of our findings. You state that the KO mice have elevated RER, but this is not the case: in CR, females maintain a higher RER during the fasted (night) phase but have a lower post-prandial RER. In contrast, the males have a higher RER immediately after refeeding, but not at any other time point. We highlight this subtle point in our summary figure (Figure 6). In addition, during CR, adiponectin KO mice tend to have lower fasting glucose than WT mice, which does not suggest increased carbohydrate production in the CR KO mice. Our interpretation is that, in CR males, KO mice utilise carbohydrates more efficiently after feeding, which may be why they have a lower glucose spike in the OGTT. As you note, the KO mice may also favour amino acids as a gluconeogenic substrate during CR. This could contribute to lower fasting glucose because gluconeogenesis from amino acids is less efficient than from glycerol.

We agree that these points are complex, particularly as many of the KO effects differ strongly depending on sex and/or diet. We attempted to summarise these points in lines 344-356 and 373-385 of our original submission, as well as Figure 6. We have updated Figure 6 to further clarify that amino acid-driven gluconeogenesis may be enhanced in KO CR mice. We have also addressed these points in lines 380-393 and 426-438 of our revised paper (moving some interpretation from the Results to the Discussion, as requested by Reviewer 4).

5. Regarding Fig. 6, bone marrow and adipose tissue illustration is not well supported by the original data. The authors should measure adiponectin expression in bone marrow and adipose tissue in each condition.

Response: Thank you for this suggestion. Previous studies from us and others showed that, during CR, bone marrow adipose tissue (BMAT) is the primary source of circulating adiponectin; this includes data showing that CR increases adiponectin expression in bone marrow but not in white adipose depots (Cawthorn et al. 2014, Scheller et al. 2016) – a finding that we repeat in our revised manuscript, at least for male mice (new Supplementary Figures S1D-E). Therefore, we indicated this in our original summary figure (Figure 6). However, because we did not assess BMAT-derived adiponectin production in our present study, we have updated Figure 6 to remove BMAT.

Reviewer #2: This manuscript focused on the role of adiponectin during caloric restriction. The authors found that adiponectin KO mice do not have altered body weight and body composition in both sexes. Interestingly, in male mice, the improvement in glucose tolerance due to caloric restriction is more pronounced in adiponectin KO mice. These male adiponectin KO mice had higher fatty acids in their fasting blood, as well as impaired lipid clearance after oral lipid administration. Female adiponectin KO mice showed relatively milder alterations among all the assays. RER showed that both male and female adiponectin KO mice had a lower RER, indicating more fatty acid usage compared to carbohydrate usage.
Overall the manuscript presented interesting observations suggesting there is sexual dimorphism in the role of adiponectin during caloric restriction. However, it would be nice if the authors could provide more mechanistic insights into the sexual dimorphism in adiponectin function. I also have a few suggestions that could potentially improve the manuscript.

Response: Thank you for highlighting the interesting observations in our manuscript, including the strong sexual dimorphism. We agree that this would be interesting to further explore mechanistically, and a major interest in our lab is to understand why the effects of CR itself (even in WT mice) differ so strikingly between males and females (Suchacki et al. 2023). Mechanistic exploration of this typically relies first on gonadectomy studies to determine the contributions of sex hormones, followed by genetic manipulation of sex steroid receptors to further test downstream and tissue-specific mechanisms (Mauvais-Jarvis et al. 2017). Before understanding sexual dimorphism in adiponectin function, we think it is necessary to understand the basis for CR’s sexually dimorphic effects in WT mice.

These mechanistic analyses are extensive and therefore beyond the scope of the present paper. However, a firm conclusion, stated at the end of the Discussion, is that biological sex strongly influences the impact of adiponectin deficiency and that future studies must further establish the extent of, and basis for, adiponectin’s sexually dimorphic effects

1.	Please add validation of adiponectin knockout.

Response: In our original submission we showed that plasma adiponectin is undetectable in the KO mice (Figure 1B). To further validate (including addressing concerns raised by Reviewer 3), we have now analysed adiponectin transcript and protein expression in white adipose tissue. This includes separate analysis of the mRNA sequences for exons 1-2 (encoding the collagenous domain) and exon 3 (encoding the functional globular domain, which comprises >70% of the protein-coding sequence and which is deleted in our KO model). These data, shown in new Supplementary Figures S1B-E, show that KO mice continue to express low levels of exons 1-2 in WAT but have no expression of exon 3 and a complete absence of adiponectin protein. These data conclusively validate our KO model.

2.	Other than reduced fatty acid oxidation, increased fasting free fatty acids in the circulation could also be due to altered lipolysis from the adipose tissue. Actually, RER shows the KO mice prefer fatty acids as a fuel. An in vivo lipolysis test is suggested to be done in parallel with the OLTT.

Response: We agree that increased NEFA in the KO mice during CR could be a result of increased lipolysis. In our original submission, we did not see any effect of the KO on WAT mass or total fat mass during CR (Figure 1), suggesting that the NEFA differences are unlikely to result from increased lipolysis in the KO mice – if so, we might expect lower fat mass in the KOs. The RER data do show lower RER in the fasting (daytime) period in the KO males and females on AL diet, but do not support increased fatty acid use in the KO mice during CR. Instead, on CR diet, female KOs have higher fasting (nighttime) RER, suggesting impaired lipid oxidation.

To further explore if KO alters lipolysis in adipose tissue, we used immunoblotting to measure HSL phosphorylation and perilipin A expression in gWAT, and histomorphometry to measure adipocyte size distributions in gWAT and iWAT. We acknowledge that these are not direct tests of lipolysis in vivo; such experiments could not be done for logistical reasons (i.e. time and costs required to breed sufficient numbers of mice to achieve adequate statistical power for these studies). However, HSL phosphorylation and adipocyte size are robust proxies for lipolytic activity in WAT. These new data, shown in new Figures 3E-F and S3-S5, show that, on AL diet, KO decreases adipocyte size in males and increases this in females, especially in iWAT. KO AL males also have greater HSL phosphorylation and perilipin A expression than WT AL males (Supplementary Figures S4A-B); hence, increased lipolysis may explain the adipocyte hypotrophy in KO AL males. This finding is consistent with data showing that adiponectin suppresses lipolysis in gWAT adipocytes from control-fed male mice (Qiao et al. 2011).

We find subtle effects on CD36 expression: KO significantly decreases this in females and tends to do so in males during CR (Figure S4). This might contribute to the increased fasting NEFA in these mice (Daquinag et al. 2021). However, we show that on CR diet KO does not affect adipocyte size or HSL activation in either sex (Figures 3E-F, Supplementary Figures S3 and S4). These data indicate that adiponectin KO does not alter lipolysis during CR.

We have updated the Results and Discussion of the manuscript with these important new findings, which help to understand the KO effects on systemic lipid metabolism.

3.	It would be nice to validate the genes related to fatty acid transport, oxidation, and synthesis in the liver and adipose tissue in both sexes.

Response: Thank you for this suggestion. For the liver, our GO and GSEA data show how transcriptional effects relate to genes involved in FA synthesis and catabolism; these are shown in Figure 5 and in Supplementary Figure S7C (Figure S5C of the original manuscript). There are 182 genes for the MM4791_GOBP_FATTY_ACID_BIOSYNTHETIC_PROCESS gene set and 122 for the MM5199_GOBP_FATTY_ACID_CATABOLIC_PROCESS gene set (Figure S7C). Therefore, in the manuscript it is unfeasible to clearly present these data on the level of individual genes. Instead, our preference is to focus on the summary data provided by the GO and GSEA analyses. KO does not alter FA catabolism in any sex-diet subgroup (Figure S7C); however, FA biosynthesis is enriched by KO in AL males and females, identifying this as a more-robust KO effect. Therefore, we have updated Figure S7 to show the KO effect on key genes in the ‘GOBP_FATTY_ACID_BIOSYNTHETIC_PROCESS’ pathway (*Fasn, Acaca,* and *Scd1*) in AL mice of each sex. To accommodate this, we have moved the GO analyses of CR mice (previous figures S5D-E) to Figure S8 of the revised manuscript.

Although we have space to show only these key genes, readers will be able to directly assess expression of any other gene of interest using the raw RNAseq data that we will deposit through NCBI GEO (our WT liver data is already available with accession number GSE230402).

For adipose tissue, we have used qPCR and western blotting to assess expression of key genes and proteins in these pathways. These new analyses are shown in Figures 3G and S3-S5 of the revised manuscript. The results are interesting and complex, with KO effects often being diet- and/or sex-dependent.

In addition to the effects on adipocyte size and lipolytic markers (described above in response to your second point), we show that KO influences expression of several genes relating to adipose lipid metabolism. Across all sex-diet subgroups, fatty acid-binding protein 4 (*Fabp4*) and peroxisome-proliferator activated receptor-a (*Ppara*) are increased by adiponectin KO, particularly in CR males (Figure 3G). KO also moderately increases expression of carnitine palmitoyltransferase 1A (*Cpt1a*), the rate-limiting enzyme for FA oxidation. In contrast, CR increases expression of lipoprotein lipase (*Lpl*), fatty acid synthase (*Fasn*) and diacylglycerol O-acyltransferase 2 (*Dgat2*), three critical regulators of lipid uptake and storage, but their expression is unaffected by adiponectin KO (Supplementary Figure S5B).

Reviewer 3 asked us to also analyse regulators of adipogenesis, so we also analysed expression of *Pparg2, Cebpa* and *Dlk1* (Pref1; Supplementary Figure S5). Adiponectin KO decreases *Dlk1* expression and tends to increase *Pparg2.*

These new results show that adiponectin KO has diverse effects on WAT morphology and molecular characteristics. However, while the small changes in CD36 may contribute, the overall KO effects on WAT suggest that altered WAT function is not a primary mediator of increased plasma NEFA or impaired lipid clearance in KO mice. For example, increased *Fabp4, Ppara* and *Cpt1a* expression in KO WAT might be expected to decrease plasma NEFA, because *Fabp4* deficiency increases plasma NEFA during fasting (Syamsunarno et al. 2013) and PPARα and CPT1a promote adipocyte FA oxidation (Goto et al. 2011). The KO-associated increases in *Pparg2* and decreases in *Dlk1* – an inhibitor of adipogenesis – suggest enhanced adipocyte lipid storage capacity (Hudak et al. 2013), yet KO does not influence expression of lipoprotein lipase, *Fasn* or *Dgat2.* This suggests that altered WAT lipid uptake does not mediate the impaired lipid tolerance of KO males, and may explain why overall fat mass and adipocyte sizes are similar in KO vs WT mice during CR.

Together, it is clear that adiponectin KO exerts diet- and sex-dependent effects on WAT, but the systemic relevance of these remains to be determined.

We report these new analyses and their interpretation in the Results and Discussion of the revised manuscript (lines 178-207 and 395-414).

4.	Please also validate some of the glucose-related pathways discussed in the liver and confirm sexual dimorphism at the molecular level.

Response: In our original submission we showed that KO alters the expression of gluconeogenesis-related genes in CR males only (Figure 5F, GO analysis). We also showed counts for two genes in this pathway, *Gpd1* and *Gpd2*, to demonstrate the diet- and sex-specific effects of the KO, and because these two genes are relevant to gluconeogenesis from glycerol; these data were in Supplementary Figures S9A-B of our original submission. We agree it is helpful to further present key molecular changes that contribute to this KO effect. Therefore, in our revised manuscript we show data for three other key genes, *Got1, Sds* and *Fbp2*, that differ between WT vs KO males during CR but are less influenced by KO in the other diet-sex groups. Both *Got1* and *Gpd1* show clear sex-specific effects of adiponectin KO. These new data are shown in Supplementary Figures S11C-E (along with *Gpd1* and *Gpd2*).

It is challenging to show all the genes included in the gluconeogenesis pathway because this GO term (GO:0006094: gluconeogenesis) includes 106 different genes. However, the above transcripts illustrate the diet- and sex-specific effects of adiponectin KO on transcripts related to gluconeogenesis, while the GO analyses and volcano plots in Figure 5 demonstrate that this effect also applies across a wide range of gluconeogenic genes. As above (for genes relating to FA metabolism), expression of any other genes of interest can be done using the raw RNAseq data that we will deposit through NCBI GEO.

**Reviewer #3:**
This investigation examined whether adiponectin mediates the metabolic effects of caloric restriction. The authors generate a novel adiponectin KO mouse model via the excision of exon 3, which is noteworthy since two prior adiponectin KO models retain exon 3 encoding the bioactive domain. A bioRxiv preprint (<https://www.biorxiv.org/content/10.1101/2025.07.12.664095v1>) suggests that those KO mice display residual effects if adiponectin vs the model that achieves a complete knockout of all three exons. The effects of adiponectin KO shown here are mild, but this could be because the mice were chow-fed. Adiponectin deficiency did not alter body mass or fat mass under CR. However, the authors show the exciting findings for how adiponectin regulates hepatic gene expression. Under ad libitum feeding, adiponectin KO alters lipid metabolism genes, and during CR adiponectin KO increases genes related to amino acid catabolism. The inclusion of both sexes is admirable and provides useful information, since females naturally have higher adiponectin levels, but in this study have similar responses to males. The phenotyping is thorough, and the RNA-seq data from the liver are informative. However, the manuscript requires additional data and clarification to strengthen the conclusions about the systemic interactions of adiponectin during CR.

Major comments

1. Adiponectin mRNA from exon 1 should be assessed to confirm the absence of full-length transcripts and validate the KO model. This will distinguish this model from prior partial KOs. This technical advancement should be explicitly emphasized as a strength in the manuscript.

Response: Thank you for this raising this important point. To further characterise the KO, we have now analysed adiponectin transcript and protein expression in white adipose tissue. This includes separate analysis of the mRNA sequences for exons 1-2, which were not targeted by our KO strategy, and exon 3, which is fully deleted in our KO model. These data, shown in new Supplementary Figures S1B-E, show that KO mice continue to express low levels of exons 1-2 in WAT but have no expression of exon 3 and a complete absence of adiponectin protein. This conclusively validates our full KO model.

In the updated manuscript we discuss these findings in the Results (lines 119-124) and Discussion (lines 364-369), putting them in the context of the findings of the recent bioRxiv preprint (Onodera et al. 2025).

2. Adipose tissue phenotype has not been analyzed in sufficient depth. Since both adiponectin and CR have large effects on adipose biology, inclusion of adipose tissue histology staining of iWAT and gWAT and assessment of adipose tissue quality are essential. The fat mass may not change, but there can be significant changes in adipose tissue function. Moreover, expression of key adipocyte lineage and lipogenic genes (Ap2, Cebpa, Pparγ, Plin1, Pref1) should be assessed to clarify adiponectin's role in adipose adaptation during CR. For these reasons, I suggest removing this statement. "Line 358. It remains less clear how KO increases fasting NEFA in CR males. This is unlikely to reflect greater lipolysis, because adiponectin KO does not alter adiposity or CR-induced fat loss."

Response: Thank you for this suggestion. We have now analysed WAT in more detail, including histomorphometry of adipocyte sizes, qPCR for adipocyte lineage genes (*Fabp4, Cebpa, Pparg2, Dlk1 [Pref1[),* and qPCR or immunoblotting for genes and proteins relating to lipid metabolism (*Fasn, Dgat2, Lpl, Cpt1a, Ppara,* CD36, Perilipin A, and HSL phosphorylation). As you predicted, adiponectin KO has various effects on these readouts. We explain these in detail in the Results and Discussion of our manuscript (lines 178-207 and 395-404). These new analyses show that adiponectin KO exerts diet- and sex-dependent effects on WAT, something that we now highlight further in the updated summary figure (Figure 6).

3. RNA-seq findings suggest major transcriptional changes in hepatic lipid handling. Yet there is no histological assessment of the liver. Since liver size decreases under CR without concurrent reductions in triglyceride levels (Fig. S3B), liver histology is essential to corroborate the RNAseq data. This is especially important considering there were the sex differences.

Response: Thank you for this suggestion. We have now done liver histology to quantify lipid droplet area. We did not have frozen sections and so quantified lipid droplets based on the overall area of white vacuoles in the FFPE sections.

These new data (shown in Supplementary Figure S6C-D of our revised submission) show that, when assessed across both sexes and genotypes, CR decreases liver lipid droplet accumulation. This is consistent with our analysis of hepatic TG accumulation (Figure S6B); *note that we have updated these TG after identifying a small error in our calculations (our original submission presented data for total glycerol concentration, i.e. free glycerol plus esterified glycerol, rather than true TG concentrations).*

Our analyses show that adiponectin KO has no effect on lipid droplet area in AL or CR males or females, consistent with the TG concentrations. This is seemingly at odds with our RNAseq data showing that adiponectin KO enhances expression of genes relating to lipid localisation and synthesis in males and females under AL diet (e.g. Figure 5 and Supplementary Figure S7 of revised manuscript). One possibility is that analysis of gross lipid content are not sufficiently sensitive to detect KO effects on lipid metabolism, as captured by the liver RNAseq data.

Lipidomics is one more-sensitive approach to consider. Because adiponectin has been strongly implicated with hepatic sphingolipid metabolism, we previously used lipidomics to quantify sphingolipid species in KO vs WT livers. However, these analyses also revealed no effects of adiponectin KO (Supplementary Tables 1-3). Therefore, it may be that metabolic tracer studies are needed to more-comprehensively establish how adiponectin KO impacts hepatic lipid metabolism in AL and CR mice. We currently do not have funding for such analyses and are aware that our manuscript already contains an extensive amount of data, so we instead have highlighted this in the Discussion as an important future direction.

4. Did the adiponectin KO mice lose their hair? If so, a photo should be included. Alopecia can affect thermogenesis in mice housed at ~22°C and may explain the RER results.

Response: This is an interesting possibility, but we have never observed hair loss in the KO mice. KO also does not increase energy expenditure on AL or CR diet (Figure 4D-F) and brown adipose tissue (BAT) mass is unaffected by KO (now shown in updated Figure 1E). Therefore, there is no evidence that the KO mice have increased susceptibility to cold stress.

Minor comments

The 2012 Harvey Lodish paper also examines adiponectin KO effects on liver gene expression on a chow diet and should be referenced. They have similar findings that could be discussed/mentioned.

Response: Thank you for highlighting this. We are very familiar with the excellent Lodish 2012 study and had cited it in an earlier draft of our paper, but this was removed when shortening the manuscript before our first submission. We have now restored this text to the Discussion (lines 460-473). Although the Lodish study investigated HFD-fed mice, many of their reported KO effects are consistent with our findings in chow-fed (AL) mice. Comparing our studies is important because it helps to identify the common vs diet-dependent effects of adiponectin KO in the liver.

The term hyperadiponectinemia is typically associated with a disease state; The increase in adiponectin seen due to CR is modest vs levels associated with this clinical term.

Response: We used this term because it refers to increased plasma adiponectin, but we agree it can be more common to refer to pathological, supraphysiological increases in adiponectin. Therefore, we have now replaced it with “increased adiponectin” or “elevated adiponectin”, as suggested.

The text reads that the lack of effect on hepatic ceramides in the KO mice is "striking." This is misleading, as ceramide modulation by adiponectin has primarily been observed under high-fat feeding. Please rephrase. It makes sense CR lowers ceramides due to decreased dietary fat and enhanced insulin sensitivity. (It is very nice to see all the data on all the different ceramide species.)

Response: Thank you for highlighting this and the benefits of showing data for all the ceramide species. We have updated the text from “Strikingly, adiponectin KO did not alter ceramides, DHCs, or the ceramide: DHC ratio...” to “However, adiponectin KO did not alter ceramides, DHCs, or the ceramide: DHC ratio...”.

Please remove the identified outliers in Supplementary Fig. S3B and S3C, which are >3 SD from the mean, as they skew the interpretation of liver TG content.

Response: We checked the data originally shown in Supplementary Figures S3B-C. The female KO CR group does have one very high datapoint in each figure (value of 67.6 mg/g in S3B and 60 mg in S3C), but these are not >3 SD from the mean in either case (Figure S3B: max = 67.6, mean = 23.1, SD = 19.1. Therefore mean + 3 SD = 80.4) (Figure S3C: max. = 60, mean = 18.1, SD = 18.1. Therefore mean + 3 SD = 72.4).However, as noted above, in reviewing this data we identified a small error in our calculations: our original submission showed the concentrations of TG plus free glycerol, rather than TG only. We have now updated the analysis to show concentrations of triglycerides only; this is shown in Supplementary Figure S6 (an updated version of previous figure S3). We also no longer show total TG content, following your recommendation in minor point 3.

With these updated data, we tested for outliers using the Rout method (Q=1%). This identified one statistical outlier (from KO CR females), which we have excluded from the analyses. Our corrected data now show a significant diet effect across all groups, but no KO effect or KO*diet interactions.

We have updated the legend to Figure S6 (previous figure S3) to explain that one data point was identified as a statistical outlier and removed. Three outliers were also identified for our new analyses of liver lipid droplet accumulation (now shown in Figure S6C-D).

Consider discussing the potential limitations of using mice on a chow diet instead of starting out with an obese mouse or a more physiologically challenging context for liver TG accumulation, which might better reveal the role of adiponectin in the liver.

Response: We agree that beginning with obese mice could reveal additional effects of adiponectin during a dietary weight loss intervention. However, many previous studies have already investigated how adiponectin KO impacts liver function in obesity; our goal was to test if it alters liver function and systemic metabolism during CR, for which the AL chow-fed mice are considered the most appropriate controls. This rationale is similar to gold-standard human CR studies, such as the CALERIE trial, which studied non-obese participants (Kraus et al. 2019).

To better highlight how our liver data relate to previous studies of adiponectin, including in obese mice, we have updated the Discussion of our revised manuscript (lines 460-473). Our finding that adiponectin KO does not affect hepatic TG content in chow-fed (AL) mice is consistent with earlier reports showing that adiponectin KO does not alter hepatic TGs in chow- or HFD-fed males (Maeda et al. 2002) or affect liver TG secretion (Qiao et al. 2008). Many of the transcriptional effects of adiponectin KO that we detect in AL mice are also consistent with those previously reported for HFD-fed males (Liu et al. 2012), something that we also now highlight in the Discussion. Therefore, even though our control AL group is not obese, they still demonstrate many KO effects that are similar to those in obese mice.

Reviewer #4: This manuscript by Ikushima et al describes the effects of adiponectin KO on the response to CR in both males and females.
Overall comments: this is a nice paper and is easy to read and follow. It could benefit from being shortened and this could come partly from a smaller amount in interpretation in the results section. Also, I believe there is a bit of overinterpretation of small changes that occur in some measurements.

Response: Thank you for kind comments. Considering the complexity of our findings (including the diet- and sex-dependence of the KO effects), we feel that including some interpretation in the results is helpful in explaining the logical progression of our experiments. However, we agree that overly long manuscripts can be off-putting for readers, so have cut some interpretation from the Results, particularly in the indirect calorimetry section (as this interpretation was repeated in the Discussion).

Major comments
1.	Please report when you fed the CR mice, i.e morning or afternoon, as this potentially has an effect on circadian rhythms. Looking at the RER it seems like the authors have fed in the morning which is why the curves have the increase in the morning while the ad lib group increase in the evening. Previous studies have shown that feeding in the light cycle vs the dark will affect transcriptional and protein profiles. Since the authors go onto investigate the differences in transcriptional profiles, can they assure the reader that they are actual changes and not just because their circadian rhythm has been disrupted. This needs to be addressed in the discussion as a major limitation of the study that may affect interpretation of outcomes and conclusion.

Response: We agree that morning feeding will influence circadian rhythms. To clarify this, we have updated the Methods to specify that the CR mice were fed in the morning (this information was previously included indirectly, by citing one of our previous CR studies; however, we agree it is helpful to explicitly state this in the methods section). We have also highlighted the issue of day vs night feeding in a new paragraph in the Discussion (lines 494-504), including evidence that our findings are generalisable to other CR regimens.

2.	Due to the CR feeding period being in the morning, was the fasting period the same at cull between the two groups? This could have implications for the transcriptomics above, but also the NEFA concentrations in figure 3.

Response: Thank you for highlighting this important point. To mitigate any differences caused by feeding CR mice in the morning, at the time of cull we ensure similar fasting durations between CR and AL mice. To do so, all mice are provided with half of their usual daily food allotment on the evening prior to cull, resulting in fasting for ~6-12 hours before culling and sampling tissues. This ensures that any AL vs CR differences are not simply a result of a longer fasting duration in the CR mice. This approach is recommend by other groups with longstanding expertise in CR research (Lusseau et al. 2015, Mitchell et al. 2015, Mitchell et al. 2015, Mitchell et al. 2015, Derous et al. 2016, Derous et al. 2016, Mitchell et al. 2016, Derous et al. 2017, Derous et al. 2017, Green et al. 2017, Mitchell et al. 2017, Green et al. 2018). We have updated this information provided in the Methods of our revised manuscript.

3.	Measuring blood/plasma insulin levels throughout a GTT is not GSIS, this is a separate assay that is done ex vivo. What you are measuring during a GTT is both release from the pancreas as well as clearance through the liver. Thus, I suggest that you rename this to plasma levels during a GTT and change the wording around this particular result.
Response: Thank you for this suggestion, we have updated the paper accordingly.

4.	I believe that too much is being made out of the very small difference seen in the oGTT curves between the genotypes and CR (only different at 15 min in males). The AUC should be the focus of the statistics and discussion.

Response: We agree that the differences in the OGTT at individual time points are subtle; however, robust effects of adiponectin KO are revealed by 3-way ANOVA, especially in the males (i.e. *P* [KO*Diet] = 0.0015). The power of doing 3-way ANOVA is that it allows us to detect interactions between KO, diet, and temporal changes in glucose during the120-min OGTT protocol. This is demonstrated by the female analyses, where ANOVA finds a significant KO*Diet interaction, despite no differences at the individual timepoints.

AUC is helpful in providing a summary across the whole timecourse, but it does not capture these dynamic changes and how they are altered by KO and diet. Therefore, we decided to focus on the 3-way ANOVA results across the OGTT timecourses.

5.	Regarding the lipid tolerance test: "The ability to quickly normalize hyperlipidemia following the administration of lipids provides integrated information about intestinal lipid absorption, lipid transport via lipoproteins, and tissue-specific lipid metabolism." PMID: 33022003. Therefore, just focusing on the potential for FAO being the only proposed mechanism for the differences seen is too reductionist.

Response: Thank you for highlighting this point, which we have addressed through several updates. In the Results section (lines 180-182), before presenting our new data on the WAT phenotype, we now write, “The KO effects on plasma NEFA and lipid tolerance might result from KO increasing lipolysis, increasing FA synthesis, decreasing FA uptake, and/or decreasing FA oxidation in WAT.” We then present new analyses of WAT that address these possibilities (see our responses to Reviewer 2 [point 3] and Reviewer 3 [point 2]).

We have also updated the Discussion (lines 487-488) to state, “impaired lipid clearance in KO males could result from effects on other organs, such as impaired intestinal lipid absorption” and interpret the lipid clearance phenotype in light of our new data for WAT.

6.	Regarding the RER in females: Could it not be that the female KO CR mice have increased metabolic flexibility as they are better able to use the fuel source available at the time - i.e. CHO during the day when fed and fat when fasting? Saying that, they are small changes.

Response: We agree that the RER data indicate altered metabolic flexibility in the females, but think this points to impaired flexibility: their RER tends to be lower in the day (after feeding), indicating impaired CHO use, but it is higher in the night, indicating impaired fat oxidation. We have updated the discussion with this interpretation, writing (lines 386-393) “in CR females, adiponectin KO *attenuates* the postprandial rise in RER, which may explain why KO does not suppress OGTT peak glucose in CR females. Instead, KO suppresses the decline in RER as CR females progress from the fed (daytime) to fasted (nighttime) state. This suggests that, under CR, KO females resist the increase in lipid oxidation and/or sustain elevated carbohydrate oxidation during the onset of fasting, **indicating impaired metabolic** **flexibility**. These effects may underlie the lower OGTT blood glucose and increased fasting NEFA observed in KO vs WT females during CR.”

7.	Figure 5: why do you present the male data here but put the females in the supp figure? While I realise that space is at a premium in figures, it is highly irritating having to go back and forward between the two figures to compare. Also considering the title of the paper as well as the figure legend, being able to compare the males and females easily is needed.

Response: We agree that it is better to show male and female data together in the main figures. We have updated main Figure 5 by incorporating female data that was shown previously in Figure S4. We have removed the heatmaps that were previously shown in Figure 5 and Figure S4 because we felt these were not essential (we have tried to minimise inclusion of non-essential data, to improve readability of the paper). Thank you for giving us the opportunity to improve our figures.

Minor comments
1.	Why were the GTT and Intralipid doses based on BW while the insulin was based on lean mass? Lean mass would have been the better option for at least the GTT.

Response: Glucose, intralipid and glycerol were dosed against total body weight to account for differences in adiposity; this is important because adaptations in adipose tissue contribute to CR’s systemic metabolic effects (we have taken the same dosing approach in our previous studies (Cawthorn et al. 2014, Suchacki et al. 2023)). For ITTs, we decided to dose relative to lean mass because CR mice are prone to pathological hypoglycaemia following insulin administration. We found that dosing insulin relative to lean mass gives a more predictable response, with lower incidence of hypoglycaemia, and therefore used this method for our studies. This shouldn’t compromise the biological interpretation, given that skeletal muscle and the liver are the main mediators of decreasing blood glucose in response to insulin. We now explain this rationale in the Methods section.

2.	Line 134-135 "These data confirm our previous findings that CR has a weaker effect on glucose tolerance in females than in males (5)." Is weaker really the right word here - maybe smaller or lesser effect would be better? This is relevant for the rest of the manuscript as well.

Response: Thank you for this suggestion. We agree that “smaller effect” is more accurate than “weaker effect”, so we have updated this throughout the manuscript.

3.	Supp Fig 3B and C - I don't understand why the need for the two measured, content (B) is usually what is presented in publications. And considering the large amount of data presented, reducing anything that is not necessary would be beneficial. Also why were the lipidomics only done on the males?

Response: We presented both TG concentrations (Supp Fig 3B) and overall liver TG content (Supp Fig. 3C) because the livers of the CR mice are smaller; therefore, even if concentrations were similar, total content may differ. However, we acknowledge that most previous studies of adiponectin KO or CR measure hepatic TG concentrations (e.g. (Zhu et al. 2004, Liu et al. 2012)) and that the absolute TG content does not provide any insights beyond those from the TG concentrations. Therefore, we have removed the data in Supp Fig 3C, as suggested (note that this is no Figure S6 of the revised manuscript).

Note that, in reviewing this data, we identified a small error in our calculations: our original submission showed the concentrations of TG plus free glycerol, rather than TG only. We have now updated the figure to show concentrations of TG only.

Regarding lipidomic analyses of ceramide concentrations, we focussed on males for several reasons. Firstly, males show much stronger KO and KO*diet effects than females, so we reasoned that, if there are no KO effects on ceramides in males, there are unlikely to be KO effects on these in females. Secondly, we previously showed that, in wild-type mice, CR alters hepatic sphingolipids far more strongly in males than in females (Suchacki et al. 2023); thus, if we see no KO effects in males, we reasoned that we were unlikely to find them in females. Our RNAseq data also find no transcriptional signatures of altered sphingolipid metabolism in females, so we couldn’t justify the added cost of lipidomics in the females.

4.	There is no Supplementary Table 5 or 6, so I could not assess the results in section 2.9.
Response: We are unsure why you did not have access to these tables (they were included in the pdf generated by our submission). The data are included in the same format in our revised manuscript.

5.	Figure 6: the bone in this figure comes out of nowhere. Is it really necessary?
Response: We intended to highlight previous literature reporting that BMAT is a major source of adiponectin during CR (Cawthorn et al. 2014, Scheller et al. 2016). However, we agree that our study did not address this and so have updated the figure to focus only on our present findings.

**Other revisions made in addition to addressing the reviewers’ comments:**

In addition to the above updates, we have updated the myofibre cross-sectional area (CSA) analyses shown in Figure S10. In our original submission, we calculated the average CSA for each mouse and compared these averages between the different groups (sex/diet/genotype). However, we realised that, within each mouse, the myofibre CSAs are not normally distributed. Therefore, it is more appropriate to calculate the median for each mouse and to compare these medians between the different groups. This is consistent with our analyses of adipocyte sizes now shown in updated Figure 3 and Figure S3.

Figure S10 has been updated with the median CSA data. The results do not change our interpretation or conclusions.

References Cited:

Cawthorn, W. P., E. L. Scheller, B. S. Learman, S. D. Parlee, B. R. Simon, H. Mori, . . . O. A. MacDougald (2014). "Bone Marrow Adipose Tissue Is an Endocrine Organ that Contributes to Increased Circulating Adiponectin during Caloric Restriction." Cell Metabolism **20**(2): 368-375. doi:10.1016/j.cmet.2014.06.003

Daquinag, A. C., Z. Gao, C. Fussell, L. Immaraj, R. Pasqualini, W. Arap, . . . M. G. Kolonin (2021). "Fatty acid mobilization from adipose tissue is mediated by CD36 posttranslational modifications and intracellular trafficking." JCI Insight **6**(17). doi:10.1172/jci.insight.147057

Derous, D., S. E. Mitchell, C. L. Green, L. Chen, J. J. Han, Y. Wang, . . . A. Douglas (2016). "The effects of graded levels of calorie restriction: VI. Impact of short-term graded calorie restriction on transcriptomic responses of the hypothalamic hunger and circadian signaling pathways." Aging (Albany NY).

Derous, D., S. E. Mitchell, C. L. Green, Y. Wang, J. D. Han, L. Chen, . . . A. Douglas (2016). "The effects of graded levels of calorie restriction: VII. Topological rearrangement of hypothalamic aging networks." Aging (Albany NY) **8**(5): 917-932. doi:10.18632/aging.100944

Derous, D., S. E. Mitchell, C. L. Green, Y. Wang, J. D. J. Han, L. Chen, . . . J. R. Speakman (2017). "The Effects of Graded Levels of Calorie Restriction: X. Transcriptomic Responses of Epididymal Adipose Tissue." J Gerontol A Biol Sci Med Sci. doi:10.1093/gerona/glx101

Derous, D., S. E. Mitchell, L. Wang, C. L. Green, Y. Wang, L. Chen, . . . J. R. Speakman (2017). "The effects of graded levels of calorie restriction: XI. Evaluation of the main hypotheses underpinning the life extension effects of CR using the hepatic transcriptome." Aging (Albany NY) **9**(7): 1770-1824. doi:10.18632/aging.101269

Goto, T., J. Y. Lee, A. Teraminami, Y. I. Kim, S. Hirai, T. Uemura, . . . T. Kawada (2011). "Activation of peroxisome proliferator-activated receptor-alpha stimulates both differentiation and fatty acid oxidation in adipocytes." J Lipid Res **52**(5): 873-884. doi:10.1194/jlr.M011320

Green, C. L., S. E. Mitchell, D. Derous, Y. Wang, L. Chen, J. J. Han, . . . J. R. Speakman (2017). "The effects of graded levels of calorie restriction: IX. Global metabolomic screen reveals modulation of carnitines, sphingolipids and bile acids in the liver of C57BL/6 mice." Aging Cell **16**(3): 529-540. doi:10.1111/acel.12570

Green, C. L., Q. A. Soltow, S. E. Mitchell, D. Derous, Y. Wang, L. Chen, . . . J. R. Speakman (2018). "The Effects of Graded Levels of Calorie Restriction: XIII. Global Metabolomics Screen Reveals Graded Changes in Circulating Amino Acids, Vitamins, and Bile Acids in the Plasma of C57BL/6 Mice." J Gerontol A Biol Sci Med Sci. doi:10.1093/gerona/gly058

Hudak, C. S. and H. S. Sul (2013). "Pref-1, a gatekeeper of adipogenesis." Front Endocrinol (Lausanne) **4**: 79. doi:10.3389/fendo.2013.00079

Kraus, W. E., M. Bhapkar, K. M. Huffman, C. F. Pieper, S. Krupa Das, L. M. Redman, . . . L. Fontana (2019). "2 years of calorie restriction and cardiometabolic risk (CALERIE): exploratory outcomes of a multicentre, phase 2, randomised controlled trial." The Lancet Diabetes & Endocrinology **7**(9): 673-683. doi:10.1016/S2213-8587(19)30151-2

Liu, Q., B. Yuan, K. A. Lo, H. C. Patterson, Y. Sun and H. F. Lodish (2012). "Adiponectin regulates expression of hepatic genes critical for glucose and lipid metabolism." Proceedings of the National Academy of Sciences of the United States of America **109**(36): 14568-14573. doi:10.1073/pnas.1211611109

Lusseau, D., S. E. Mitchell, C. Barros, D. Derous, C. Green, L. Chen, . . . J. R. Speakman (2015). "The effects of graded levels of calorie restriction: IV. Non-linear change in behavioural phenotype of mice in response to short-term calorie restriction." Sci Rep **5**: 13198. doi:10.1038/srep13198

Maeda, N., I. Shimomura, K. Kishida, H. Nishizawa, M. Matsuda, H. Nagaretani, . . . Y. Matsuzawa (2002). "Diet-induced insulin resistance in mice lacking adiponectin/ACRP30." Nature Medicine **8**(7): 731-737.

Mauvais-Jarvis, F., A. P. Arnold and K. Reue (2017). "A Guide for the Design of Pre-clinical Studies on Sex Differences in Metabolism." Cell Metab **25**(6): 1216-1230. doi:10.1016/j.cmet.2017.04.033

Mitchell, S. E., C. Delville, P. Konstantopedos, D. Derous, C. L. Green, L. Chen, . . . J. R. Speakman (2015). "The effects of graded levels of calorie restriction: III. Impact of short term calorie and protein restriction on mean daily body temperature and torpor use in the C57BL/6 mouse." Oncotarget **6**(21): 18314-18337. doi:10.18632/oncotarget.4506

Mitchell, S. E., C. Delville, P. Konstantopedos, D. Derous, C. L. Green, Y. Wang, . . . J. R. Speakman (2016). "The effects of graded levels of calorie restriction: V. Impact of short term calorie and protein restriction on physical activity in the C57BL/6 mouse." Oncotarget. doi:10.18632/oncotarget.8158

Mitchell, S. E., C. Delville, P. Konstantopedos, J. Hurst, D. Derous, C. Green, . . . J. R. Speakman (2015). "The effects of graded levels of calorie restriction: II. Impact of short term calorie and protein restriction on circulating hormone levels, glucose homeostasis and oxidative stress in male C57BL/6 mice." Oncotarget **6**(27): 23213-23237. doi:10.18632/oncotarget.4003

Mitchell, S. E., Z. Tang, C. Kerbois, C. Delville, D. Derous, C. L. Green, . . . J. R. Speakman (2017). "The effects of graded levels of calorie restriction: VIII. Impact of short term calorie and protein restriction on basal metabolic rate in the C57BL/6 mouse." Oncotarget **8**(11): 17453-17474. doi:10.18632/oncotarget.15294

Mitchell, S. E., Z. Tang, C. Kerbois, C. Delville, P. Konstantopedos, A. Bruel, . . . J. R. Speakman (2015). "The effects of graded levels of calorie restriction: I. impact of short term calorie and protein restriction on body composition in the C57BL/6 mouse." Oncotarget **6**(18): 15902-15930. doi:10.18632/oncotarget.4142

Okun, J. G., P. M. Rusu, A. Y. Chan, Y. Wu, Y. W. Yap, T. Sharkie, . . . A. J. Rose (2021). "Liver alanine catabolism promotes skeletal muscle atrophy and hyperglycaemia in type 2 diabetes." Nature Metabolism **3**(3): 394-409. doi:10.1038/s42255-021-00369-9

Onodera, T., D.-S. Kim, M.-Y. Wang, M. Virostek, S. Chen, Z. A. Kipp, . . . P. E. Scherer (2025). "A comparison of adiponectin-deficient mice reveals the fundamental role of intracellular adiponectin." bioRxiv: 2025.2007.2012.664095. doi:10.1101/2025.07.12.664095

Qiao, L., B. Kinney, J. Schaack and J. Shao (2011). "Adiponectin inhibits lipolysis in mouse adipocytes." Diabetes **60**(5): 1519-1527. doi:10.2337/db10-1017

Qiao, L., C. Zou, D. R. van der Westhuyzen and J. Shao (2008). "Adiponectin reduces plasma triglyceride by increasing VLDL triglyceride catabolism." Diabetes **57**(7): 1824-1833. doi:10.2337/db07-0435

Scheller, E. L., A. A. Burr, O. A. MacDougald and W. P. Cawthorn (2016). "Inside out: Bone marrow adipose tissue as a source of circulating adiponectin." Adipocyte **5**(3): 251-269. doi:<https://doi.org/10.1080/21623945.2016.1149269>

Suchacki, K. J., B. J. Thomas, Y. M. Ikushima, K.-C. Chen, C. Fyfe, A. A. S. Tavares, . . . W. P. Cawthorn (2023). "The effects of caloric restriction on adipose tissue and metabolic health are sex- and age-dependent." eLife **12**: e88080. doi:<https://doi.org/10.7554/eLife.88080>

Syamsunarno, M. R. A. A., T. Iso, H. Hanaoka, A. Yamaguchi, M. Obokata, N. Koitabashi, . . . M. Kurabayashi (2013). "A Critical Role of Fatty Acid Binding Protein 4 and 5 (FABP4/5) in the Systemic Response to Fasting." PLOS ONE **8**(11): e79386. doi:10.1371/journal.pone.0079386

Virtue, S. and A. Vidal-Puig (2021). "GTTs and ITTs in mice: simple tests, complex answers." Nat Metab **3**(7): 883-886. doi:10.1038/s42255-021-00414-7

Zhu, M., J. Miura, L. X. Lu, M. Bernier, R. DeCabo, M. A. Lane, . . . D. K. Ingram (2004). "Circulating adiponectin levels increase in rats on caloric restriction: the potential for insulin sensitization." Experimental gerontology **39**(7): 1049-1059. doi:10.1016/j.exger.2004.03.024
