# Supplementary material for: Adiponectin exerts sex-dependent effects on lipid, amino acid, and glucose metabolism during caloric restriction
Source: PLoS Biol. 2026 Jun 18;24(6):e3003821. doi: 10.1371/journal.pbio.3003821 (PMC13278438; doi:10.1371/journal.pbio.3003821)
Supplement: Peer review history

# Editor report: Decision Letter 0

**DOI:** 10.1371/journal.pbio.3003821.r001

**Authors:** Lucas Smith

21 Aug 2025

Dear Dr Cawthorn,

Thank you for submitting your manuscript entitled "Adiponectin exerts sex-dependent effects on lipid, amino acid, and glucose metabolism during caloric restriction" for consideration as a Research Article by PLOS Biology.

Your manuscript has now been evaluated by the PLOS Biology editorial staff and I am writing to let you know that we would like to send your submission out for external peer review.

However, before we can send your manuscript to reviewers, we need you to complete your submission by providing the metadata that is required for full assessment. To this end, please login to Editorial Manager where you will find the paper in the 'Submissions Needing Revisions' folder on your homepage. Please click 'Revise Submission' from the Action Links and complete all additional questions in the submission questionnaire.

Once your full submission is complete, your paper will undergo a series of checks in preparation for peer review. After your manuscript has passed the checks it will be sent out for review. To provide the metadata for your submission, please Login to Editorial Manager (https://www.editorialmanager.com/pbiology) within two working days, i.e. by Aug 26 2025 11:59PM.

If your manuscript has been previously peer-reviewed at another journal, PLOS Biology is willing to work with those reviews in order to avoid re-starting the process. Submission of the previous reviews is entirely optional and our ability to use them effectively will depend on the willingness of the previous journal to confirm the content of the reports and share the reviewer identities. Please note that we reserve the right to invite additional reviewers if we consider that additional/independent reviewers are needed, although we aim to avoid this as far as possible. In our experience, working with previous reviews does save time.

If you would like us to consider previous reviewer reports, please edit your cover letter to let us know and include the name of the journal where the work was previously considered and the manuscript ID it was given. In addition, please upload a response to the reviews as a 'Prior Peer Review' file type, which should include the reports in full and a point-by-point reply detailing how you have or plan to address the reviewers' concerns.

During the process of completing your manuscript submission, you will be invited to opt-in to posting your pre-review manuscript as a bioRxiv preprint. Visit http://journals.plos.org/plosbiology/s/preprints for full details. If you consent to posting your current manuscript as a preprint, please upload a single Preprint PDF.

Feel free to email us at plosbiology@plos.org if you have any queries relating to your submission.

Kind regards,

Luke

Lucas Smith, Ph.D.

Senior Editor

PLOS Biology

lsmith@plos.org

# Peer review: Decision Letter 1

**DOI:** 10.1371/journal.pbio.3003821.r002

**Authors:** Lucas Smith

13 Nov 2025

Dear Dr Cawthorn,

Thank you for your patience while your manuscript " Adiponectin exerts sex-dependent effects on lipid, amino acid, and glucose metabolism during caloric restriction" was peer-reviewed at PLOS Biology. Please accept my apologies, again, for the protracted review process for your study. As mentioned, we had a reviewer drop out fairly late in the process and so we needed to find a replacement reviewer. You will see that we ended up with four reviewers total - that is because two reviewers ended up signing on when I was looking for a replacement. Your study has now been evaluated by the PLOS Biology editors, an Academic Editor with relevant expertise, and by these independent reviewers.

In light of the reviews, which you will find at the end of this email, we would like to invite you to revise the work to thoroughly address the reviewers' reports.

As you will see below, the reviewers find the study to be generally interesting but they have each highlighted aspects of the work that should be developed further to strengthen the study before publication. We think the reviewer comments should be thoroughly addressed, and that you will need to validate the knockout model and provide additional data to support the conclusions and flesh out the mechanistic characterizations provided here.

Given the extent of revision needed, we cannot make a decision about publication until we have seen the revised manuscript and your response to the reviewers' comments. Your revised manuscript is likely to be sent for further evaluation by all or a subset of the reviewers.

In addition to these revisions, you will need to complete some formatting changes, which you will receive in a follow up email. A member of our team will be in touch with a set of requests shortly.

We expect to receive your revised manuscript within 3 months. Please email us (plosbiology@plos.org) if you have any questions or concerns, or would like to request an extension.

At this stage, your manuscript remains formally under active consideration at our journal; please notify us by email if you do not intend to submit a revision so that we may withdraw it.

**IMPORTANT - SUBMITTING YOUR REVISION**

Your revisions should address the specific points made by each reviewer. Please submit the following files along with your revised manuscript:

1. A 'Response to Reviewers' file - this should detail your responses to the editorial requests, present a point-by-point response to all of the reviewers' comments, and indicate the changes made to the manuscript.

*NOTE: In your point-by-point response to the reviewers, please provide the full context of each review. Do not selectively quote paragraphs or sentences to reply to. The entire set of reviewer comments should be present in full and each specific point should be responded to individually, point by point.

You should also cite any additional relevant literature that has been published since the original submission and mention any additional citations in your response.

2. In addition to a clean copy of the manuscript, please also upload a 'track-changes' version of your manuscript that specifies the edits made. This should be uploaded as a "Revised Article with Changes Highlighted" file type.

*Re-submission Checklist*

When you are ready to resubmit your revised manuscript, please refer to this re-submission checklist: https://plos.io/Biology_Checklist

To submit a revised version of your manuscript, please go to https://www.editorialmanager.com/pbiology/ and log in as an Author. Click the link labelled 'Submissions Needing Revision' where you will find your submission record.

Please make sure to read the following important policies and guidelines while preparing your revision:

*Published Peer Review*

Please note while forming your response, if your article is accepted, you may have the opportunity to make the peer review history publicly available. The record will include editor decision letters (with reviews) and your responses to reviewer comments. If eligible, we will contact you to opt in or out. Please see here for more details:

https://blogs.plos.org/plos/2019/05/plos-journals-now-open-for-published-peer-review/

*PLOS Data Policy*

Please note that as a condition of publication PLOS' data policy (http://journals.plos.org/plosbiology/s/data-availability) requires that you make available all data used to draw the conclusions arrived at in your manuscript. If you have not already done so, you must include any data used in your manuscript either in appropriate repositories, within the body of the manuscript, or as supporting information (N.B. this includes any numerical values that were used to generate graphs, histograms etc.). For an example see here: http://www.plosbiology.org/article/info%3Adoi%2F10.1371%2Fjournal.pbio.1001908#s5

*Blot and Gel Data Policy*

We require the original, uncropped and minimally adjusted images supporting all blot and gel results reported in an article's figures or Supporting Information files. We will require these files before a manuscript can be accepted so please prepare them now, if you have not already uploaded them. Please carefully read our guidelines for how to prepare and upload this data: https://journals.plos.org/plosbiology/s/figures#loc-blot-and-gel-reporting-requirements

*Protocols deposition*

To enhance the reproducibility of your results, we recommend that if applicable you deposit your laboratory protocols in protocols.io, where a protocol can be assigned its own identifier (DOI) such that it can be cited independently in the future. Additionally, PLOS ONE offers an option for publishing peer-reviewed Lab Protocol articles, which describe protocols hosted on protocols.io. Read more information on sharing protocols at https://plos.org/protocols?utm_medium=editorial-email&utm_source=authorletters&utm_campaign=protocols

Thank you again for your submission to our journal. We hope that our editorial process has been constructive thus far, and we welcome your feedback at any time. Please don't hesitate to contact us if you have any questions or comments.

Sincerely,

Luke

Lucas Smith, Ph.D.

Senior Editor

PLOS Biology

lsmith@plos.org

------------------------------------

REVIEWS:

Reviewer #1: Ikushima et al. investigated the effects of calorie restriction in adiponectin knockout (KO) mice under both ad libitum and calorie-restricted conditions, in both sexes. They compared plasma adiponectin levels, body weight, and tissue weights, and found that calorie restriction significantly increased plasma adiponectin levels. However, body mass and fat mass were not different between ad libitum and calorie-restricted groups in either male or female mice.

In glucose tolerance tests (GTT), male KO mice displayed higher glucose levels under ad libitum feeding, but lower glucose levels under calorie restriction compared with wild-type controls. Interestingly, insulin levels during the oral GTT were not altered in any group. Triglyceride (TG) clearance tests showed elevated serum TG levels in male KO mice under ad libitum conditions, accompanied by increased NEFA levels, whereas females did not show these NEFA changes.

Indirect calorimetry revealed that RER was lowered under ad libitum and increased under calorie restricted condtions. Gene expression analyses revealed that male KO mice under ad libitum feeding showed upregulation of lipogenesis-related genes, while amino acid metabolic processes were enriched in calorie-restricted KO mice.

Overall, the study demonstrated that calorie restriction lowered blood glucose levels in adiponectin KO mice probably due to higher utility of carbohydrate, enhanced fasting NEFA levels, and increased postprandial RER — particularly in males — though the magnitude of change between ad libitum and calorie restriction was smaller in females than in males. To strengthen the story in this paper, the authors should address the points below.

1. The authors should present the data of a high-fat diet feeding instead of a normal chow diet and evaluate both body-weight gain and fat mass.

2. Since pyruvate is a major substrate for gluconeogenesis in the liver and kidney, the authors should perform a pyruvate tolerance test (PTT) in both male and female KO and wild-type mice to directly evaluate gluconeogenic capacity.

3. The representation of Figure 5 and Fig. S4 should be improved for clarity. For example, for panel E, the legend and figure should clearly state which pathways are enriched in which genotype (not describe "wild-type vs KO", but clearly state "enriched in wild-type" or "enriched in KO"), rather than using the term "between WT and KO", "WT vs KO". This will make the interpretation more straightforward for readers.

4. The authors showed that male adiponectin knockout (KO) mice have elevated serum triglyceride (TG) and NEFA levels, as well as upregulated amino acid catabolic pathways in the liver and higher RER. This suggests that, despite the presence of abundant circulating lipids, the KO mice preferentially utilize carbohydrate-derived energy — likely by catabolizing amino acids to support gluconeogenesis. The authors should further explain or discuss this apparent metabolic preference in adiponectin KO mice, particularly under calorie-restricted conditions, where the mice seem to favor carbohydrate production and utilization despite having elevated circulating lipids.

5. Regarding Fig. 6, bone marrow and adipose tissue illustration is not well supported by the original data. The authors should measure adiponectin expression in bone marrow and adipose tissue in each condition.

Reviewer #2: This manuscript focused on the role of adiponectin during caloric restriction. The authors found that adiponectin KO mice do not have altered body weight and body composition in both sexes. Interestingly, in male mice, the improvement in glucose tolerance due to caloric restriction is more pronounced in adiponectin KO mice. These male adiponectin KO mice had higher fatty acids in their fasting blood, as well as impaired lipid clearance after oral lipid administration. Female adiponectin KO mice showed relatively milder alterations among all the assays. RER showed that both male and female adiponectin KO mice had a lower RER, indicating more fatty acid usage compared to carbohydrate usage.

Overall the manuscript presented interesting observations suggesting there is sexual dimorphism in the role of adiponectin during caloric restriction. However, it would be nice if the authors could provide more mechanistic insights into the sexual dimorphism in adiponectin function. I also have a few suggestions that could potentially improve the manuscript.

1. Please add validation of adiponectin knockout.

2. Other than reduced fatty acid oxidation, increased fasting free fatty acids in the circulation could also be due to altered lipolysis from the adipose tissue. Actually, RER shows the KO mice prefer fatty acids as a fuel. An in vivo lipolysis test is suggested to be done in parallel with the OLTT.

3. It would be nice to validate the genes related to fatty acid transport, oxidation, and synthesis in the liver and adipose tissue in both sexes.

4. Please also validate some of the glucose-related pathways discussed in the liver, and confirm sexual dimorphism at the molecular level.

Reviewer #3:

This investigation examined whether adiponectin mediates the metabolic effects of caloric restriction. The authors generate a novel adiponectin KO mouse model via the excision of exon 3, which is noteworthy since two prior adiponectin KO models retain exon 3 encoding the bioactive domain. A bioRxiv preprint https://doi.org/10.1101/2025.07.12.664095 suggests that those KO mice display residual effects if adiponectin vs the model that achieves a complete knockout of all three exons. The effects of adiponectin KO shown here are mild, but this could be because the mice were chow-fed. Adiponectin deficiency did not alter body mass or fat mass under CR. However, the authors show the exciting findings for how adiponectin regulates hepatic gene expression. Under ad libitum feeding, adiponectin KO alters lipid metabolism genes, and during CR adiponectin KO increases genes related to amino acid catabolism. The inclusion of both sexes is admirable and provides useful information, since females naturally have higher adiponectin levels, but in this study have similar responses to males. The phenotyping is thorough, and the RNA-seq data from the liver are informative. However, the manuscript requires additional data and clarification to strengthen the conclusions about the systemic interactions of adiponectin during CR.

Major comments

Adiponectin mRNA from exon 1 should be assessed to confirm the absence of full-length transcripts and validate the KO model. This will distinguish this model from prior partial KOs. This technical advancement should be explicitly emphasized as a strength in the manuscript.

Adipose tissue phenotype has not been analyzed in sufficient depth. Since both adiponectin and CR have large effects on adipose biology, inclusion of adipose tissue histology staining of iWAT and gWAT and assessment of adipose tissue quality are essential. The fat mass may not change, but there can be significant changes in adipose tissue function. Moreover, expression of key adipocyte lineage and lipogenic genes (Ap2, Cebpa, Pparγ, Plin1, Pref1) should be assessed to clarify adiponectin's role in adipose adaptation during CR. For these reasons, I suggest removing this statement. "Line 358. It remains less clear how KO increases fasting NEFA in CR males. This is unlikely to reflect greater lipolysis, because adiponectin KO does not alter adiposity or CR-induced fat loss."

RNA-seq findings suggest major transcriptional changes in hepatic lipid handling. Yet there is no histological assessment of the liver. Since liver size decreases under CR without concurrent reductions in triglyceride levels (Fig. S3B), liver histology is essential to corroborate the RNAseq data. This is especially important considering there were the sex differences.

Did the adiponectin KO mice lose their hair? If so, a photo should be included. Alopecia can affect thermogenesis in mice housed at ~22°C and may explain the RER results.

Minor comments

The 2012 Harvey Lodish paper also examines adiponectin KO effects on liver gene expression on a chow diet and should be referenced. They have similar findings that could be discussed/mentioned. https://doi.org/10.1073/pnas.1211611109

The term hyperadiponectinemia is typically associated with a disease state; The increase in adiponectin seen due to CR is modest vs levels associated with this clinical term.

The text reads that the lack of effect on hepatic ceramides in the KO mice is "striking." This is misleading, as ceramide modulation by adiponectin has primarily been observed under high-fat feeding. Please rephrase. It makes sense CR lowers ceramides due to decreased dietary fat and enhanced insulin sensitivity. (It is very nice to see all the data on all the different ceramide species.)

Please remove the identified outliers in Supplementary Fig. S3B and S3C, which are >3 SD from the mean, as they skew the interpretation of liver TG content.

Consider discussing the potential limitations of using mice on a chow diet instead of starting out with an obese mouse or a more physiologically challenging context for liver TG accumulation, which might better reveal the role of adiponectin in the liver.

Reviewer #4: This manuscript by Ikushima et al describes the effects of adiponectin KO on the response to CR in both males and females.

Overall comments: this is a nice paper and is easy to read and follow. It could benefit from being shortened and this could come partly from a smaller amount in interpretation in the results section. Also, I believe there is a bit of overinterpretation of small changes that occur in some measurements.

Major comments

1. Please report when you fed the CR mice, i.e morning or afternoon, as this potentially has an effect on circadian rhythms. Looking at the RER it seems like the authors have fed in the morning which is why the curves have the increase in the morning while the ad lib group increase in the evening. Previous studies have shown that feeding in the light cycle vs the dark will affect transcriptional and protein profiles. Since the authors go onto investigate the differences in transcriptional profiles, can they assure the reader that they are actual changes and not just because their circadian rhythm has been disrupted. This needs to be addressed in the discussion as a major limitation of the study that may affect interpretation of outcomes and conclusion.

2. Due to the CR feeding period being in the morning, was the fasting period the same at cull between the two groups? This could have implications for the transcriptomics above, but also the NEFA concentrations in figure 3.

3. Measuring blood/plasma insulin levels throughout a GTT is not GSIS, this is a separate assay that is done ex vivo. What you are measuring during a GTT is both release from the pancreas as well as clearance through the liver. Thus, I suggest that you rename this to plasma levels during a GTT and change the wording around this particular result.

4. I believe that too much is being made out of the very small difference seen in the oGTT curves between the genotypes and CR (only different at 15 min in males). The AUC should be the focus of the statistics and discussion.

5. Regarding the lipid tolerance test: "The ability to quickly normalize hyperlipidemia following the administration of lipids provides integrated information about intestinal lipid absorption, lipid transport via lipoproteins, and tissue-specific lipid metabolism." PMID: 33022003. Therefore, just focusing on the potential for FAO being the only proposed mechanism for the differences seen is too reductionist.

6. Regarding the RER in females: Could it not be that the female KO CR mice have increased metabolic flexibility as they are better able to use the fuel source available at the time - i.e. CHO during the day when fed and fat when fasting? Saying that, they are small changes.

7. Figure 5: why do you present the male data here but put the females in the supp figure? While I realise that space is at a premium in figures, it is highly irritating having to go back and forward between the two figures to compare. Also considering the title of the paper as well as the figure legend, being able to compare the males and females easily is needed.

Minor comments

1. Why were the GTT and Intralipid doses based on BW while the insulin was based on lean mass? Lean mass would have been the better option for at least the GTT.

2. Line 134-135 "These data confirm our previous findings that CR has a weaker effect on glucose tolerance in females than in males (5)." Is weaker really the right word here - maybe smaller or lesser effect would be better? This is relevant for the rest of the manuscript as well.

3. Supp Fig 3B and C - I don't understand why the need for the two measured, content (B) is usually what is presented in publications. And considering the large amount of data presented, reducing anything that is not necessary would be beneficial. Also why were the lipidomics only done on the males?

4. There is no Supplementary Table 5 or 6, so I could not assess the results in section 2.9.

5. Figure 6: the bone in this figure comes out of nowhere. Is it really necessary?

# Author comment: Author response to Decision Letter 2

**DOI:** 10.1371/journal.pbio.3003821.r003

9 Mar 2026

Attachment Submitted filename: Reviewer response_PBIOLOGY-D-25-02593R1_v20260306.docx

# Peer review: Decision Letter 2

**DOI:** 10.1371/journal.pbio.3003821.r004

**Authors:** Lucas Smith

17 Apr 2026

Dear Dr Cawthorn,

Thank you for your patience while we considered your revised manuscript entitled "Adiponectin exerts sex-dependent effects on lipid, amino acid, and glucose metabolism during caloric restriction" for publication as a Research Article at PLOS Biology. This revised version of your manuscript has been evaluated by the PLOS Biology editors, the Academic Editor and the four original reviewers.

Based on the reviews and after consulting with our Academic Editor, we are likely to accept this manuscript for publication, provided you satisfactorily address the data and other policy-related requests stated below my signature. While we do appreciate the point raised by Reviewer 1 and that there are differences between models, the paper focusses on caloric restriction not high fat diet, thus we do not think the additional high fat diet experiment needs to be added to this study.

As you address these items, please take this last chance to review your reference list to ensure that it is complete and correct. If you have cited papers that have been retracted, please include the rationale for doing so in the manuscript text, or remove these references and replace them with relevant current references. Any changes to the reference list should be mentioned in the cover letter that accompanies your revised manuscript.

In addition to these revisions, you may need to complete some formatting changes, which you will receive in a follow up email. A member of our team will be in touch with a set of requests shortly. If you do not receive a separate email within a few days, please assume that checks have been completed, and no additional changes are required.

We expect to receive your revised manuscript within two weeks.

To submit your revision, please go to https://www.editorialmanager.com/pbiology/ and log in as an Author. Click the link labelled 'Submissions Needing Revision' to find your submission record. Your revised submission must include the following:

- a cover letter that should detail your responses to any editorial requests, if applicable, and whether changes have been made to the reference list

- a Response to Reviewers file that provides a detailed response to the reviewers' comments (if applicable, if not applicable please do not delete your existing 'Response to Reviewers' file.)

- a track-changes file indicating any changes that you have made to the manuscript.

NOTE: If Supporting Information files are included with your article, note that these are not copyedited and will be published as they are submitted. Please ensure that these files are legible and of high quality (at least 300 dpi) in an easily accessible file format. For this reason, please be aware that any references listed in an SI file will not be indexed. For more information, see our Supporting Information guidelines:

https://journals.plos.org/plosbiology/s/supporting-information

*Published Peer Review History*

Please note that you may have the opportunity to make the peer review history publicly available. The record will include editor decision letters (with reviews) and your responses to reviewer comments. If eligible, we will contact you to opt in or out. Please see here for more details:

https://plos.org/published-peer-review-history/

*Press*

Should you, your institution's press office or the journal office choose to press release your paper, please ensure you have opted out of Early Article Posting on the submission form. We ask that you notify us as soon as possible if you or your institution is planning to press release the article.

*Protocols deposition*

To enhance the reproducibility of your results, we recommend that if applicable you deposit your laboratory protocols in protocols.io, where a protocol can be assigned its own identifier (DOI) such that it can be cited independently in the future. Additionally, PLOS ONE offers an option for publishing peer-reviewed Lab Protocol articles, which describe protocols hosted on protocols.io. Read more information on sharing protocols at https://plos.org/protocols?utm_medium=editorial-email&utm_source=authorletters&utm_campaign=protocols

Please do not hesitate to contact me should you have any questions.

Sincerely,

Ines

--

Ines Alvarez-Garcia, PhD

Senior Editor

PLOS Biology

ialvarez-garcia@plos.org

on behalf of

Lucas Smith, Ph.D.

Senior Editor

PLOS Biology

lsmith@plos.org

------------------------------------------------------------------------

ETHICS STATEMENT:

-- Thank you for providing the name of the ethics committee that reviewed and approved the animal care. However, we are missing the approval/license number - please provide it.

------------------------------------------------------------------------

DATA POLICY:

I think you are aware of the PLOS Data Policy, but we do require the data underlying all individual quantitative observations that underlie the data summarized in the figures. You can have a look here: http://journals.plos.org/plosbiology/s/data-availability. For more information, please also see this editorial: http://dx.doi.org/10.1371/journal.pbio.1001797

Please update the excel file you have deposited in DataShare and upload it in our editorial system as 'Supporting Information' and save if using exactly the following convention: S1_Data.xlsx (using an underscore). However, to mention the data in the text, please use S1 Data. If the file is too big, you can split the data in several files using the same nomenclature (eg. S2_Data, etc).

Please ensure that you provide the individual numerical values that underlie the summary data displayed in the following figure panels as they are essential for readers to assess your analysis and to reproduce it:

Fig. 1A, C-E; Fig. 2A-F; Fig. 3A-D, F, G; Fig. 4A-F; Fig. 5A, B, D-H; Fig. S2; Fig. S4 and Plot S1-4

NOTE: the numerical data provided should include all replicates AND the way in which the plotted mean and errors were derived (it should not present only the mean/average values).

Please also ensure that figure legends in your manuscript include information on where the underlying data can be found. For example, you can add at the end of each correponding figure legend the following sentence: "All the data underlying the graphs can be found in S1 Data."

Please also ensure that your Data Statement in the submission system accurately describes where your data can be found.

***In addition, please make sure you deposit the raw RNA sequencing data for KO livers at NCBI's GEO repository at this stage and make it publicly available.

------------------------------------------------------------------------

CODE POLICY

Per journal policy, if you have generated any custom code during the course of this investigation, please make it available without restrictions. Please ensure that the code is sufficiently well documented and reusable, and that your Data Statement in the Editorial Manager submission system accurately describes where your code can be found. More information on our Code Policy, what and how to share can be found here: https://journals.plos.org/plosbiology/s/code-availability

Please note that we cannot accept sole deposition of code in GitHub, as this could be changed after publication. However, you can archive this version of your publicly available GitHub code to Zenodo. Once you do this, it will generate a DOI number, which you will need to provide in the Data Accessibility Statement (you are welcome to also provide the GitHub access information). See the process for doing this here: https://docs.github.com/en/repositories/archiving-a-github-repository/referencing-and-citing-content

-------------------------------------------------------------------------

BLOT AND GEL REPORTING REQUIREMENTS:

We require the original, uncropped and minimally adjusted images supporting all blot and gel results reported in an article's figures or Supporting Information files. We will require these files before a manuscript can be accepted so please prepare and upload them now. Please carefully read our guidelines for how to prepare and upload this data: https://journals.plos.org/plosbiology/s/figures#loc-blot-and-gel-reporting-requirements

Please provide the original raw gels for Fig. 1B.

------------------------------------------------------------------------

Reviewers' comments

Reviewer #1:

1. The authors should present the data of a high-fat diet feeding instead of a normal chow diet and evaluate both body-weight gain and fat mass.

In terms of this question, it is still important to validate this model by use of HFD model as the author says "diet-induced obesity has been studied extensively".

The reviewer wonder which kind of model the author's AKO mice is similar to. It is often recognized that AKO mice shows glucose intolerance. However, Kadowaki et al. reported that AKO model that lacks exon2 and 3 lower exhibit body weight (Cell Metab. 2007 Jul;6(1):55-68.) and Scherer et al.(bioRxiv [Preprint]. 2025 Jul 14:2025.07.12.664095.) as well. So, it remains controversial whether AKO develop glucose intolerance and body weight reduction. The author's data shows that male AKO increase blood glucose level at ad libitum, but decrease under fasting. Characterization under standard HFD conditions would help clarify how this model compares to previously reported AKO lines.

Regarding other questions, well addressed.

Reviewer #2:

The authors had addressed all my concerns and I have no further comments.

Reviewer #3:

The manuscript and figures were much improved. I appreciate the efforts to add validation of the mouse model, gene expression, and other markers of lipolysis. This is a valuable body of work that describes very interesting and novel aspects of adiponectin, including sex differences.

Reviewer #4:

I believe my concerns have been addressed

# Author comment: Author response to Decision Letter 3

**DOI:** 10.1371/journal.pbio.3003821.r005

4 May 2026

Attachment Submitted filename: Reviewer_response_PBIOLOGY-D-25-02593R1_v20260306_auresp_3.docx

# Editor report: Decision Letter 3

**DOI:** 10.1371/journal.pbio.3003821.r006

**Authors:** Lucas Smith

11 May 2026

Dear Will,

Thank you for the submission of your revised Research Article "Adiponectin exerts sex-dependent effects on lipid, amino acid, and glucose metabolism during caloric restriction" for publication in PLOS Biology and thank you for addressing our last editorial request in this revision. On behalf of my colleagues and the Academic Editor, Rebecca Haeusler, I am pleased to say that we can in principle accept your manuscript for publication, provided you address any remaining formatting and reporting issues. These will be detailed in an email you should receive within 2-3 business days from our colleagues in the journal operations team; no action is required from you until then. Please note that we will not be able to formally accept your manuscript and schedule it for publication until you have completed any requested changes.

**IMPORTANT - as you address any production requests to come, please also address the following requests:

1) Thank you for providing a file with the underlying data for your paper (S1_data). Please add a sentence to every relevant figure legend, pointing readers to this file. (Ex. you can say 'the underlying data for this figure can be found in S1_data')

2) Thank you for providing me with an accession number to access your RNA-seq data on GEO. Please update your Data and Code Availability Statement in our online editorial manager system to include the accession number and make the dataset public.

3) I see your current data statement currently says 'Data from an earlier version of this manuscript have been deposited at University of Edinburgh DataShare and are available at https://doi.org/10.7488/ds/7809. Upon acceptance of this manuscript, the source data will be updated and deposited on DataShare, where it will be assigned a DOI and a CC-BY license. ' >>please do provide an updated data deposition, and update your Data and Code Availability Statement in our editorial manager system, accordingly.

4) As discussed, I have updated your supplemental 'raw-images' file with the new version you provided over email. Please do take a quick look to make sure that this change looks good.

Please take a minute to log into Editorial Manager at http://www.editorialmanager.com/pbiology/, click the "Update My Information" link at the top of the page, and update your user information to ensure an efficient production process.

PRESS

We frequently collaborate with press offices. If your institution or institutions have a press office, please notify them about your upcoming paper at this point, to enable them to help maximise its impact. If the press office is planning to promote your findings, we would be grateful if they could coordinate with biologypress@plos.org. If you have previously opted in to the early version process, we ask that you notify us immediately of any press plans so that we may opt out on your behalf.

We also ask that you take this opportunity to read our Embargo Policy regarding the discussion, promotion and media coverage of work that is yet to be published by PLOS. As your manuscript is not yet published, it is bound by the conditions of our Embargo Policy. Please be aware that this policy is in place both to ensure that any press coverage of your article is fully substantiated and to provide a direct link between such coverage and the published work. For full details of our Embargo Policy, please visit http://www.plos.org/about/media-inquiries/embargo-policy/.

Thank you again for choosing PLOS Biology for publication and supporting Open Access publishing. We look forward to publishing your study.

Sincerely,

Luke

Lucas Smith, Ph.D.

Senior Editor

PLOS Biology

lsmith@plos.org
